# Uptake and Biotransformation of the Tire Rubber-derived Contaminants 6-PPD and 6-PPD Quinone in the Zebrafish Embryo (*Danio rerio*)

**DOI:** 10.1021/acs.est.3c02819

**Published:** 2023-10-02

**Authors:** Nico Grasse, Bettina Seiwert, Riccardo Massei, Stefan Scholz, Qiuguo Fu, Thorsten Reemtsma

**Affiliations:** † Department of Analytical Chemistry, Helmholtz-Centre for Environmental Research—UFZ, Permoserstrasse 15, 04318 Leipzig, Germany; ‡ Department of Bioanalytical Ecotoxicology, Helmholtz-Centre for Environmental Research—UFZ, Permoserstrasse 15, 04318 Leipzig, Germany; § Institute for Analytical Chemistry, University of Leipzig, Linnestrasse 3, 04103 Leipzig, Germany

**Keywords:** phase II metabolism, tire and road wear particles, suspect and nontarget screening, aquatic organisms, water quality

## Abstract

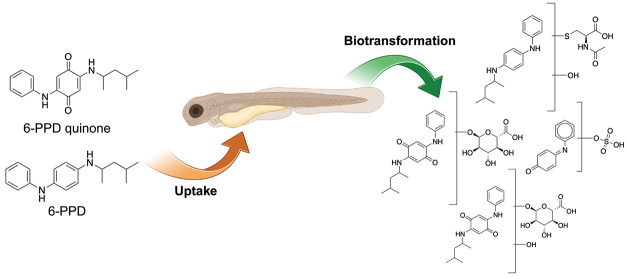

*N*-(1,3-Dimethylbutyl)-*N*′-phenyl-*p*-phenylenediamine (6-PPD) is a widely used antioxidant in tire rubber known to enter the aquatic environment via road runoff. The associated transformation product (TP) 6-PPD quinone (6-PPDQ) causes extreme acute toxicity in some fish species (e.g., coho salmon). To interpret the species-specific toxicity, information about biotransformation products of 6-PPDQ would be relevant. This study investigated toxicokinetics of 6-PPD and 6-PPDQ in the zebrafish embryo (ZFE) model. Over 96 h of exposure, 6-PPD and 6-PPDQ accumulated in the ZFE with concentration factors ranging from 140 to 2500 for 6-PPD and 70 to 220 for 6-PPDQ. A total of 22 TPs of 6-PPD and 12 TPs of 6-PPDQ were tentatively identified using liquid chromatography coupled to high-resolution mass spectrometry. After 96 h of exposure to 6-PPD, the TPs of 6-PPD comprised 47% of the total peak area (TPA), with 4-hydroxydiphenylamine being the most prominent in the ZFE. Upon 6-PPDQ exposure, >95% of 6-PPDQ taken up in the ZFE was biotransformed, with 6-PPDQ + O + glucuronide dominating (>80% of the TPA). Among other TPs of 6-PPD, a reactive *N*-phenyl-*p*-benzoquinone imine was found. The knowledge of TPs of 6-PPD and 6-PPDQ from this study may support biotransformation studies in other organisms.

## Abstract 2

A comprehensive characterization of the uptake and biotransformation of 6-PPD and 6-PPDQ in the zebrafish embryo was performed, providing a possible explanation for the relatively low sensitivity of zebrafish embryos toward 6-PPDQ.

## 1 Introduction

*N*,*N*′-Disubstituted *p*-phenylenediamines (PPDs) are added as antioxidants in tires to prevent oxidative degradation of the rubber and are themselves oxidized to quinones (PPDQs) and other transformation products (TPs).^1^ The most prominent representative of this compound class is *N*-(1,3-dimethylbutyl)-*N*′-phenyl-*p*-phenylenediamine (6-PPD). 6-PPD enters the environment via tire and road wear particles (TRWPs) produced by friction between tire and road; TRWPs are a mixture of tire tread and road surface material.^2^ TRWPs accumulate on highways and streets and enter the aquatic environment via stormwater runoff.^3–5^ It was shown recently that previously unexplained acute mortality of coho salmon (*Oncorhynchus kisutch*) in the Pacific Northwest of the United States was due to the quinone TP of 6-PPD (6-PPDQ).^5^ This emphasizes the need for identification and toxicological characterization of 6-PPD and its TPs.^5^ Analysis of representative samples of road runoff as well as surrounding waters on the U.S. West Coast revealed the widespread distribution of 6-PPDQ in this area, with concentrations in runoff samples ranging from <0.04 to 2.3 μg/L, whereas concentrations of 6-PPDQ in surrounding streams ranged from <0.02 to 0.42 μg/L.^5^ An average of 0.21 and 3.3 μg/L 6-PPDQ was detected in road snow samples in Leipzig, Germany.^6^ These environmental concentrations^1,4,6–14^ exceeded the half-maximal lethal concentrations (LC_50_) for coho salmon ranging from 41 to 95 ng/L.^5,15–17^ The particularly specific mortality of coho salmon exposed to 6-PPDQ motivated research activities on the toxicity of 6-PPDQ also to other fish species.^5,18–21^ The white spotted char (*Salvelinus leucomaenis pluvius*) is the second most sensitive species yet reported with an LC_50_ value of 0.51 μg/L.^21^ Some salmon species including rainbow trout (*Oncorhynchus mykiss*) and brook trout (*Salvelinus fontinalis*) were about 1 order of magnitude less sensitive, with LC_50_ values from 0.59 to 1.96 μg/L, respectively.^18^ Another salmonid, for example, the Arctic char (*Salvelinus alpinus*), and nonsalmonids such as the white sturgeon (*Acipenser transmontanus*), Japanese medaka (*Oryzias latipes*), and zebrafish (*Danio rerio*) with LC_50_ values >12.7 μg/L showed even greater tolerance to 6-PPDQ.^18–20^ The reason for the different sensitivities of these fish species to 6-PPDQ is still unknown. Cross-species internal concentrations and biotransformation of 6-PPDQ provide an important contribution to the characterization of species-specific toxicity. Internal concentrations of 6-PPD and 6-PPDQ have already been determined in fish species exposed under laboratory conditions, including the zebrafish embryo (ZFE).^11,22^ However, the quantification of 6-PPD and 6-PPDQ is challenging as they are very short-lived, with half-lives of 4.8 and 33 h in aqueous solutions, respectively.^20^ Besides that, knowledge of the biotransformation of 6-PPD and 6-PPDQ in fish species is still lacking.^21,22^ The phase-I-hydroxylated TP of 6-PPDQ (6-PPDQ + O) has been reported in vitro^23^ and in white-spotted char (*S. leucomaenis*), southern Asian Dolly Varden (*Salvelinus curilus*), and landlocked masu salmon (*O. masou masou*),^21^ but no data on phase II metabolism of 6-PPDQ have been reported in the peer-reviewed literature so far. Consequently, it is still unknown whether toxicokinetics (TK) plays a role in the observed species-specific 6-PPDQ toxicity. So far, only few studies have been done to investigate the mode of action of 6-PPDQ.^23^ However, in vitro data for other quinones suggested a reactive mechanism, which could explain the very high toxicity in aquatic organisms.^24^ This hypothesis is supported by Wu et al., who showed that 6-PPDQ forms DNA adducts in vivo.^25^ However, previous studies did not consider biotransformation and the role of TPs in the toxicity of 6-PPDQ.

The ZFE model has previously been used to learn about the TKs of organic contaminants because of its rapid development, small size, ease of handling, and compliance to the principle of reducing, replacing, and refining (3R) animal tests.^26–29^ The ZFE shows substantially greater tolerance to 6-PPD and 6-PPDQ compared to sensitive salmonids. For the ZFE, LC_50_ values of 442 and 133 μg/L were determined for 6-PPD and 6-PPDQ, respectively, which fall into the range of unspecific baseline toxicity.^19,30^

To address the knowledge gap of the TKs of 6-PPD and 6-PPDQ in aquatic organisms, exposure studies with ZFE were performed, combined with time-resolved quantitative analysis of internal concentrations and identification of TPs. Using this approach, the present study aimed to answer the following questions: (1) to what extent are 6-PPD and 6-PPDQ absorbed by the ZFE? (2) Which TPs are formed in the ZFE? (3) Does biotransformation have an impact on internal concentrations of 6-PPD and 6-PPDQ? This knowledge and the MS-data generated for TPs in this study support research on the biotransformation of 6-PPD and 6-PPDQ in other organisms and contribute to the understanding of the role of TKs in species-specific sensitivity of 6-PPD and 6-PPDQ.

## 2 Materials and Methods

### 2.1 Chemicals, Reagents, and Standards

All chemicals were of analytical grade and used without further purification. Methanol (>99%) and formic acid (99%) for chemical analysis were purchased from Biosolve (Valkenswaard, The Netherlands) (Table S1). The compounds for ZFE exposure experiments were dissolved in a medium defined by the International Organization for Standardization (called ISO water) containing 10 mM 4-(2-hydroxyethyl)-1-piperazineethanesulfonic acid at pH 7.4. The exact composition of ISO water is listed in the Supporting Information (Table S2). *N*-(1,3-Dimethylbutyl)-*N*′-phenyl-*p*-phenylenediamine (6-PPD, 98%, CAS 793-24-8) was purchased from abcr GmbH (Karlsruhe, Germany). The standard 6-PPD quinone (6-PPDQ) (99%, CAS 2754428-18-5) used for both ZFE exposure and calibration was provided by HPC Standards GmbH (Borsdorf, Germany). 4-Hydroxydiphenylamine (4-HDPA, 98%, CAS 122-37-2) was provided by Alfa Aesar (Kandel, Germany). Ultrapure water was obtained from a Merck Milli-Q Integral 5 system (Merck, Darmstadt, Germany).

The stock solutions of 6-PPD and 6-PPDQ were freshly prepared in methanol (MeOH) before every exposure experiment. Therefore, a 1 mg/mL solution was prepared in MeOH and then sonicated for 30 min at room temperature. The stock solutions were stored at −20 °C. To monitor the conversion of 6-PPD to 4-HDPA in the exposure medium, quantification of 4-HDPA was also performed. For calibration, a mixed standard solution of 6-PPDQ and 4-HDPA (the main abiotic TP of 6-PPD) and a separate solution of 6-PPD were diluted 1:100 in MeOH to obtain two working solutions of 10 μg/mL. Calibration standards were prepared from these solutions with MeOH in the range of 0.1 and 20 ng/mL. Extracted matrix from eight nonexposed ZFEs at 96 h post-fertilization (hpf) in MeOH was used to prepare matrix-matched calibration curves. These matrix-matched standards were diluted 1:1 with ultrapure water to obtain the final calibration concentrations in a range from 0.05 to 10 ng/mL.

### 2.2 Exposure of ZFEs

#### 2.2.1 Fish Husbandry

Adult wild-type zebrafish (strain UFZ-OBI, Leipzig) have been bred at UFZ for more than 13 generations. Fish were kept in 14 L aquaria, with 35 fish each with a sex distribution of 1:1 between females and males. The light–dark rhythm was 14:10 h, and the water temperature was 26 ± 1 °C. Water quality parameters (pH, water hardness, conductivity, nitrate, nitrite, ammonia, oxygen saturation) were measured biweekly. Spawning was induced by light, and the eggs were collected in glass trays covered with 3 mm mesh and artificial plants. Within 30 min after spawning, eggs were collected using a grid-covered dish and successively cleaned with ISO standard dilution water (ISO water) as specified in ISO 7346-3. Fish were cultured and used according to German and European animal protection standards and approved by the Government of Saxony, Landesdirektion Leipzig, Germany (Aktenzeichen 75-9185.64).

#### 2.2.2 Selection of Eggs

Developmental stages were identified according to Kimmel,^38^ and only four-cell stage embryos were selected to start the exposure. At the beginning of the experiments, the embryos had an age of about 4 ± 1 hpf.

#### 2.2.3 TK Experiments

In order to avoid mortality in the TK experiment, the exposure concentrations of 6-PPD and 6-PPDQ were 10- to 20-fold lower than the LC_50_.^19^ For 6-PPDQ, the nominal exposure concentrations were 37.5, 9.75, and 5.0 μg/L. Nominal exposure concentrations of 6-PPD were 18.75 and 4.68 μg/L. The exposure concentration of each chemical was confirmed via liquid-chromatography coupled with tandem mass spectrometry (LC–MS/MS). The MeOH stock solutions of 6-PPD and 6-PPDQ were diluted in ISO water to the corresponding nominal concentration with a total MeOH content of 0.1% (v/v). Twenty-five ZFEs were exposed in crystallization dishes (7.5 cm diameter), with a proportion of one embryo/mL. The exposure was conducted for 2, 4, 6, 8, 24, 48, 72, and 96 h, and vessels were incubated at 28 ± 1 °C. The same number of fertilized eggs were exposed in ISO water containing 0.1% MeOH as a negative control. Dead ZFEs were discarded daily to avoid fungal growth. Exposure medium was collected daily before and after the medium was refilled to confirm concentrations for 6-PPD and 6-PPDQ. Medium samples were stored at −20 °C until HPLC–MS/MS analysis. The uptake experiments were performed in three independent experiments for 6-PPD and 6-PPDQ. For each experiment, two technical replicates per compound and life stage were prepared and analyzed.

#### 2.2.4 Chemical Stability Assessment

For stability assessment, exposure media were supplemented with chemicals but without ZFE. The nominal concentration of 6-PPD and 6-PPDQ in the exposure media was 10 μg/L, while the analytically determined starting concentrations were 1.23 and 7.91 μg/L, respectively. Rapid decrease of 6-PPD concentration in solution occurred during the exposure experiments, with 55% loss over 24 h in the pure exposure medium and 75% loss in the exposure medium with ZFE. 6-PPDQ was more stable with 5 and 30% decrease, respectively, over 24 h (Figures S1 and S9). Therefore, in order to compensate for the decline, the medium was renewed daily in exposure experiments.

#### 2.2.5 Verification of the Influence of Adsorption Effects on the Detected Internal Concentration

To investigate how much 6-PPD or 6-PPDQ remains after washing with ISO water, a short-term exposure (less than 30 s) of ZFE at 72 hpf with 1 mg/L 6-PPD and 6-PPDQ was conducted according to Brox et al.^31^ In this experiment, it was assumed that such a short exposure time would prevent significant uptake of the test compounds from the embryonic body. In order to compare the results of short-term exposure with those of 72 h of exposure, we converted the data into relative concentrations. Additionally, dechorionated ZFEs were compared with ZFEs with intact chorion after 24 h of exposure using 10 μg/L exposure solutions (Supporting Information Section 7) to assess the influence of the adsorption to chorion and distribution into the perivitelline space (PVS) on the total concentration detected in the ZFE.

### 2.3 Sample Preparation for Chemical Analysis

Exposed ZFEs were collected in a FastPrep tube (2 mL) filled with glass beads (8 ZFEs per replicate). ZFEs were collected with chorion until 8 h of exposure, because dechorionation is technically impossible for such small embryos at this stage. At subsequent time points (24, 48, 72, and 96 hpf), embryos were manually dechorionated if ZFE had not hatched naturally. The exposure solution was removed, and the ZFEs were washed three times with ISO water. After all, the washing solution was removed, and the ZFEs were frozen in liquid nitrogen and stored at −80 °C until further processing. The analytes were extracted from the ZFE using MeOH according to Halbach et al. (2020).^28^ Matrix-matched calibration was performed with extracts of ZFE of 96 hpf for quantitative analysis by liquid chromatography coupled to high-resolution mass spectrometry (LC-HRMS) for 6-PPD, 6-PPDQ, and 4-HDPA available as pure reference compounds. Matrix effects in extracts of ZFE of 24 hpf differed by 0.65, 20, and 6% for 6-PPDQ, 6-PPD, and 4-HDPA from those of 96 hpf (Figure S6). Therefore, the calibration in a 96 hpf matrix was applied to all extracts. The embryos were then homogenized using a FastPrep homogenizer (MP Biomedicals, USA) for 20 s at a rate of 6.5 m/s. In the next step, the samples were sonicated for 30 min at room temperature. Afterward, the homogenates were centrifuged (13,000 rpm, 4 °C, 15 min), and 150 μL of the supernatants were transferred to HPLC glass vials containing 300 μL inserts. The extracts were stored at −20 °C until chemical analysis.

### 2.4 Chemical Analysis

#### 2.4.1 Determination of External and Internal Concentrations

Concentrations of 6-PPD and 6-PPDQ in the ZFE extracts and the exposure media were determined by using LC–MS/MS. To determine the concentration of 4-HDPA formed by 6-PPD in the exposure medium, 4-HDPA was quantified as well. A quantitative analysis of 6-PPD, 6-PPDQ, and 4-HDPA was performed using an Agilent 1290 HPLC system (Agilent) equipped with an Atlantis T3 C_18_-phase column (2.1 mm × 50 mm, 3 μm; Waters) with an Atlantis T3 Security Guard column (2.1 × 10 mm, Waters) connected to a QTRAP 5500 (SCIEX) triple quadrupole mass spectrometer by multiple reaction monitoring. Transitions and HPLC conditions are given in the Supporting Information (Table S7). Extracts of exposed ZFE were diluted to the calibration range of the analytes, respectively. For injection, the final samples contained a water/MeOH-ratio of 1:1 (v/v). A matrix-matched calibration was performed using standards prepared in the ZFE matrix. The sample preparation of the matrix-matched calibration standards was conducted directly before chemical analysis to minimize the degradation of the analytes.

#### 2.4.2 Analysis of TPs with UPLC-QTOF-MS

For the identification of TPs, ZFE extracts were analyzed by ultraperformance liquid chromatography time-of-flight mass spectrometry (UPLC-TOF-MS) using a ACQUITY UPLC I-Class system (Waters) equipped with a HSS T3 column (100 mm × 2.1 mm, 1.7 μm) coupled to a XEVO G2S mass spectrometer (Waters). The detailed instrumental conditions are according to Seiwert et al. 2022,^1^ and the corresponding method can be found in the Supporting Information (Table S6). The extracts were injected without further dilution in a water/MeOH ratio of 1:1 (v/v).

### 2.5 Data Analysis

#### 2.5.1 Identification of TPs Using Suspect- and Nontarget screening

TPs were identified using both MarkerLynx (Waters, version 4.1) and UNIFI (Waters, version 1.8.2.169). To get a first overview of occurring TPs, data were evaluated using the UNIFI software from Waters using a suspect list and the parameters listed in Table S13. To detect a wider range of TPs, a nontarget screening approach using MarkerLynx was used. More details on parameter settings can be found in the Supporting Information (Table S14).

UPLC–MS data were evaluated in a retention time window of 1–10 min and a mass range of 50–1200. The peak picking was performed using MarkerLynx with a 0.1 min deviation in retention time and a 0.01 Da deviation in the exact mass. Chemical formulas were generated using a mass tolerance of 5 ppm and elemental composition of C (0–100), H (0–100), N (0–20), O (0–20), S (0–20), and Na (0–2). In addition, fragment ions were considered for structure elucidation based on possible (bio)transformations that may happen to the parent compounds 6-PPD and 6-PPDQ. The results were exported to Excel (Microsoft), and all further statistical analysis steps were performed there. Newly appeared peaks in the exposed ZFE extracts compared to the unexposed ZFE samples and the solvent blanks were selected as candidate TPs.

#### 2.5.2 Determination of the Formation Kinetics of TPs

The previously identified TPs were incorporated into a potential target list to detect their formation kinetics in the exposed organisms. The retention times and exact masses of the molecular ions of the TPs were used in the TargetLynx method. This method was applied to monitor the time course of the peak areas of the identified TPs. For this purpose, the samples generated in the uptake experiments (Section 2.2.3) were analyzed by UPLC-QTOF-MS, and the data obtained were analyzed via TargetLynx.

## 3 Results and Discussion

### 3.1 Impact of Adsorption and PVS on Internal Concentrations in the ZFE

ZFE exposed to 6-PPD and 6-PPDQ showed fourfold and sixfold higher total concentrations, respectively, if extracted with their chorion compared to ZFE extracted without their chorion (Supporting Information Section 7 and Figure S6). The ZFE were exposed to 10 μg/L (nominal concentration). The measured concentrations in the exposure medium were 1.23 μg/L for 6-PPD and 7.91 μg/L for 6-PPDQ. For better comparability, the data were expressed as relative concentrations. Thus, the total relative concentration after 24 h in the ZFE with chorion was 746 ± 50 (*n* = 2), and after dechorionation, it was 210 ± 34 (*n* = 2). For 6-PPDQ, the total relative concentration with chorion was 209 ± 34 (*n* = 2), while this value decreased after dechorionation to 33.42 ± 0.33 (*n* = 2). Thus, the adsorption of both compounds to the chorion as well as their distribution into the PVS led to an overestimation of the internal concentration.^32^ Therefore, manual dechorionation of nonhatched ZFE was considered mandatory before homogenization and extraction.

Adsorption to the ZFE itself, however, could also bias internal concentration analysis and would be relevant both for nonhatched and hatched ZFE. A short-term (30 s) exposure of hatched ZFE (72 hpf) led to around 10% of the total 6-PPD concentration in the ZFE detected in the 72 h exposure (Figure S8). For 6-PPDQ, adsorption was relatively more important, with 22% of the total concentration found after 72 h of exposure (Figure S8). Therefore, adsorption to the surface of hatched ZFE may lead to a slight overestimation of the internal concentration.

### 3.2 Uptake Kinetics of 6-PPD and 6-PPDQ in the ZFE

**Figure 1:**
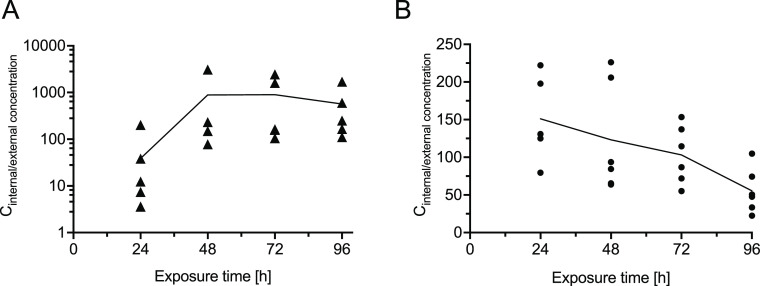
Time course of internal concentrations of 6-PPD and 6-PPDQ in ZFE exposed from 4 to 100 hpf. Data are shown as individual replicates, with means connected by a line. (A) Relative internal concentrations of 6-PPD of all experiments. Analytically determined exposure concentrations of 6-PPD were 6.3 and 1.28 μg/L, respectively. Relative internal concentrations are presented on a logarithmic scale. (B) Relative internal concentrations of 6-PPDQ of all experiments. Analytically determined exposure concentrations of 6-PPDQ were 20.9, 11.3, and 4.8 μg/L, respectively.

None of the test substances were detected in the blank samples or in extracts of nonexposed ZFE (Figure S4). Due to the high reactivity as well as partitioning and sorption tendency of 6-PPD and 6-PPDQ in the exposure system, the external concentrations were not stable over 96 h of exposure, though the exposure medium was refreshed every 24 h. Thus, internal concentrations are given as relative internal concentrations (Figure 1). This approach involved relating the determined internal concentrations to the determined external concentrations at each time of sampling. The relative internal concentration data, especially for 6-PPD, showed high standard deviations probably related to the nonconstant exposure conditions. The lower variability for 6-PPDQ concentrations is probably due to its higher chemical stability and less pronounced tendency to sorption compared to 6-PPD. Already at the beginning of 96 h exposure, the 6-PPD concentration was only 12% and the concentration of 6-PPDQ was 79% of the nominal concentration (Supporting Information Section 4). This stability difference is consistent with the previously reported half-lives of 6-PPD and 6-PPDQ in aqueous solutions (4.8 and 33 h, respectively).^20^ This makes quantitative assessments of the concentrations of both compounds challenging. In addition, the biological variability of the individuals contributed to the data scatter. Results from all experiments, including two technical replicates and three independent biological replicates, are shown in Figure 1A,B. Internal concentrations are given as relative internal concentrations.

Both compounds, 6-PPD and 6-PPDQ, are rapidly absorbed by ZFE and are thus potentially available for biotransformation. A steady state of 6-PPD concentration in the ZFE was reached after 48 h of exposure (Figure 1A). The ratio between the total internal concentration in ZFE and the concentration in the exposure medium is determined as the concentration factor (CF). Using the internal concentration of 6-PPD at 72 h, where a steady state was reached in all experiments, CFs of 6-PPD ranged from 142 to 2447 (Supporting Information Figure S11), indicating bioaccumulation in the ZFE as previously reported.^22^

In contrast to 6-PPD, 6-PPDQ did not reach a stable internal concentration (Figure 1B). Rather, a mean maximum CF of 135.8 ± 64.9 (*n* = 11) was reached between 24 and 48 h of exposure and then decreased significantly to a CF of 55.5 ± 29.84 (*n* = 6; *P* = 0.0157) after 96 h of exposure (Figure 1B). The CF at the highest internal concentration (48 h of exposure) ranged from 75 to 216. The decrease in the internal concentration of 6-PPDQ may be associated with its biotransformation reactions in the ZFE and the formation of TPs.

### 3.3 TPs and Pathways of 6-PPD and 6-PPDQ in the ZFE

#### 3.3.1 Biotransformation of 6-PPD

**Figure 2:**
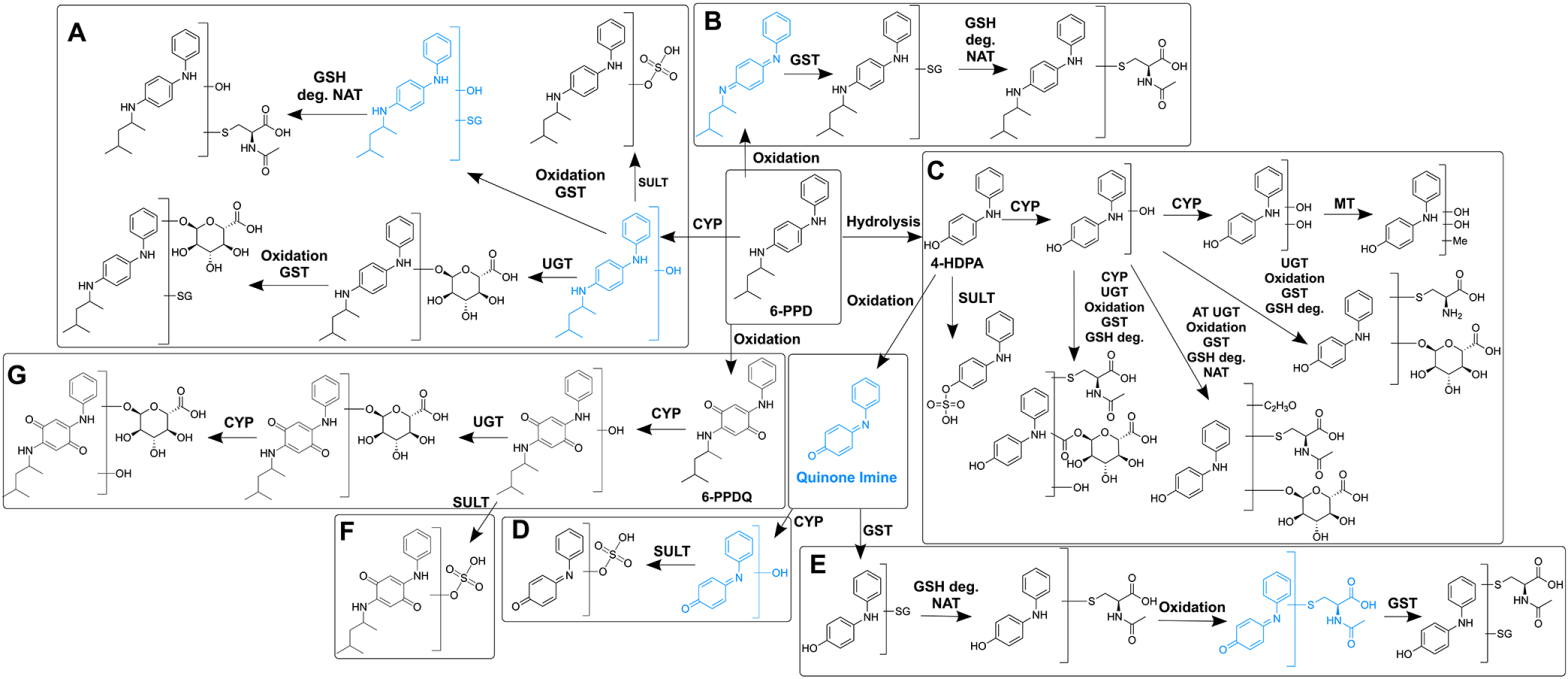
TPs detected in ZFE exposed to 6-PPD and hypothetical transformation pathways based on the structural change and putative enzymatic reactions. Proposed intermediates are marked in blue. CYP—cytochrome P450; UGT—UDP-glucuronosyltransferase; SULT—sulfotransferase; MT—methyltransferase; AT—acetyltransferase; NAT—*N*-acetyltransferase. GSH – glutathione; GST – glutathione-S-transferase; deg. – degradation. The arrows indicate the involved enzymes but not the sequence of the reactions. Detailed mass spectrometric data of TPs are given in the Excel file attached to the Supporting Information.

After 96 h of exposure, 22 putative TPs of 6-PPD were detected in exposed ZFE by suspect and nontarget screening with LC-HRMS. These TPs were tentatively identified by their exact mass and fragment ions (Excel file attached to Supporting Information) and assigned to different hypothetical biotransformation pathways A–G (Figure 2). 6-PPD was converted into 4-HDPA already in the exposure medium, indicating a standard impurity and abiotic transformation (Supporting Information Figure S3). This finding was consistent with the results of Seiwert et al. (2022) who found that 6-PPD shows high reactivity and forms numerous TPs by abiotic oxidation and hydrolysis, 4-HDPA being one among them.^1^ Therefore, it is not possible to distinguish whether 4-HDPA found in the ZFE was formed in the medium or after the uptake of 6-PPD in the ZFE.

Except for 6-PPDQ, no phase I metabolites of 6-PPD were found in the ZFE. However, the tentatively identified phase II conjugated metabolites such as glucuronides and sulfates (Figure 2, pathway A) suggest that a hydroxylated 6-PPD was formed as the intermediate. The presence of glutathione (GSH)-conjugates and their degradation products, such as cysteine and *N*-acetylcysteine adducts, indicates prior oxidative activation of 6-PPD to form a *p*-benzoquinone diamine derivative (Figure 2, pathway A). Alternatively, activation by epoxidation of the aromatic ring would also be possible.^33^ This hypothesis of an intermediate epoxide being formed is supported by the presence of both a hydroxyl group and a GSH residue at the same time. The same might be true for 4-HDPA (Figure 2, pathway C).

Additionally, two phase II conjugations occurred simultaneously on the same molecule, as in pathways A and B, where both glucuronic acid conjugation and GSH conjugation were observed (Figure 2). Besides glucuronides, GSH-, cysteine-, *N*-acetylcysteine-, and sulfate-adducts, no other phase II metabolites were observed, such as taurine- and carnitine conjugates previously described for other chemicals in the ZFE.^27^ A few TPs of 6-PPDQ were also found in extracts of ZFEs exposed to 6-PPD. The hydroxy metabolite (6PPDQ + O), glucuronides (6-PPDQ + O + Gluc; 6-PPDQ + 2O + Gluc), and a sulfated TP (6-PPDQ + O + SO_3_) were detected (Figure 2, pathways F and G).

In total, 12 metabolites of 4-HDPA were found, mono- and dihydroxylated 4-HDPA being two among them. Phase II metabolization to *N*-acetylcysteine-, cysteine-, glucuronide-, and GSH-adducts occurred for 4-HDPA (Figure 2, pathways C–E). In addition, oxidation of 4-HDPA to a highly reactive quinone imine was observed, which was tentatively identified as a sulfate conjugate in both positive and negative ion modes (Figure 2, pathway D). No other phase II metabolites of *N*-phenyl-*p*-benzoquinone imine (4-HDPA-quinone imine) were found. Quinone imines have been reported to behave similarly as quinones and cause mitochondrial dysfunction and hepatotoxicity.^34–36^ A similar metabolization pattern has been described for acetaminophen, also forming a reactive quinone imine, as from 4-HDPA, that has been shown to react with biomolecules.^37^ However, without reference compounds and the associated toxicity data, no conclusion can be drawn about whether 4-HDPA quinone imine significantly contributes to the toxicity observed upon exposure to 6-PPD.

#### 3.3.2 Biotransformation of 6-PPDQ

**Figure 3:**
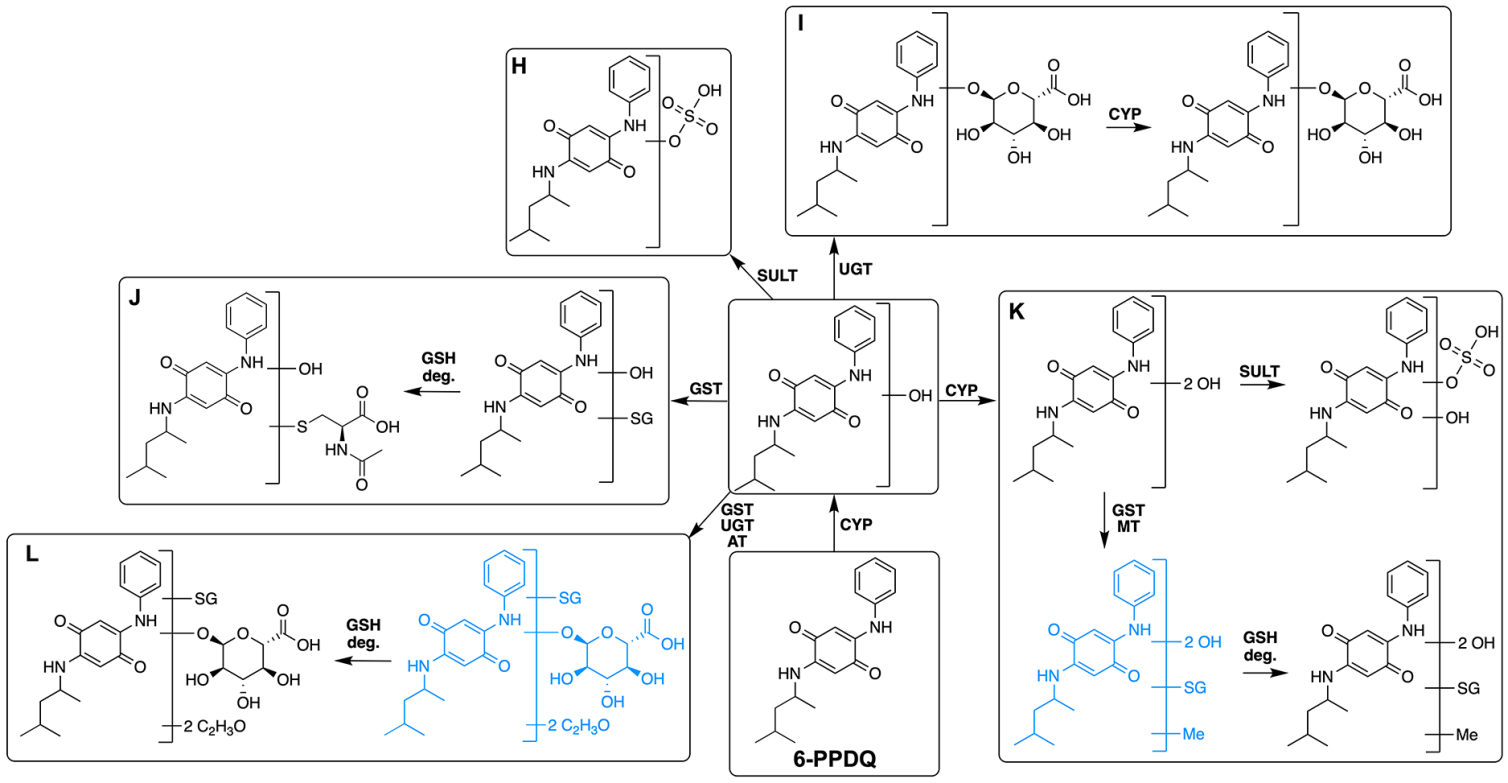
TPs detected in ZFEs exposed to 6-PPDQ and proposed transformation pathway based on chemical structures and known enzymatic reactions. The arrows indicate the potentially involved enzymes but not the sequence of reactions. Proposed intermediates are marked in blue. CYP—cytochrome P450; NAT—*N*-acetyltransferase; UGT—UDP-glucuronosyltransferase; SULT—sulfotransferase; QR—quinone reductase. GSH – glutathione; GST – glutathione-S-transferase; deg. – degradation. Detailed mass spectrometric data of TPs are given in the Excel file attached to Supporting Information.

In the extracts of ZFE exposed to 6-PPDQ, a total of 12 TPs related to the core structure of 6-PPDQ were tentatively identified (Figure 3 and Section 12 in Supporting Information). Two phase I metabolites, for example, monohydroxylated and dihydroxylated TPs, were detected. In the peer-reviewed literature, 6-PPDQ + O is the only metabolite of 6-PPDQ reported thus far.^21,23^ Different phase II reactions were assigned for the monohydroxylated 6-PPDQ (Figure 3, pathways H–L). Additionally, glucuronide-, *N*-acetylcysteine-, sulfate-, and GSH-adducts of 6-PPDQ were detected (Figure 3). Since all TPs detected exhibit the quinone motif, no indication for reductive metabolization of 6-PPDQ was found. Consequently, these TPs might have preserved the ability for redox cycling and the associated formation of reactive-oxygen species.^24^ However, the specific relative contribution of these properties to quinone toxicity is influenced by the chemical structure, particularly substituent effects. Based on the abundance of phase II metabolites and the tolerance of ZFE to 6-PPDQ, it is more likely that their formation contributes mainly to detoxification.

### 3.4 Kinetics of Biotransformation of 6-PPD and 6-PPDQ

#### 3.4.1 Relative Importance of 6-PPD, 6-PPDQ, and Their TPs in the ZFE

**Figure 4:**
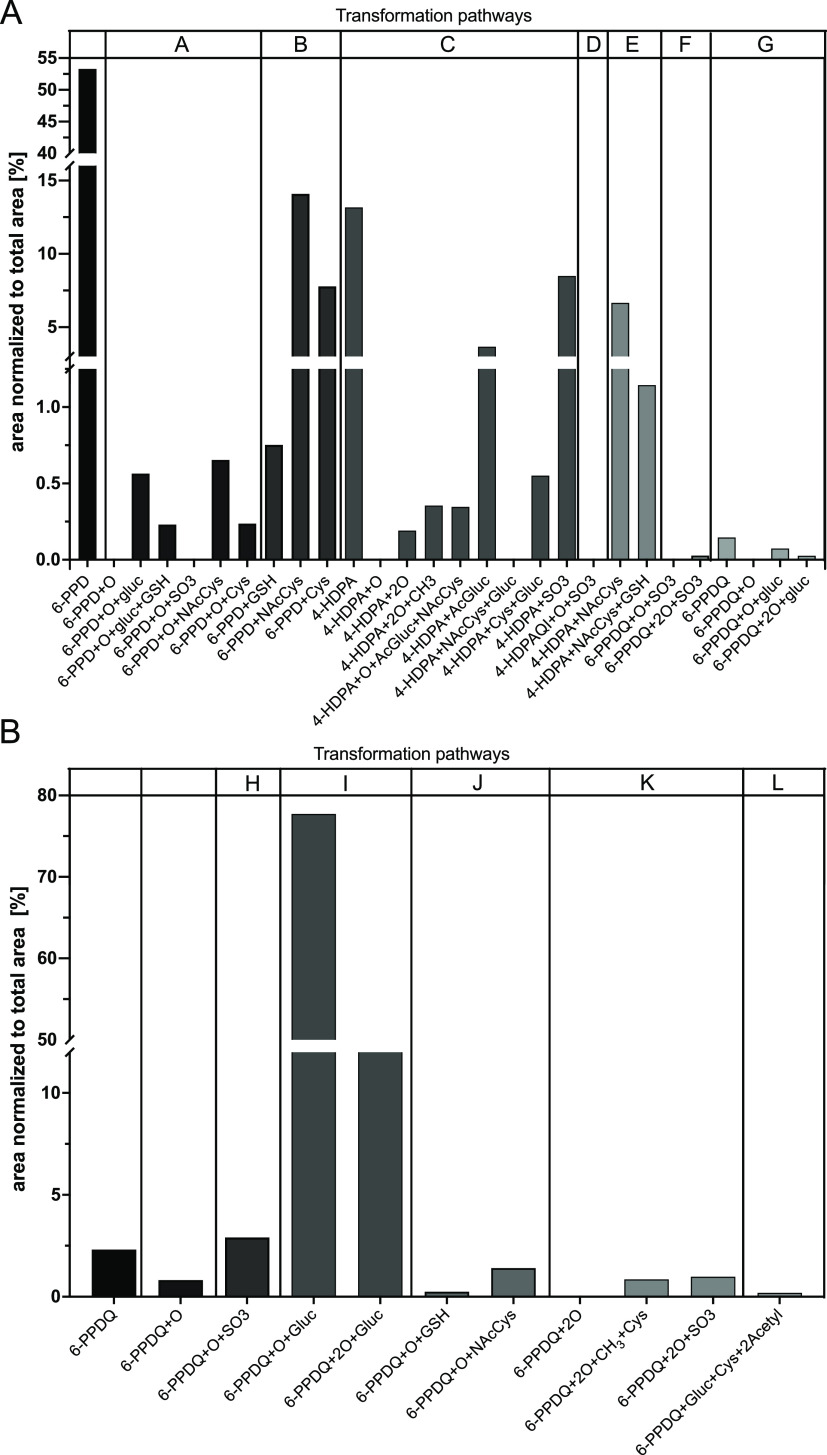
Distribution of TPs of 6-PPD in extracts of ZFE exposed to 6-PPD after 96 h of exposure. (A) Peak area of 6-PPD and its TPs normalized to the TPA. ZFE were only exposed to 6-PPD. TPs based on 6-PPD, 4-HDPA, and 6-PPDQ are shown separately. (B) Distribution of TPs of 6-PPDQ in extracts of ZFE exposed to 6-PPDQ after 96 h of exposure. The exposure was started at 4 ± 1 hpf. Gluc—glucuronide; GSH—glutathione; AcGlu—acylglucuronide; Cys—cysteine; NAcCys—*N*-acetyl-cysteine. TPA is related to the sum of all of the TPs detected and their corresponding parental compounds. Letters refer to the transformation pathways indicated in Figure 3.

Since no reference standards of the numerous TPs of 6-PPD and 6-PPDQ were commercially available, only a semiquantitative assessment based on peak areas could be conducted to estimate the relative contribution of TPs to the exposure. Therefore, the peak areas of TPs and the parent compounds were normalized to the combined total peak area (TPA) of all analytes, including the parent compound and its TPs (Figure 4). Semiquantitative data were arranged according to the hypothetical transformation pathways in Figures 2 and 3. The 22 TPs of parent compound 6-PPD accounted for 47% of the TPA after 96 h of exposure (Figure 4A). These data might indicate that half of the initial amount of 6-PPD taken up by the ZFE was transformed within 96 h of exposure. The TPs with the highest relative TPA contribution were 6-PPD + NAcCys, 4-HDPA, 4-HDPA + SO_3_, 6-PPD + Cys, 4-HDPA + NAcCys, and 4-HDPA + AcGluc (Figure 4A). These TPs correspond to pathways A–C (Figure 2). TPs of 6-PDDQ found in extracts of ZFE exposed to 6-PPD were 6-PPDQ + O + Gluc, 6-PPDQ + 2O + Gluc, and 6-PPDQ + O + SO_3_ (Figure 4A) (pathways F and G). However, the contribution of 6-PPDQ and its TPs to the TPA was almost negligible (0.28%). This indicates that the formation of 6-PPDQ from 6-PPD is not a relevant process in the ZFE.

In extracts of ZFE exposed to 6-PPDQ, most of 6-PPDQ (>95%) was transformed within 96 h of exposure (Figure 4B). This explains the observed decline in the internal 6-PPDQ concentration after 48 h of exposure. The extensive biotransformation and decrease of 6-PPDQ in ZFE might explain the greater tolerance of ZFE to 6-PPD. After 96 h of exposure, approximately 80% of the TPA of 6-PPDQ and its TPs was attributed to 6-PPDQ + O + Gluc. Furthermore, 6-PPDQ + 2O + Gluc, which is assigned to the same pathway (I), contributed to 12% of the TPA. These results suggest that the pathway I (Figure 3, pathway I) is likely the most important in 6-PPDQ biotransformation in the ZFE. The peak area of 6-PPDQ + 2O + SO_3_ (pathway H) shows a proportion of 3% to the TPA, indicating a minor role in the transformation of 6-PPDQ in the ZFE. The remaining 4.7% of TPA are attributed to other TPs formed from 6-PPDQ (Figure 3). Hiki et al. reported on 6-PPDQ + O tissue concentrations using a semiquantitative approach^23^ in the brain and gills of three salmonids.^21^ In this previous study, no significant differences in tissue concentrations were found for this phase I metabolite across sensitive and tolerant species. Our data indicate extensive biotransformation of 6-PPDQ in the ZFE forming phase II metabolites from phase I hydroxylated 6-PPDQ such as 6-PPDQ + O + glucuronide and 6-PPDQ + 2O + glucuronide. Thus, phase I metabolism is relevant for the transformation of 6-PPDQ in ZFE and might contribute to species-specific differences. A definite conclusion can be made only after comparative studies on biotransformation of 6-PPDQ between tolerant and sensitive species, taking the wider range of phase I and phase II metabolites into consideration.

#### 3.4.2 Time Course of TPs of 6-PPD and 6-PPDQ

**Figure 5:**
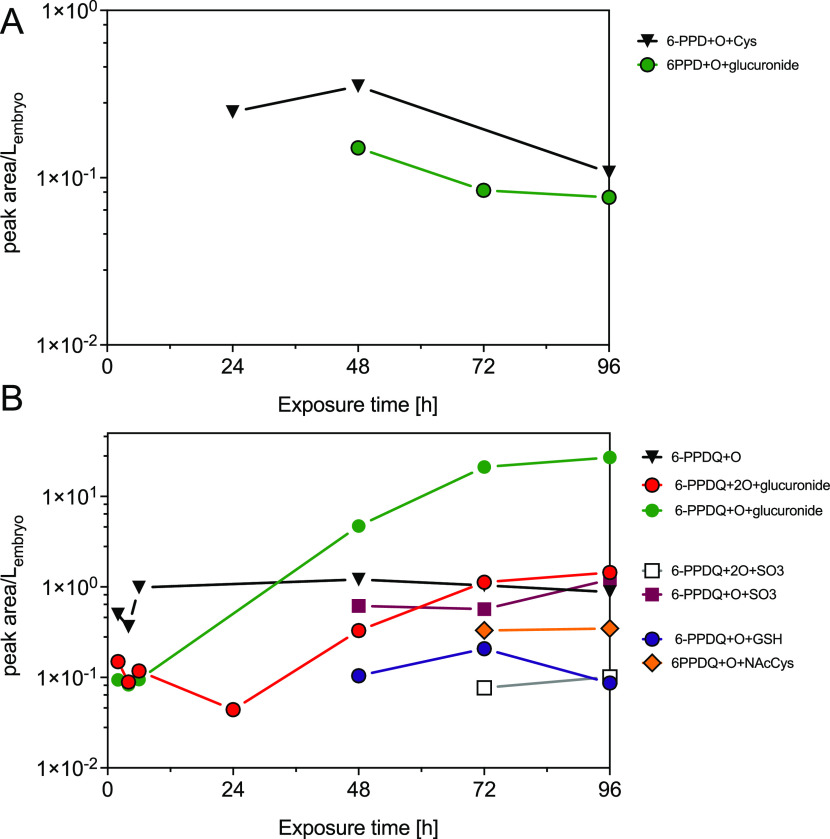
Time course of TPs of 6-PPD and 6-PPDQ. (A) Peak areas for phase II TPs of 6-PPD in ZFE over 96 h of exposure (analytically determined exposure concentration of 6.3 μg/L); (B) peak areas of phase I and phase II metabolites of 6-PPDQ over 96 h of exposure (analytically determined exposure concentration of 11.3 μg/L). Samples are as in Figure 3. Peak areas were normalized to the number of ZFE per sample and its volume at the respective life stage.

Time-resolved data were obtained for ZFE exposed to nominal concentrations of 18.75 and 9.75 μg/L of 6-PPD and 6-PPDQ, respectively (see Section 3.1; Figure 5). In extracts of ZFEs exposed to 6-PPD, the phase II metabolites 6-PPD + O + Cys and 6-PPD + O + glucuronide were detected. After 24 h of exposure, 6-PPD + O + Cys was detected. The 6-PPD + O + glucuronide occurred only after 48 h of exposure, indicating an increase in metabolic activity at more advanced life stages.

For 6-PPDQ, seven TPs were detected in the time-resolved experiment (Figure 5B), with the phase I hydroxy metabolite 6-PPDQ + O and phase II metabolites 6-PPDQ + O + glucuronide and 6-PPDQ + 2O + glucuronide detected after 2 h. Most of the 6-PPDQ absorbed by the ZFE over 96 h of exposure was transformed (94% related to TPA). 6-PPDQ + O reached a steady state after 6 h. At these early stages of development (<24 hpf), all the toxic outcomes appear to happen.^19^ This early extensive phase I biotransformation to 6-PPDQ + O suggests that the ZFE is capable of rapidly biotransforming this compound even at the developmental stage of 2 h, which is a possible explanation for its reduced sensitivity to 6-PPDQ compared to other fish species. A rapid increase in the peak area of 6-PPDQ + O + glucuronide, the main phase II metabolite of 6-PPDQ with 76% contribution to TPA, was observed between 24 and 48 h of exposure, indicating that phase II metabolism has greater relevance from this life stage onward. Correspondingly, from 48 h of exposure onward, an increase in the internal concentrations of further phase II metabolites such as 6-PPDQ + 2O + glucuronide, 6-PPDQ + O + SO_3_, 6-PPDQ + 2O + SO_3_, 6-PPDQ + NAcCys, and 6-PPDQ + GSH was observed.

## 4 Research Implications

This study indicates an extensive metabolization of 6-PPD and 6-PPDQ in the ZFE. The tentatively identified TPs of 6-PPD and 6-PPDQ in the ZFE represent the first detailed data set of the biotransformation and TKs of 6-PPD and 6-PPDQ in aquatic organisms. In total, 22 TPs of 6-PPD and 12 TPs of 6-PPDQ were tentatively identified by HRMS and assigned to hypothetical biotransformation pathways. Semiquantification of TPs based on peak areas showed that within 96 h of exposure, about 50% of 6-PPD and 95% of 6-PPDQ could be detoxified via biotransformation in the ZFE. This might explain the greater tolerance of ZFE to 6-PPDQ compared to other fish species, for example, coho salmon. The most prominent TP of 6-PPDQ was 6-PPDQ + O + glucuronide, which accounted for 80% of the TPA. However, 4-HDPA-quinone imine was detected, which may also exhibit toxic effects in the ZFE. To which extent biotransformation may contribute to the species-specific sensitivity of 6-PPDQ would need to be tested in a comparative study of the extent of biotransformation of 6-PPDQ in both tolerant and sensitive fish species. The biotransformation products identified in the ZFE model can be used as a suspect list to search for TPs in other organisms and support the assessment of the role of 6-PPDQ biotransformation in its species-specific toxicity.

## Supporting Information Available

Physicochemical properties and baseline toxicities of test chemicals; preparation of standard and exposure solutions; time course of external concentrations during 96 h of exposure; assessment of the chemical stability of 6-PPD and 6-PPDQ during 96 h of exposure without embryos; instrumental parameters of LC–MS methods; software parameters used for data analysis; raw data of internal and external concentrations; and MS/MS spectra of transformation products (PDF)
Information about retention times, *m*/*z* values of precursor and fragment ions, proposed chemical formulas, mass errors, and neutral losses of transformation products (XLSX)

## Abbreviations

4-HDPA: 4-hydroxydiphenylamine
6-PPD: *N*-(1,3-dimethylbutyl)-*N*′-phenyl-*p*-phenylenediamine
6-PPDQ: *N*-(1,3-dimethylbutyl)-*N*′-phenyl-*p*-phenylenediamine quinone
GSH: glutathione
AcGluc: acyl-glucuronide
CYP: cytochrome P450
Cys: cysteine
Gluc: glucuronide
GSH: glutathione
hpf: hours post fertilization
LC_50_: median lethal concentration of a substance that is expected to cause death in 50% of the exposed animals
NAcCys: *N*-acetylcysteine
RT: room temperature
SULT: sulfotransferase
TK: toxicokinetics
TP: transformation product
TPA: total peak area
UGT: UDP-glucuronosyltransferase
UPLC-QTOF-MS: ultraperformance liquid chromatography quadrupole-time-of-flight mass spectrometry
ZFE: zebrafish embryo
