# Supplementary material for: Uptake and Biotransformation of the Tire Rubber-derived Contaminants 6-PPD and 6-PPD Quinone in the Zebrafish Embryo (Danio rerio)
Source: Environ Sci Technol. 2023 Oct 2;57(41):15598–607. doi: 10.1021/acs.est.3c02819 (PMC10586378; doi:10.1021/acs.est.3c02819)
Supplement: Supplementary file 1 — es3c02819_si_001.pdf [file es3c02819_si_001.pdf]

Supporting information for

Uptake and biotransformation of the tire rubber-derived contaminant 6-PPD and 6-PPD quinone in the zebrafish embryo (*Danio rerio*)

Nico Grasse[a], Bettina Seiwert[a], Riccardo Massei[b], Stefan Scholz[b], Qiuguo Fu[a*#], Thorsten Reemtsma[a,c*#]

[a] Helmholtz-Centre for Environmental Research – UFZ, Department of Analytical Chemistry, Permoserstrasse 15, 04318 Leipzig, Germany.

[b] Helmholtz-Centre for Environmental Research – UFZ, Department of Bioanalytical Ecotoxicology, Permoserstrasse 15, 04318 Leipzig, Germany.

[c] University of Leipzig, Institute for Analytical Chemistry, Linnestrasse 3, 04103 Leipzig, Germany.

*Corresponding to:

Helmholtz-Centre for Environmental Research – UFZ

Department of Analytical Chemistry

Permoserstrasse 15, 04318 Leipzig, Germany

Dr. Qiuguo Fu and Prof. Dr. Thorsten Reemtsma

#Q.F. and T.R. contributed equally to this work

E-mail address: qiuguo.fu@ufz.de; thorsten.reemtsma@ufz.de

Table of content

1. CHEMICALS, SOLVENTS AND SOLUTIONS 5

2. DEVICES AND SOFTWARE 6

3. PHYSICOCHEMICAL PROPERTIES OF CHEMICALS 7

4. QUALITY ASSURANCE AND QUALITY CONTROL 8

5. 96 H EXPOSURE EXPERIMENT FOR IDENTIFICATION OF BIOTRANSFORMATION PRODUCTS 12

6. HPLC-MS/MS ANALYSIS 13

7. SOFTWARE PARAMETERS FOR IDENTIFICATION OF TRANSFORMATION PRODUCTS 20

8. THE INFLUENCE OF ADSORPTION ON INTERNAL CONCENTRATIONS 21

9. INTERNAL AND EXTERNAL CONCENTRATIONS OF 6-PPD AND 6-PPDQ IN ZEBRAFISH EMBRYOS 25

10. CALCULATION OF INTERNAL CONCENTRATIONS 31

11. CHROMATOGRAMS AND ADDITIONAL FIGURES 32

12. MS SPECTRA OF TRANSFORMATION PRODUCTS OF 6-PPD AND 6-PPDQ 34

13. REFERENCES SUPPORTING INFORMATION 63

List of figures

FIGURE S1: RECOVERY OF 6-PPD AND 6-PPDQ AFTER 24 H 8

FIGURE S2: RECOVERY OF 6-PPD AND 6-PPDQ AFTER 96 H 9

FIGURE S3: RELATIVE PROPORTION OF 4-HDPA AND 6-PPDQ IN AQUEOUS SOLUTION OF 6-PPD BEFORE AND AFTER 24 HOURS 9

FIGURE S4: EXTRACTED-ION-CHROMATOGRAMS OF 6-PPD, 4-HDPA AND 6-PPDQ IN EXPOSURE MEDIA CONTAINING 6-PPD 10

FIGURE S5: REPLICATE MEASUREMENTS OF STANDARDS OF 6-PPD AND 6-PPDQ, AND 4-HDPA 11

FIGURE S6: METHOD RECOVERIES AND MATRIX EFFECTS 19

FIGURE S7: ASSESSMENT OF THE ADSORPTION TO THE CHORION OF 6-PPD AND 6-PPDQ TO EXPOSED ZFE. 22

FIGURE S8: CONTRIBUTION OF ADSORPTION OF 6-PPD AND 6-PPDQ TO INTERNAL CONCENTRATION ANALYSIS. 23

FIGURE S9: TIME COURSE OF EXTERNAL CONCENTRATIONS OVER 96 HOURS OF EXPOSURE. 25

FIGURE S10: UPTAKE CURVES OF 6-PPDQ FROM THREE INDEPENDENT EXPERIMENTS. 29

FIGURE S11: UPTAKE CURVES OF 6-PPD FROM THREE INDEPENDENT EXPERIMENTS. 30

FIGURE S12: VOLUMES OF WHOLE EMBRYOS, EMBRYONIC BODIES AND YOLK OF ZEBRAFISH EMBRYOS 31

FIGURE S13: LC-HRMS CHROMATOGRAM OF 6-PPDQ+O+GLUCURONIDE IN EXPOSURE MEDIUM CONTAINING 37.5 µG/L 6-PPDQ 32

FIGURE S14: EXTRACTED-ION-CHROMATOGRAM OF 6-PPDQ AND 4-HDPA IN FRESHLY PREPARED EXPOSURE MEDIUM CONTAINING 1 MG/L 6-PPD 32

FIGURE S15: A CHANGE OF THE TOTAL PEAK AREA (TPA) CONSISTING OF 6-PPDQ AND ITS TPS OVER 96 H OF EXPOSURE. 33

List of tables

TABLE S1: CHEMICALS AND SOLVENTS. 5

TABLE S2: COMPOSITION OF ISO WATER. 5

TABLE S3: SUMMARY OF DEVICES USED IN THIS STUDY WITH MODEL AND SUPPLIER INFORMATION. 6

TABLE S4: SOFTWARE WITH INFORMATION ABOUT SUPPLIER AND VERSION. 6

TABLE S5: SUMMARY OF PHYSICOCHEMICAL PROPERTIES OF STUDY COMPOUNDS INCLUDING BASELINE TOXICITY AND LITERATURE DATA OF THE ZFE. 7

TABLE S6: INSTRUMENTAL PARAMETERS OF UPLC-QTOF-MS METHOD FOR TRANSFORMATION PRODUCT ANALYSIS. 13

TABLE S7: COMPOUND-SPECIFIC PARAMETER FOR MRM TRANSITIONS OF STUDY COMPOUNDS INCLUDING RETENTION TIMES. 14

TABLE S8: VALIDATION DATA OF CALIBRATION CURVE OF 6-PPD QUINONE. 15

TABLE S9: VALIDATION DATA OF CALIBRATION CURVE OF 6-PPD. 16

TABLE S10: VALIDATION DATA OF CALIBRATION CURVE OF 4-HDPA. 17

TABLE S11: VALIDATION DATA OF THE ANALYTICAL METHOD USED IN THIS STUDY. 17

TABLE S12: PARAMETERS USED FOR DATA EVALUATION IN UNIFI. 20

TABLE S13: PARAMETER OF MARKERLYNX METHOD USED FOR DETECTION OF TRANSFORMATION PRODUCTS. 20

TABLE S14: OBTAINED INTERNAL CONCENTRATIONS OF 10 µG/L 6-PPDQ AFTER 24 HOURS OF EXPOSURE 24

TABLE S15: REMAINING CONCENTRATIONS OF 6-PPD AND 6-PPDQ AFTER 30S EXPOSURE OF 1 MG/L OF 6-PPD 24

TABLE S16: MEASURED CONCENTRATIONS OF 6-PPDQ IN THE EXPOSURE MEDIUM AT A NOMINAL CONCENTRATION OF 5.0 µG/L. 25

TABLE S17: MEASURED CONCENTRATIONS OF 6-PPDQ IN THE EXPOSURE MEDIUM AT A NOMINAL CONCENTRATION OF 9.75 µG/L. 26

TABLE S18: MEASURED CONCENTRATIONS OF 6-PPDQ IN THE EXPOSURE MEDIUM AT A NOMINAL CONCENTRATION OF 37.5 µG/L. 26

TABLE S19: MEASURED CONCENTRATIONS OF 6-PPD IN THE EXPOSURE MEDIUM AT A NOMINAL CONCENTRATION OF 4.68 µG/L. 27

TABLE S20: MEASURED CONCENTRATIONS OF 6-PPD IN THE EXPOSURE MEDIUM AT A NOMINAL CONCENTRATION OF 18.75 µG/L. 28

1. Chemicals, solvents and solutions

Table S1: Chemicals and solvents.

Substance	CAS number	Supplier	Quality
6-PPD	793-24-8	abcr GmbH (Karlsruhe, Germany)	98 %
6-PPD quinone	2754428-18-5	HPC Standards GmbH (Borsforf, Germany).	99 %
4-HDPA	122-37-2	Alfa Aesar (Kandel, Germany)	98 %
Methanol	67-56-1	Biosolve (Valkenswaard, Netherlands).	> 99 %
Formic acid	64-18-6	Biosolve (Valkenswaard, Netherlands).	> 98 %

1.1 Preparation of ISO water with HEPES buffer

A solution of 10 mM (4-(2-hydroxyethyl)-1-piperazineethanesulfonic acid) (HEPES) with a pH of 7.4 in ISO water was used for the exposure experiments. The ISO water was prepared from four different salts in accordance with DIN EN ISO 7346-3 (1997) [77].

Table S2: Composition of ISO water.

Stock solution	Ingredients	Concentration [g/L]
1	$CaCl_2 \cdot 2\ H_2O$	11.760
2	$MgSO_4 \cdot 7\ H_2O$	4.932
3	$NaHCO_3$	2.52
4	KCl	0.22

1.2 Preparation of calibration standards

Calibration standards were prepared from a 50 mg/L stock solution in acetonitrile. This solution was diluted with methanol to obtain 10 analytical standards for external calibration. The quantitation of analytes was conducted via matrix-matched-calibration. For this purpose, 8 ZFE per sample were treated with 200 µL methanolic standard solution and homogenized according to the section of sample preparation. The matrix-matched calibration standards were diluted 1:1 using ultrapure water to acquire the final standard concentrations between 0.1 and 20 ng/mL.

2. Devices and Software

Table S3: Summary of devices used in this study with model and supplier information.

Device	Supplier, model
Analytical Balance	Mettler, PM4800
Incubator	Heraeus Vötsch, Bioline VB 1514
Centrifuge	VWR, Galaxy 14 D
Ultrasonic bath	Emerson, Branson 5200
Vortexer	Heidolph REAX 2000
pH-Meter	Inolab /WTW; Knick, pH-Meter 765
Light microscope	Olympus, SZX2-ILLT
MillIQ Water System	Merck, Direct 8

Table S4: Software with information about supplier and version.

Software	Supplier, version
MassLynx	Waters, 4.1
TargetLynx	Waters, 4.1
MarkerLynx	Waters, 4.1
Analyst	AB Sciex, 1.6.2
UNIFI	Waters, 1.8.2.169
MulitQuant	AB Sciex, 3.0.3

3. Physicochemical properties of chemicals

Table S5: Summary of physicochemical properties of study compounds including baseline toxicity and literature data of the ZFE.

Compound name	Chemical formula	Molecular Weight	CAS number	Log K_{ow}	pK_a*	neutral fraction at pH 7.4	Log $K_{lip/w}$**	Log $K_{MP/w}$**	Baseline toxicity[c]	Solubility, experimental	Solubility, predicted	LC_{50} from literature[d]
		[g/mol]							[µmol/L]	mg/L	mg/L	[µmol/L] [µg/L]
6PPD-Quinone	$C_{18}H_{22}N_2O_2$	298.176	2754428-18-5	3.3[a]	4.94	100%	3.3	1.07	91.5	0.038[f]; 0.067[g]	2.4[h]	0.446 132.96 (96 hpf)
6PPD	$C_{18}H_{24}N_2$	268.401	793-24-8	4.6[b]	4.6; 0.28	100%	4.6	4.66	4.3	0.563[g]	1.88[h]	1.65 442.62 (96 hpf)
4-HDPA	$C_{12}H_{11}NO$	185.230	122-37-2	2.82[e]	10.46	0%	3.0	2.10	191.2	1.0[i]		

* ACD pKa/GALAS; K_{ow} – octanol/water partition coefficient

** UFZ LSER database; $K_{MP/w}$ – muscle protein/water partition coefficient; $K_{lip/w}$ – lipid/water partition coefficient

[a] ACD (consensus)

[b] Mean of predicted values from EpiSuite and ACD (consensus)

[c] Klüver et al. *Chemosphere* **2016**, *164*, 164–173.

[d] Varshney et al. *Journal of Hazardous Materials* **2022**, *424*, 127623.

[e] PhysPropNCCT experimental value (obtained from COMPTOX)

[f] Hu et al. *Environ. Sci.: Processes Impacts* **2023**, *25* (5), 901–911.

[g] Hiki et al. *Environ. Sci. Technol. Lett.* **2021**, *8* (9), 779–784.

[h] Klöckner et al. *Water Research* **2020**, *185*, 116262.

[i] OECD TG 105 (Bayer AG, **1997**).

4. Quality assurance and quality control

To investigate the stability of 6-PPD and 6-PPDQ during 24 h and 96 h exposure, respectively, both chemicals were dissolved in 25 mL ISO water (10 mM HEPES, pH 7.4) and incubated in glass vessels for 24 h and 96 h at 28 ± 1 °C with a light/dark rhythm of 14h/10h. No zebrafish embryos were added for this experiment. Aliquots of 200 µL were taken before the start and after the exposure, respectively, and stored at -20°C until chemical analysis.

Start concentration of 6-PPD was 1.23 µg/L (10 µg/L nominal concentration) and 7.91 µg/L for 6-PPDQ (10 ng/L nominal concentration). Already at the beginning of the experiment (without ZFE), a contribution of 9 % of 4-HDPA to the TPA was detected (Figure S3). For 6-PPD, after 24 and 96 h of exposure, 45 % (Fig. S1) and 0.5 % (Fig. S2) , respectively, of the measured concentration at the exposure start were detected. Formation of 4-HDPA in 6-PPD solutions after 24 h indicated an abiotic transformation. For 6-PPDQ, after 24 h only 5 % degradation was observed (Figure S1), while no 6-PPDQ could detected after 96 h (Figure S2).

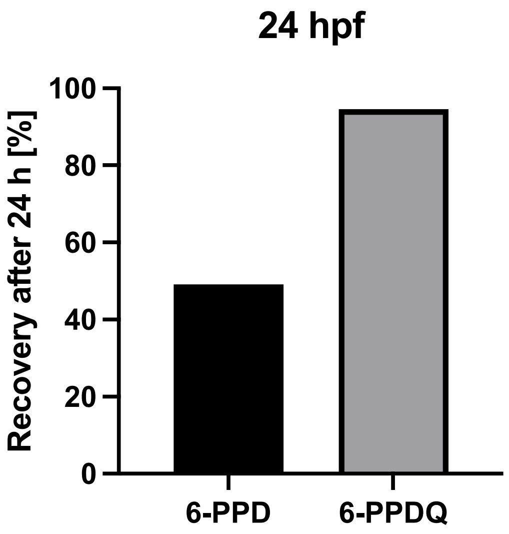

Figure S1: Recovery of 6-PPD and 6-PPDQ after 24 h at 28 ± 1 °C (n =1). Nominal concentration was 10 µg/L. Analytically determined exposure concentrations were 1.23 µg/L and 7.91 µg/L, respectively.

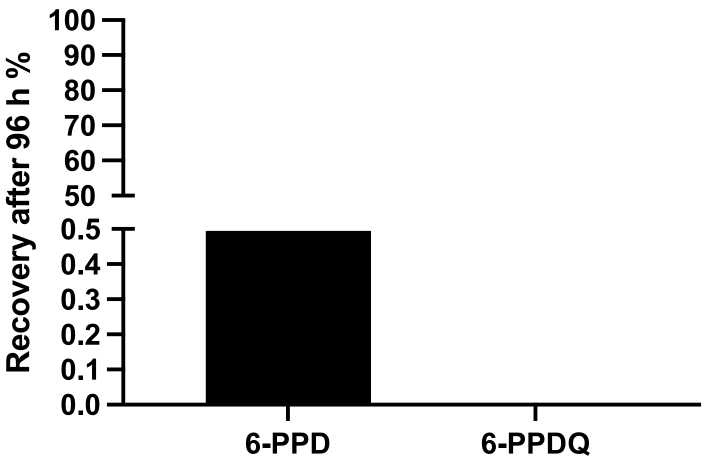

Figure S2: Recovery of 6-PPD and 6-PPDQ after 96 h at 28 ± 1 °C (n = 1). Nominal concentration was 1.2 mg/L of 6-PPD and 0.3 mg/L of 6-PPDQ. Analytically determined start concentrations were 1.0 mg/L and 0.13 mg/L, respectively.

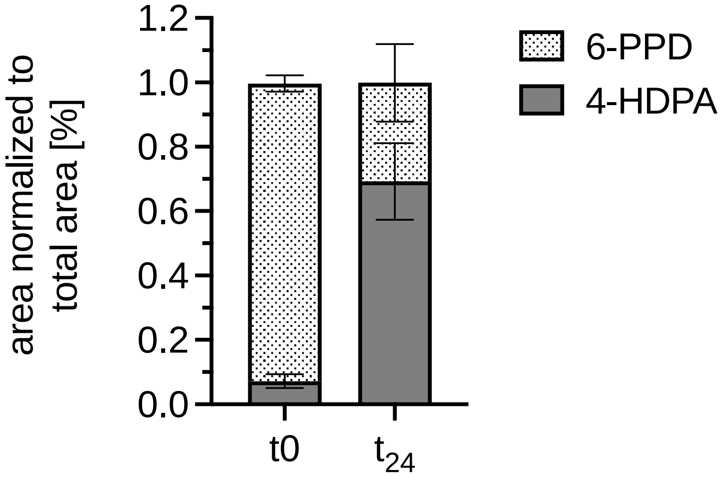

Figure S3: Relative proportion of 4-HDPA and 6-PPDQ in aqueous solution of 6-PPD before (n = 4) and after 24 hours (n = 4) of incubation of 6-PPD. Nominal start concentration of 6-PPD was 18.75 µg/L. Analytically determined start concentration was 6.3 µg/L. Error bars display standard deviations. The concentration of 6-PPDQ detected was < LOQ.

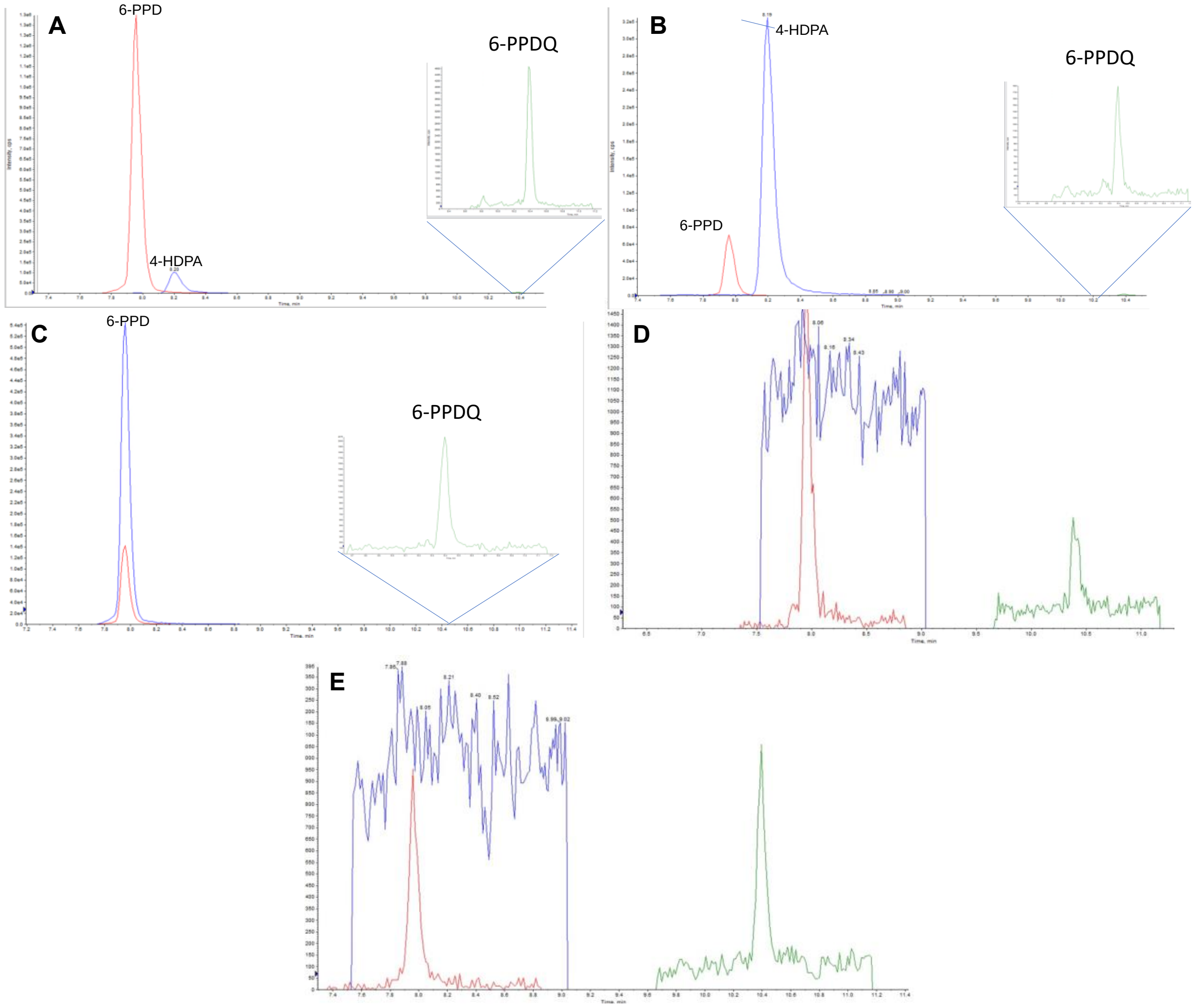

Figure S4: Extracted-ion-chromatograms of 6-PPD, 4-HDPA and 6-PPDQ in exposure media containing 6-PPD after A 0 h (t0) and B 24 h (t24) without ZFE and C extracts of exposed ZFE with 6-PPD. Nominal concentration was 18.75 µg/L and the analytically determined concentration of 6-PPD was 6.3 µg/L. Spiked samples were compared with D blank samples without chemical in ultrapure water and E extracts of non-exposed ZFE after 96 h.

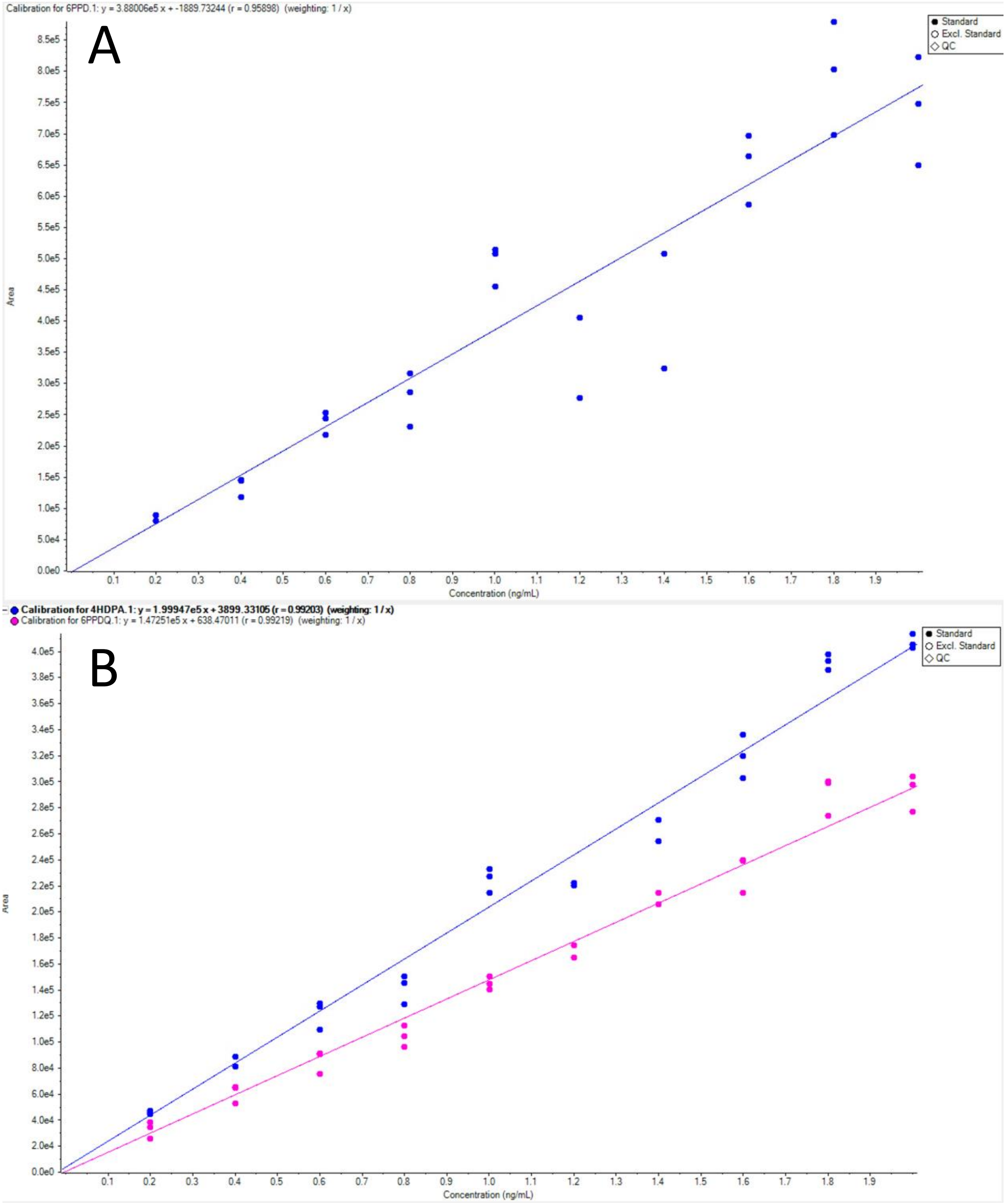

Figure S5: Replicate measurements of standards of **A** 6-PPD and **B** 6-PPDQ, and 4-HDPA in extracts of 8 ZFE per sample. The concentration range was from 0.2 – 2.0 ng/mL. Standards of 6-PPD were prepared separately from its TPs 6-PPDQ and 4-HDPA. Standards were measured three times (n = 3) at intervals of 3 hours.

5. 96 h exposure experiment for identification of biotransformation products

A 96-h exposure experiment was conducted to identify biotransformation products of 6PPD and 6PPD quinone. Exposure concentrations were based on LC_{50} values determined experimentally by Varshney *et al.*.[1] The working solutions were prepared in ISO water containing 10 mM HEPES at pH 7.4 and 0.1 % MeOH. Exposure concentrations were 163 µg/L for 6-PPD and 120 µg/L for 6-PPDQ. Samples of the chemical solutions were taken before and after exposure and stored at -20 °C until chemical analysis.

Fertilized eggs were exposed in 50 mL glass dishes covered with watch glasses. The start of the exposure experiment was approximately 4 ± 1 hpf. Twenty-five ZFE were exposed in crystallization dishes (7.5 cm diameter), with a proportion of one embryo/mL. For quality assurance fertilized ZFE in ISO water (10 mM HEPES, pH 7.4) was used as a negative control. To check the stability of both compounds, exposure media without ZFE were incubated in parallel. All samples were placed in an incubator (28 ± 1 °C) with a light/dark cycle of 14h/10h. Samples were collected after 96 h of exposure with changing the exposure medium every 24 h. ZFE extracts were prepared according to section 2.3 in the manuscript.

5.1 UPLC-QTOF-MS method for identification of transformation products

MeOH extracts from 16 zebrafish embryos exposed to 6-PPD and 6-PPDQ were diluted 1:1 (v/v) with MilliQ water and analyzed via ultra-performance liquid chromatography coupled to time-of-flight mass spectrometry (UPLC-QTOF-MS). The system is equipped with an ACQUITYUPLC system with an HSS T3 column (100 × 2.1 mm, 1.7 µm) coupled to a XEVO XS Q-TOFMS (Waters GmbH, Eschborn, Germany). The instrumental parameters are given in the following table:

Table S6: Instrumental parameters of UPLC-qTOF-MS method for transformation product analysis.

Parameter	Value
Flow rate	0.45 µL/min
Column temperature	45 °C
Capillary voltage	0.7 kV (positive mode)
	- 1.3 kV (negative mode)
Source temperature	140 °C
Desolvation temperature	550 °C
Sampling cone voltage	20 V
Source offset	50 V
Cone gas	nitrogen
Collision gas	argon
Desolvation gas flow	950 L/h
Scan time	0.15 s
Collision energy	4 eV (molecular ions); 15 - 35 eV (fragments)
Mobile phase	A: MilliQ water + 0.1 % formic acid
	B: methanol + 0.1 % formic acid
Solvent gradient	0 min 2% B, 12.25 min 99% B, 15.00 min 99% B; 15.10 min 2% B, 17.00 min 2% B.

6. HPLC-MS/MS analysis

Quantitative analysis was performed using a 1290 Infinity HPLC system (Agilent Technologies, Böblingen, Germany) coupled to a Qtrap 5500 triple-quadrupole mass spectrometer (AB Sciex, Darmstadt, Germany) with a TurboIonSpray interface. The measurements were controlled by Analyst (version 1.5.2, AB Sciex). The LC system was equipped with a degasser, a binary pump with high-pressure gradient mixer, a thermostatic autosampler (temperature set to 4 °C) and a column oven (set to 30 °C). The injection volume was 10 µL. For chromatographic separation, an Atlantis T3 C_{18}-phase column (2.1 mm x 50 mm, 3 µm; Waters, Eschborn,

Germany) with an Atlantis T3 Security Guard column (2.1 x 10 mm, Waters, Eschborn, Germany) was used. The mobile phases were MilliQ water with 0.1% formic acid (eluent A) and methanol with 0.1% formic acid (eluent B). Separation of analytes was performed with 0.3 mL/min flow rate using the following gradient program: 0.0 min, 1 % eluent B; 0.8 min, 1 % eluent B; 10 min, 95 % eluent B; 15 min, 95 % eluent B; 15.1 min, 1 % eluent B; 20 min, 1 % eluent B.

The detection of the analytes was conducted via multiple-reaction-monitoring (MRM). For this purpose, the scheduled MRM mode was used. The analysis of the molecular ions was performed in 2400 cycles with a cycling time of 0.5 seconds. The MRM detection window was set to 90 sec and the target scan time to 0.5 sec. All analytes were detected in the positive ionization mode. The source parameters were as follows: gas 1 at 50 psi, gas 2 at 50 psi and curtain gas at 40 psi. The temperature of the ion source was set to 550 °C and the ionization voltage was 5500 V. The entrance potential was 10 V. Compound-specific MRM parameters are given in the following table.

Table S7: Compound-specific parameter for MRM transitions of study compounds including retention times.

Analyte	ESI polarity	Precursor ion	EP	DP	Product ions	CE	CXP	Retention time
		[m/z]	[V]	[V]	[m/z]	[V]	[V]	[min]
6-PPD	positive	298.979	10.0	81.0	241.10	41.0	20.0	10.41
Quinone			10.0	81.0	215.10	25.0	20.0	10.41
6-PPD	positive	269.079	10.0	96.0	106.90	61.0	10.0	8.09
			10.0	96.0	184.0	35.0	16.0	8.09
4-HPDA	positive	186.095	10.0	66.0	80.00	59.0	12.0	8.28
			10.0	66.0	109.00	33.0	10.0	8.28

6.1 Matrix-matched-calibration

a) 6-PPD quinone

Table S8: Validation data of calibration curve of 6-PPD quinone.

Expected Concentration [ng/mL]	Mean Calculated Concentration [ng/mL]	Accuracy %	R^2
0.1	0.11480	114.8	0.99806
0.25	0.23792	95.2	
0.5	0.47727	95.5	
0.75	0.69432	92.6	
1.0	N/A	N/A	
2.5	2.40737	96.3	
5.0	5.23283	104.7	
7.5	8.00990	106.8	
10.0	9.42559	94.3	
20.0	N/A	N/A	

b) 6-PPD

Table S9: Validation data of calibration curve of 6-PPD.

Expected Concentratio n	Mean Calculated Concentration	Accuracy	R^2
[ng/mL]	[ng/mL]	%	
0.1	0.11326	113.3	0.99582
0.25	0.23031	92.1	
0.5	0.36983	74.0	
0.75	0.62594	83.5	
1.0	0.89545	89.6	
2.5	2.45528	98.2	
5.0	4.89017	97.8	
7.5	6.59380	87.9	
10.0	N/A	N/A	
20.0	21.39760	92.1	

c) 4-HDPA

Table S10: Validation data of calibration curve of 4-HDPA.

Expected Concentration	Mean Calculated Concentration	Accuracy	R^2
[ng/mL]	[ng/mL]	%	
0.1	0.10086	134.6	0.99326
0.25	N/A	N/A	
0.5	0.64082	N/A	
0.75	0.42359	128.2	
1.0	0.85269	56.5	
2.5	2.35779	85.3	
5.0	4.62351	94.3	
7.5	7.36569	92.5	
10.0	10.96778	98.2	
20.0	N/A	N/A	

6.2 Method validation

A methanolic standard mix with a concentration of 1 mg/mL was also used to determine the limit of detection (LOD) and limit of quantification (LOQ). Ten calibration solutions ranging from 0.05 to 10 ng/mL were prepared in ZFE matrix (8 ZFE per concentration) from this standard and measured three times via HPLC-MS/MS. The obtained LOD, LOQ and linear range data are summarized in the following table.

Table S11: Validation data of the analytical method used in this study.

Analyte	LOD	LOQ	Linear range	R^2
	[ng/mL]	[ng/mL]	[ng/mL]	
6-PPD Quinone	0.089	0.439	0.25 – 10.0	0.99399
6-PPD	0.130	0.638	0.05 – 7.5	0.99712
4-HPDA	0.099	0.488	0.05 – 7.5	0.99423

a) Sample preparation for evaluation of matrix effects

For the determination of matrix effects, 72 ZFE (three replicates consisting of 8 ZFE for three standard solutions in different concentrations) at 24 hpf and 96 hpfwere used. Therefore, 8 ZFE per sample were collected in FastPrep tubes and extracted according to the section above. For every sample, 150 µL of the supernatant was used and diluted with 150 µL of an aqueous standard mixture containing all analytes. The resulting concentrations for the evaluation of matrix effects were 0, 0.5, 1 and 5 ng/mL to cover the calibration range.

b) Sample preparation for determination of method recovery

For the determination of method recovery, the same approach was used as for the evaluation of matrix effects for ZFEs at 96 hpf. The only difference was that methanolic standard solutions were used for extraction, which was subsequently diluted with the same amount of water.

6.3 Results matrix effects and method recovery

Method recoveries in ZFE matrix (96 hpf) ranged between 85 % and 95 % for 6-PPDQ, 107 % - 123 % for 6-PPD and 83 % - 107 % for 4-HDPA. For 6-PPDQ, no matrix effects at the developmental stages (24 and 96 hpf) tested were observed (matrix recoveries around 100 % for all test concentrations). Slightly stronger differences were observed for 6-PPD and 4-HDPA. The analytical response of 6-PPD in matrix with 24 hpf old ZFE showed by means 20 % higher signal than at 96 hpf. The stronger deviations for 6-PPD can be explained by stronger tendency to sorption as well as limited chemical stability (hydrolysis in aqueous solution). Little life-stage specific differences in matrix effects by means 6 % for 4-HDPA were observed. The data obtained for clearly indicate that the method developed is well suited for determination of internal concentrations in ZFE matrix at various developmental stages using matrix-matched calibration approach. Since, quantification of 6-PPD is challenging at all, the observed deviations in matrix effects are still acceptable and the data can be used to assess its uptake behavior into the ZFE.

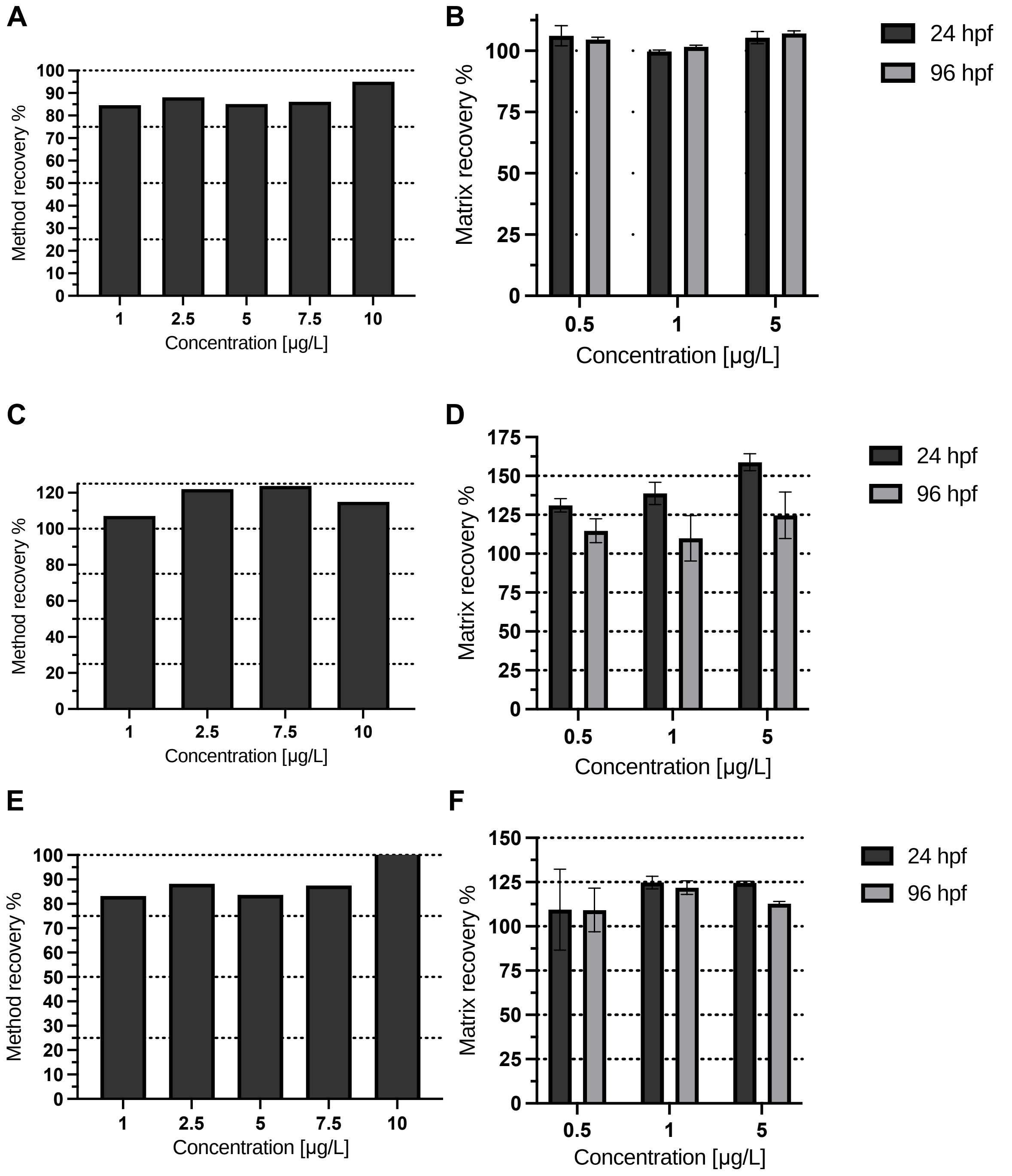

Figure S6: Method recoveries (8 ZFE at 96 hpf) and matrix effects (8 ZFE at 24 hpf and 96 hpf) for 6-PPDQ (**A,B**), 6-PPD (**C,D**) and 4-HDPA (**E,F**). Method recovery was tested for 4 different concentrations (1 – 10 µg/L). Matrix effect assessment was conducted for 3 different concentrations (0.5, 1.0 and 5.0 µg/L) in triplicates (n = 3). Method recovery was assessed using one sample per concentration (n = 1).

7. Software parameters for identification of transformation products

Parameters for TPs analysis using UNIFI and MarkerLynx

Table S12: Parameters used for data evaluation in UNIFI.

Parameter	Value
3D-Peak picking	0 – 17 min
Match tolerance	5.0 ppm
Fragment match tolerance	0.1 mDa
Maximum allowed score	4
Lockspray mass: leucin-enkephaline	556.2771 (ESI positive)
	554.2620 (ESI negative)
Transformations	Phase I (max. 1)
	Phase II (max. 2)
Dealkylation tool	aktiv
Adducts	+H, +K, +Na, $+NH_4$ (ESI positive)

***Table S13**: Parameter of MarkerLynx method used for detection of transformation products.*

Property	Value
Function	1
Analysis type	Peak Detection
Initial retention time	1.0
Final retention time	10.0
Low mass	50.0
High mass	1200.0
XIC window (Da)	0.01
Use relative retention time	No
Apply smooting	No
Noise elimination level	0.0
Deisotope data	Yes
Replicate % Minimum	0.0

8. The influence of adsorption on internal concentrations

Due to the hydrophobicity of 6-PPD and 6-PPDQ, it was necessary to determine to what extent adsorption to the chorion or on the surface of the ZFE after hatching (48 – 96 hpf) affected the measured internal concentration. Furthermore, rapid diffusion into the perivitelline space (PVS) (for 2 - 24 hpf) could bias the assessment of embryonic concentrations in early stages, when it is not possible to manually remove the chorion.

For assessment of adsorption, the residual amount of 6-PPD and 6-PPDQ detectable after a 30-second-exposure of ZFE at 72 hpf to a 1 mg/L exposure solution each was investigated. The proportion of this residual concentration was related to the measured internal concentration after long-term exposure. Furthermore, we tested the influence of adsorption to the chorion and distribution in the PVS on the measured internal concentration by extracting ZFE after 24 hoe ZFE with chorion and comparing them with ZFE that were previously dechorionated.

8.1 24-h-exposure with and without dechorionation before extraction

To estimate the extent to which adsorption of 6-PPD and 6-PPDQ to the chorion during the early stages of life affects the measured internal concentration, a 24-h exposure was performed. For this purpose, 20 ZFE were exposed to 20 mL of 10 µg/L exposure solution containing either 6-PPD or 6-PPDQ for 24 h at 28 ± 1 °C. The analytically determined exposure concentrations were 1.23 µg/L and 7.91 µg/L, respectively. The exposure was started at 4 ± 1 hpf. For negative controls, 20 ZFE were exposed in ISO water without chemical and, additionally, chemical solutions were incubated without ZFE under the same conditions. Samples of the exposure medium were collected at the beginning and the end of the experiment to test the stability of 6-PPD and 6-PPDQ. After exposure, 8 ZFE per replicate (16 ZFE in total for each compound) were dechorionated and extracted according to section 2.3 in the manuscript. Additionally, ZFE were processed without dechorionation before extraction. Samples were stored at -20 °C until chemical analysis. To relate the obtained concentrations

to data using different exposure solutions, relative internal concentrations were calculated by division of internal by external concentration.

Adsorption to the chorion or occurrence in the PVS may lead to overestimation of internal concentrations in earlier life stages of ZFE. The concentrations determined in extracts of ZFE exposed to 6-PPD and 6-PPDQ for 24 h (28 hpf) processes with chorion and after dechorionaton were compared. If processed with the chorion the resulting relative total concentration was much higher compared to the concentration determined after dechorionation. For 6-PPD the factor was 4, for 6-PPDQ even 6 (**Figure S7**). Thus, the adsorption of both compounds to the chorion was so strong that dechorionation is mandatory before homogenization and extraction. This confirms earlier results[2], in which the influence of PVS on the quantification of internal concentrations of four different substances in ZFE was investigated. As dechorionation was not possible for very early developmental stages (2 – 8 hpf), data from these stages were excluded for the uptake study.

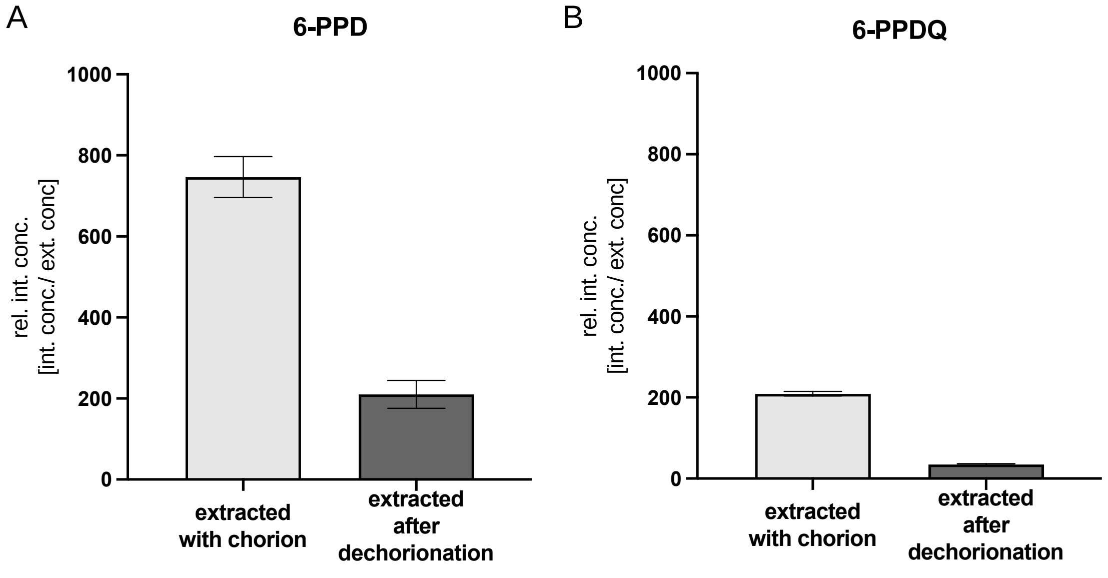

Figure S7: Assessment of the adsorption to the chorion of 6-PPD and 6-PPDQ to exposed ZFE. Relative internal concentration of 6-PPD **(A)** and 6-PPDQ **(B)** in extracts of exposed ZFE after 24 hpf. Internal concentrations of 6-PPD in ZFE with and without chorion were compared. Nominal exposure concentration was 10 µg/L. Analytically determined external concentrations of 6-PPD and 6-PPDQ were 1.23 µg/L and 7.91 µg/L, respectively. The experiment was conducted in duplicates (n = 2). Error bars display standard deviations. Adsorption experiments were conducted in duplicates. Experiments were started at 4 ± 1 hpf.

8.2 Short-time exposure to study adsorption of 6-PPD and 6-PPDQ to ZFE without chorion at 72 hpf

In order to investigate the bias of adsorption to internal concentration analysis, a short-time exposure (less than 30 s) of 6-PPD and 6-PPDQ in 72 hpf ZFE with 1 mg/L 6-PPD and 6-PPDQ was performed. For this purpose, 8 ZFE per replicate were exposed to 1 mg/L 6-PPD or- 6-PPDQ for 30 seconds at the 72-hpf stage. After 30 seconds, the exposure medium was removed and the ZFE were washed 3x with ISO water. The samples were extracted in pure MeOH according to section 2.3 in the manuscript. Samples from the exposure media were collected to determine the concentration in the exposure solution. The samples were stored at – 20°C until HPLC-MS/MS analysis.

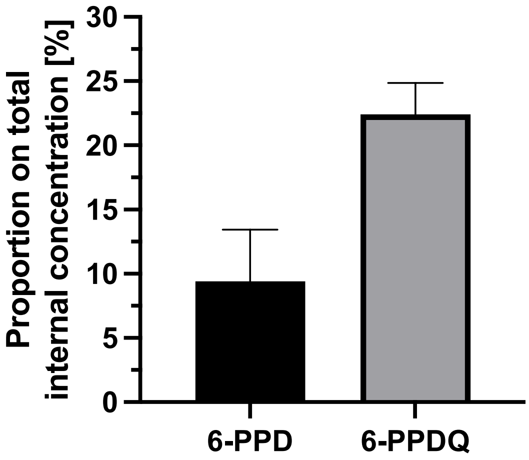

Figure S8: Contribution of adsorption of 6-PPD and 6-PPDQ to internal concentration analysis. To quantify the contribution of adsorption a short-term exposure of 30 s (external concentration 1 mg/L) and washing with ISO water was conducted with 72 hpf embrys and compared to concentration found after 72 h of exposure. The measured exposure concentrations of 6-PPD and 6-PPQ were 173 µg/L and 770 µg/L, respectively. The experiment was conducted in duplicates (n = 2). Error bars display standard deviations.

Table S14: Obtained internal concentrations of 10 µg/L 6-PPDQ after 24 hours of exposure. Extracts with and without previous dechorioniation were compared. The exposure was started at 4 ± hpf. The experiment was performed in duplicates (n = 2).

Compound	Dechorionation	Nominal exposure concentration [µg/L]	Conc. Extract (Replicate 1) [µg/L]	Conc. Extract (Replicate 2) [µg/L]	Mean conc. Extracts [µg/L]	ng/organism	V ZFE at 24 hpf [µL]	Int conc [mg/L]	Mean external concentration [mg/L]	Relative int conc. [int. Conc. / ext. Conc.]
6PPDQ	no	10.00	15.92	16.58	16.25	0.41	0.18	2.24	0.0077	291.45
6PPDQ	yes	10.00	2.58	2.61	2.59	0.06	0.18	0.36		46.53
6-PPD	no	10.00	7.25	6.59	6.92	0.17	0.18	0.96	0.00092	1039.14
6-PPD	yes	10.00	2.18	1.72	1.95	0.05	0.18	0.27		292.81

Table S15: Remaining concentrations of 6-PPD and 6-PPDQ after 30s exposure of 1 mg/L of 6-PPD or 6-PPDQ to ZFE at 72 hpf. The exposure was started at 4 ± hpf. ZFE were washed three times with ISO water before subsequent sample preparation. Relative internal concentrations were calculated by division of internal by external concentration. The experiment was performed in duplicates (n = 2) for 6-PPD and in triplicates (n = 3) for 6-PPDQ.

Compound	Life stage [hpf]	Nominal exposure con. [mg/L]	Measured external conc. mg/L	Extract conc. [ng/mL] Rep. 1	Extract conc. [ng/mL] Rep. 2	Extract conc. [ng/mL] Rep 3	Mean extract conc. [ng/mL]	ng/organism	V ZFE at 72 hpf [µL]	Int. Conc [mg/L]	Relative int. conc. after 30 s exposure	Total rel. int conc after 72 h exposure	Proportion of adsorbed compound on total int.conc.%
6-PPD	72	1.00	0.17	53.46	28.35		40.90	0.67	0.229	4.06	15.30	163.20	9.40
6-PPDQ	72	1.00	0.77	154.16	139.63	123.98	139.26	3.48	0.229	15.20	17.90	79.30	22.40

9. Internal and external concentrations of 6-PPD and 6-PPDQ in zebrafish embryos

9.1 Exposure medium concentrations

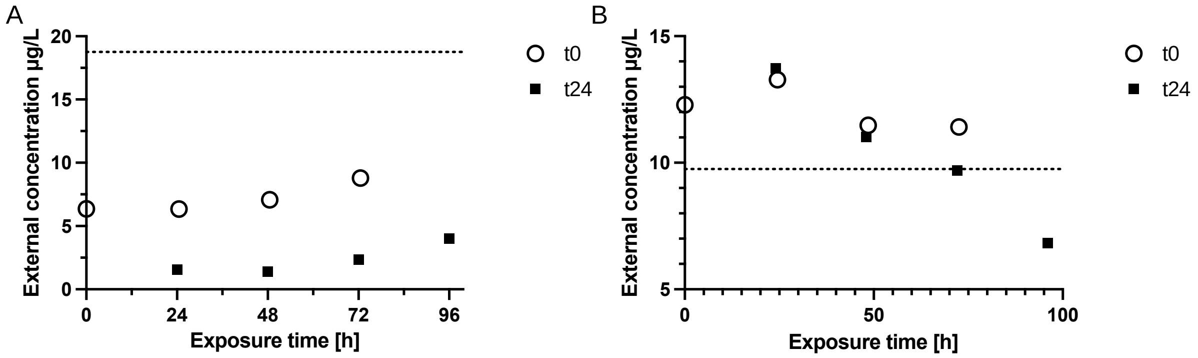

Figure S9: Time course of external concentrations over 96 hours of exposure. **A** External concentrations of 6-PPD at a nominal concentration of 18.75 µg/L. **B** External concentrations of 6-PPDQ at a nominal concentration of 9.75 µg/L. The ratio between exposure medium and embryos was one ZFE per mL. The exposure medium was changed daily. Dotted lines represent nominal exposure concentrations. t0 – concentration directly after changing the exposure medium; t24 – concentration after 24 h. Each data point represents one replicate (n = 1).

Table S16: Measured concentrations of 6-PPDQ in the exposure medium at a nominal concentration of 5.0 µg/L.

time [h]	µg/L	µmol/L
nominal conc.	**5.00**	0.017
0	5.85	0.020
24	4.57	0.015
24.5	5.78	0.019
48	4.73	0.016
48.5	5.15	0.017
72	4.24	0.014
72.5	4.55	0.015
96	3.92	0.013

Table S17: Measured concentrations of 6-PPDQ in the exposure medium at a nominal concentration of 9.75 µg/L.

time [h]	µg/L		µmol/L	
	**replicate 1**	**replicate 2**	**replicate 1**	**replicate 2**
nominal conc.	**9.75**		0.033	
0	12.29	13.16	0.041	0.044
2	13.59		0.046	
4	13.94		0.047	
24	13.73	5.48	0.046	0.018
24.5	13.28	6.83	0.045	0.023
48	11.01	6.67	0.037	0.022
48.5	11.48	7.36	0.038	0.025
72	9.70	2.68	0.033	0.009
72.5	11.41	7.19	0.038	0.024
96	6.82	2.32	0.023	0.008

Table S18: Measured concentrations of 6-PPDQ in the exposure medium at a nominal concentration of 37.5 µg/L.

time [h]	µg/L		µmol/L	
	**replicate 1**	**replicate 2**	**replicate 1**	**replicate 2**
nominal conc.	**37.5**		0.126	
0	25.67	22.61	0.086	0.076
2	17.34		0.058	
4	16.27		0.055	
6	27.79		0.093	

8	18.38		0.062	
24	20.22	21.79	0.068	0.073
24.5	20.26	25.80	0.068	0.087
48	23.70	22.87	0.079	0.077
48.5	22.31	28.79	0.075	0.097
72	23.44	22.48	0.079	0.075
72.5	18.19	30.70	0.061	0.103
96	17.28	14.69	0.058	0.049

Table S19*:* Measured concentrations of 6-PPD in the exposure medium at a nominal concentration of 4.68 µg/L.

time	µg/L	µmol/L
nominal	4.68	0.0174
0	3.44	0.0128
24	0.43	0.0016
24.5	1.73	0.0064
48	0.36	0.0014
48.5	0.93	0.0035
72	0.80	0.0030
72.5	1.92	0.0071
96	0.68	0.0025

Table S20: Measured concentrations of 6-PPD in the exposure medium at a nominal concentration of 18.75 µg/L.

time [h]	µg/L	µmol/L
nominal	18.750	0.070
0	6.354	0.024
24	1.546	0.006
24.5	6.346	0.024
48	1.388	0.005
48.5	7.070	0.026
72	2.343	0.009
72.5	8.799	0.033
96	4.017	0.015

9.2 **Internal concentrations of 6-PPD and 6-PPDQ in the ZFE**

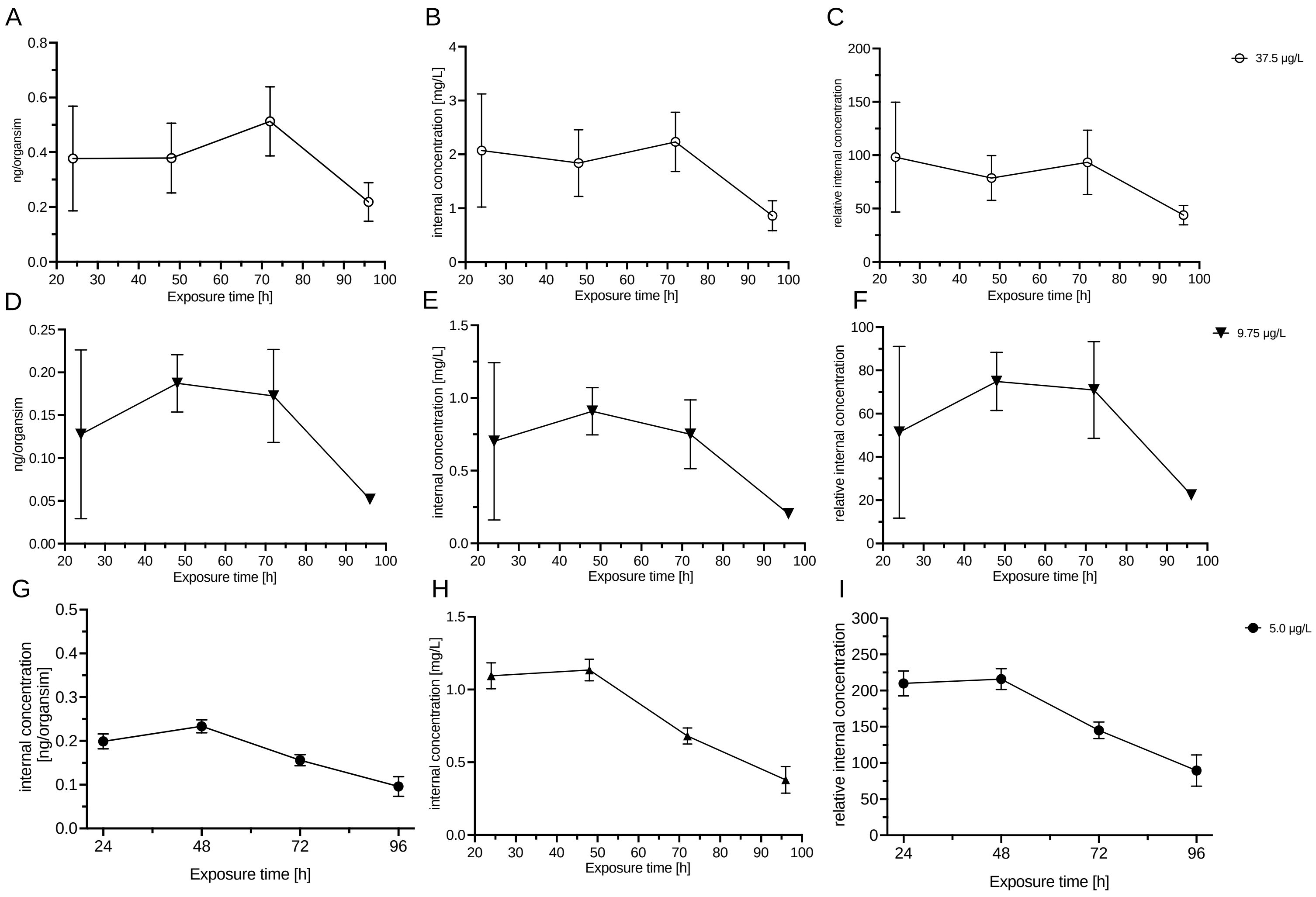

Figure S10: Uptake curves of 6-PPDQ from three independent experiments. Each row represents one experiment. Internal concentrations are shown in ng/organism (A, D, G), mg/L (B,E,H) and in relative internal concentrations (C,F,I). Exposure experiments were performed using 37.5; 9.75 and 5.0 µg/L nominal exposure concentrations. Two technical replicates were obtained for each experiment. Standard deviations are given as error bars. Each exposure experiment was started at 4 ± 1 hpf.

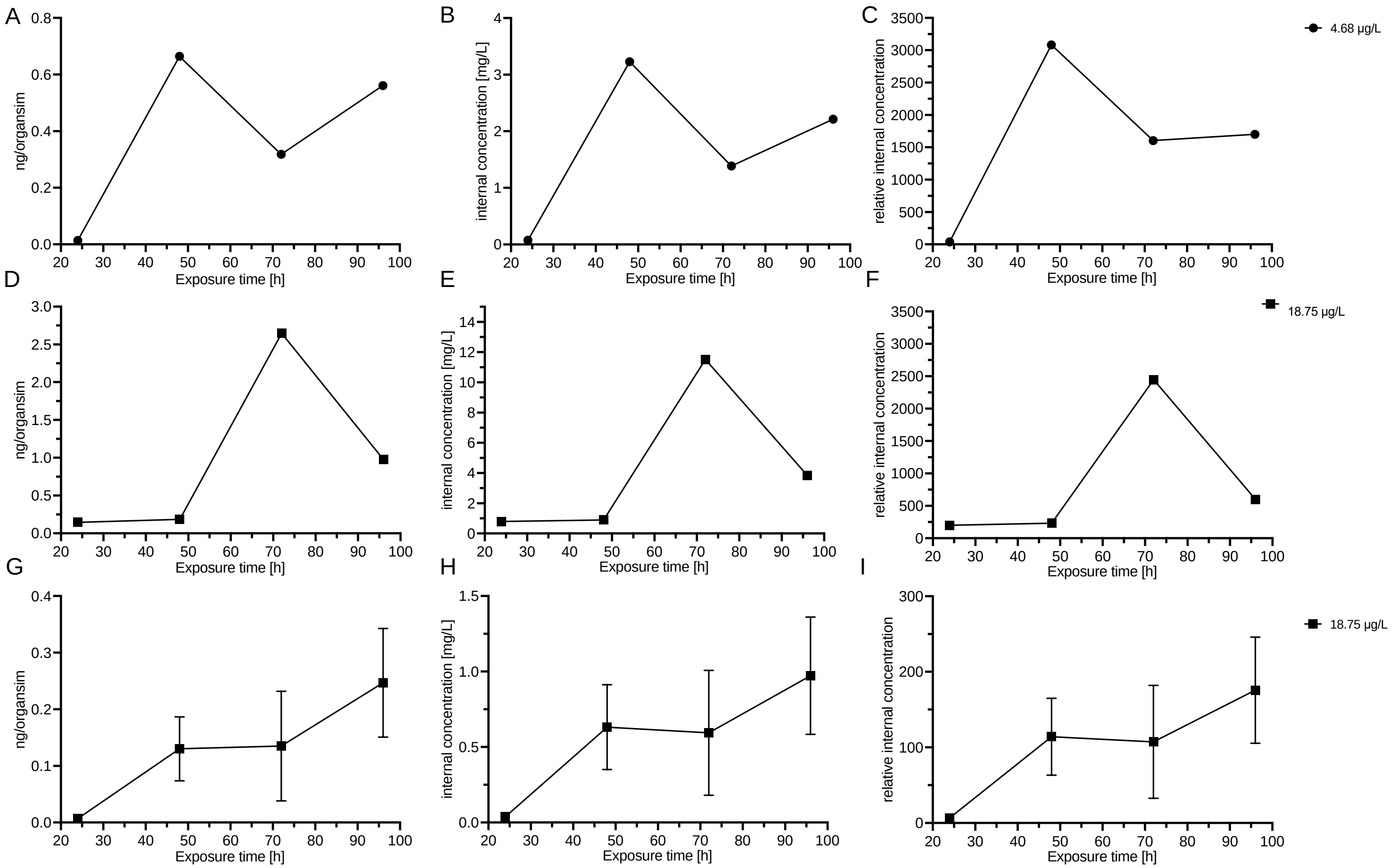

Figure S11: Uptake curves of 6-PPD from three independent experiments. Each row represents one experiment. Internal concentrations are shown in ng/organism (A, D, G), mg/L (B,E,H) and in relative internal concentrations (C,F,I). Exposure experiments were performed using 18.75; and 4.68 µg/L nominal exposure concentrations. For the first and experiment (A-C; D-F), only one data point was recorded per time point and concentration (n =1). For the third experiment, three technical replicates were obtained (n = 3). Standard deviations are given as error bars. Each exposure experiment was started at 4 ± 1 hpf.

10. Calculation of internal concentrations

Internal concentrations were calculated according to the publication of Lisa Bittner and co-workers. [3] Briefly, the obtained concentrations from the diluted samples were multiplied by the corresponding dilution factor. Subsequently, these concentrations were multiplied by the extraction volume of 0.2 mL to obtain the amount of the compound in the sample. This mass was divided by the number of ZFE in the sample to get the amount of the analyte in ng/organism. To convert these values to internal molar concentrations these masses per organism were divided by the volume of the zebrafish embryo and subsequent division by the molecular weight, respectively. Halbach *et al*. established a model to estimate the volume of the embryo at every life stage (**Figure S12**).[4]

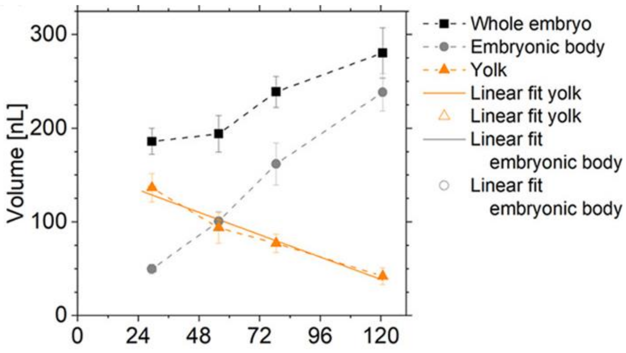

Figure S12: Volumes of whole embryos, embryonic bodies and yolk of zebrafish embryos. Linear regression:

$y = 0.996x + 158$; $R^2 = 0.85$.[4]

11. Chromatograms and additional Figures

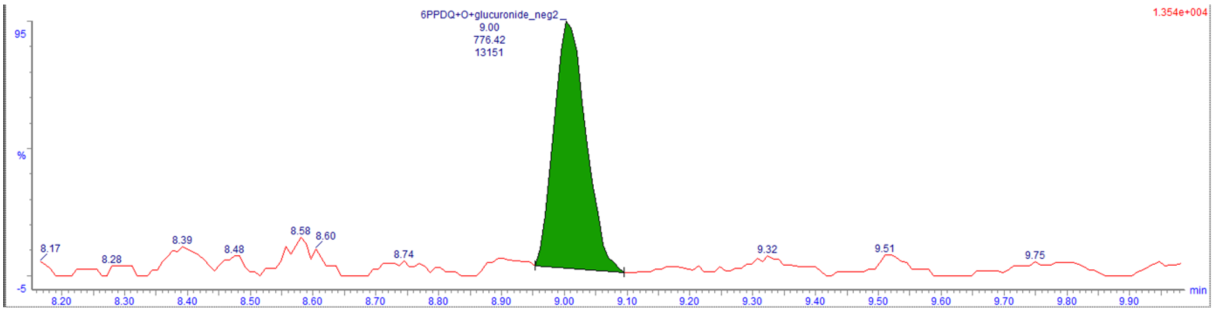

Figure S13: LC-HRMS chromatogram of 6-PPDQ+O+glucuronide in exposure medium containing 37.5 µg/L 6-PPDQ (nominal concentration). The analysis was conducted with ZFE at 96 hpf stage. The transformation product was detected in both positive and negative ionization mode.

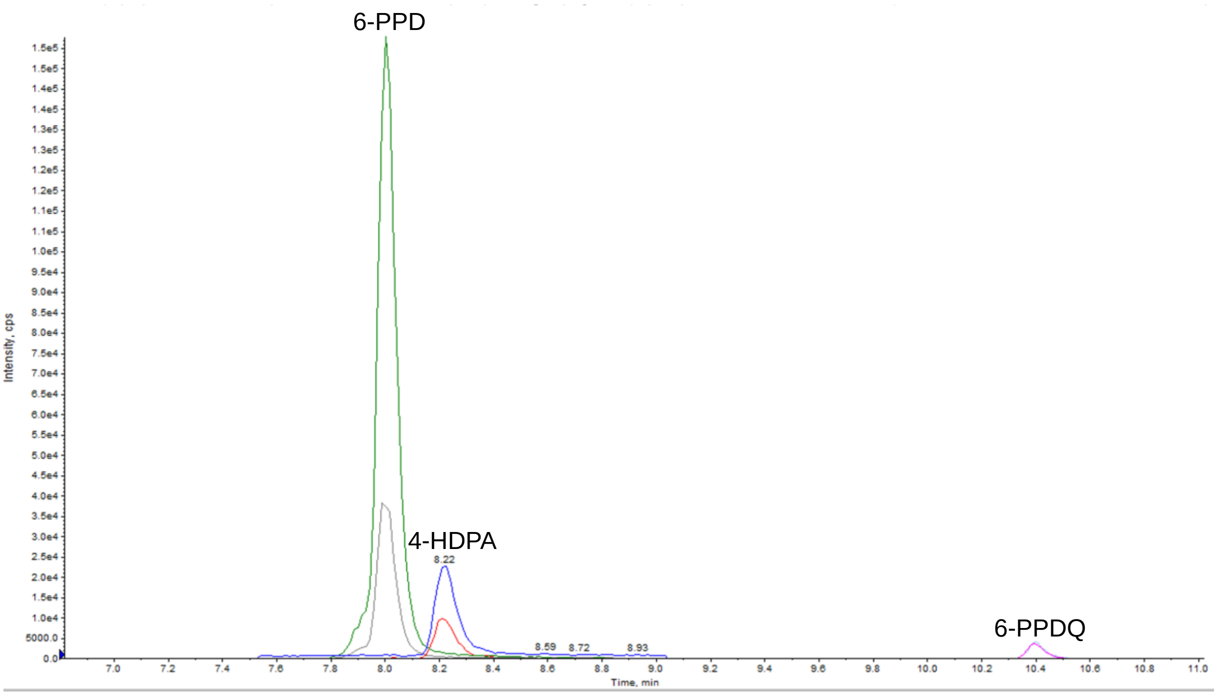

Figure S14: Extracted-ion-chromatogram of 6-PPDQ and 4-HDPA in freshly prepared exposure medium containing 1 mg/L 6-PPD (nominal concentration). Data were recorded in positive ionization-mode.

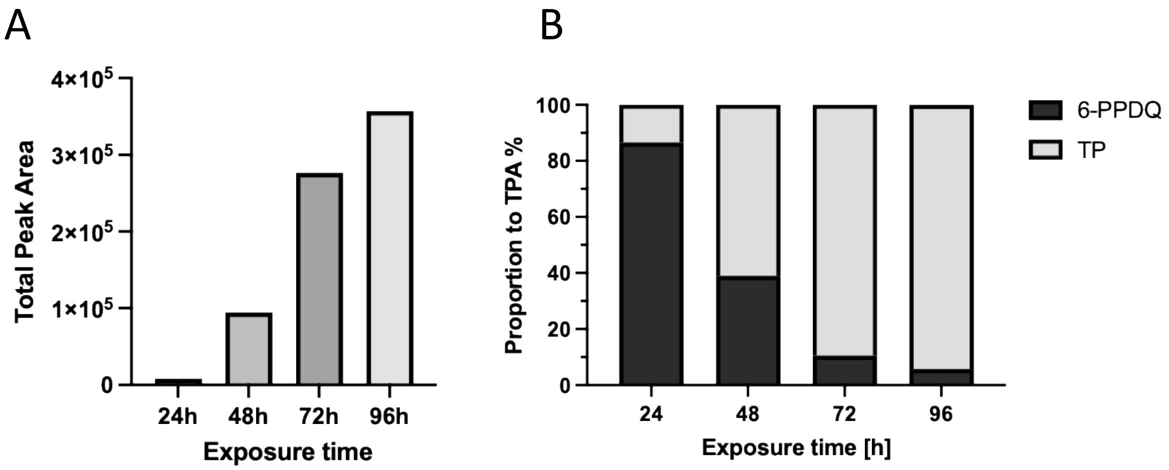

Figure S15: A Change of the total peak area (TPA) consisting of 6-PPDQ and its TPs over 96 h of exposure. **B** Evolution of the proportion of TPs and 6-PPDQ to TPA. The nominal exposure concentration was 9.75 µg/L. The exposure was started at 4 ± 1 hpf.

12. MS spectra of transformation products of 6-PPD and 6-PPDQ

Compound name: 4-HDPA quinonimine + O +SO_3

Chemical formula: $C_{12}H_8NO_5S$

m/z: 278.0131 (-)

Retention time: 4.66 min

Confidence level: 3

Proposed chemical structure:

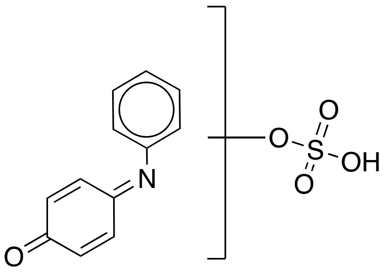

MS/MS spectrum:

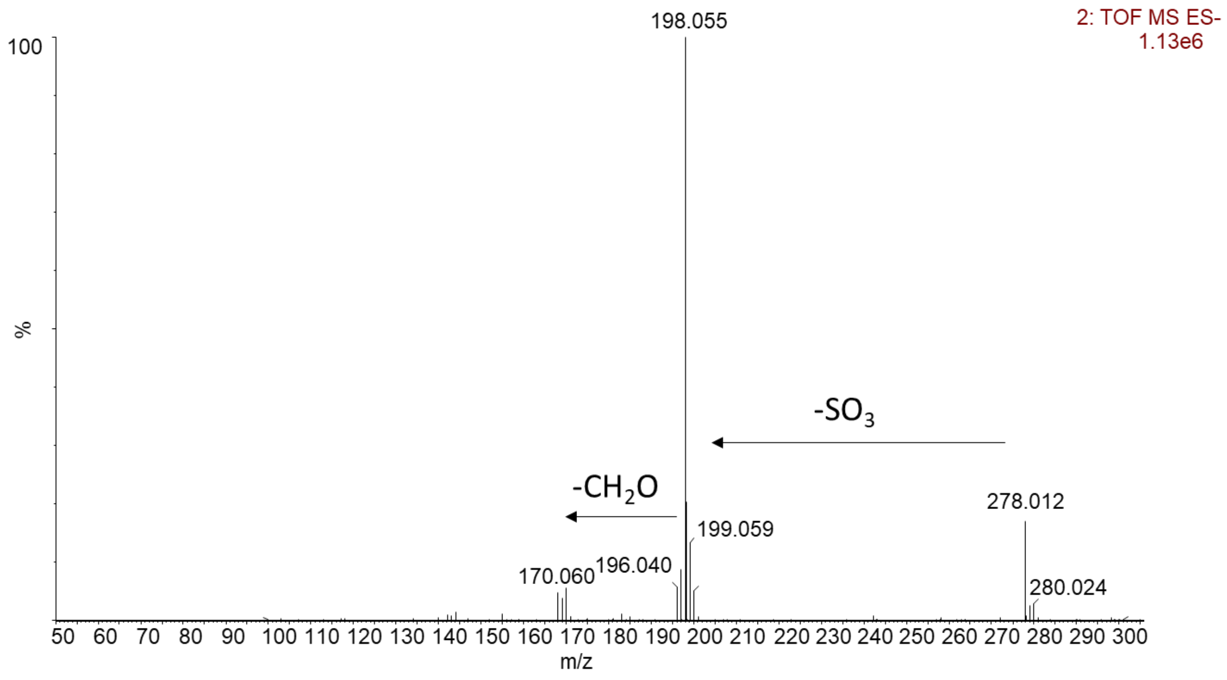

Compound name: 4-HDPA + 2O

Chemical formula: $C_{12}H_{12}NO_3$ (+) / $C_{12}H_{10}NO_3$ (-)

m/z: 218.0817 (+) / 216.0654 (-)

Retention time: 4.56 min

Confidence level: 3

Proposed chemical structure:

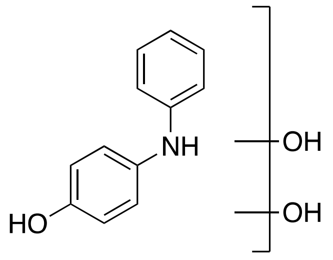

MS/MS spectrum:

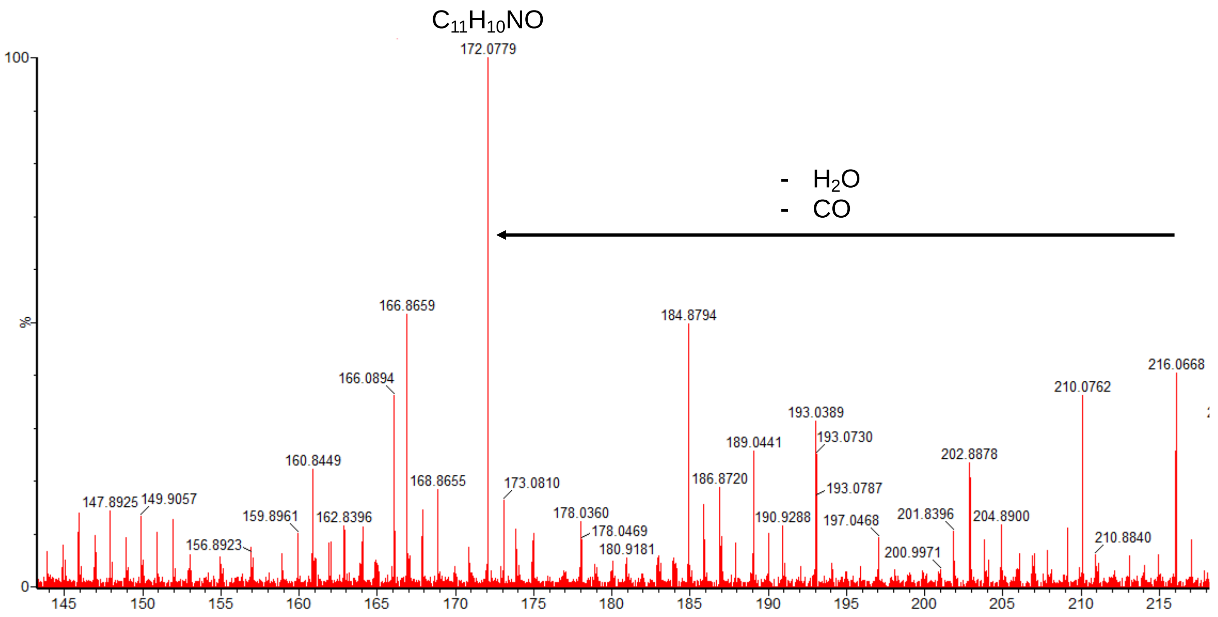

Compound name: 6-PPD+O+glucuronide

Chemical formula: $C_{24}H_{33}N_2O_7$ (+) / $C_{24}H_{31}N_2O_7$ (-)

m/z: 461.2275 (+) / 459.2130 (-)

Retention time: 5.74 min

Confidence level: 3

Proposed chemical structure:

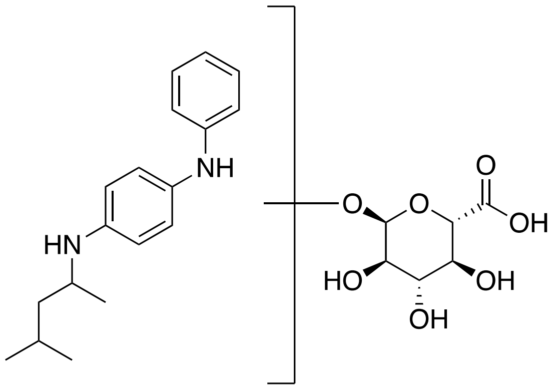

MS/MS spectrum:

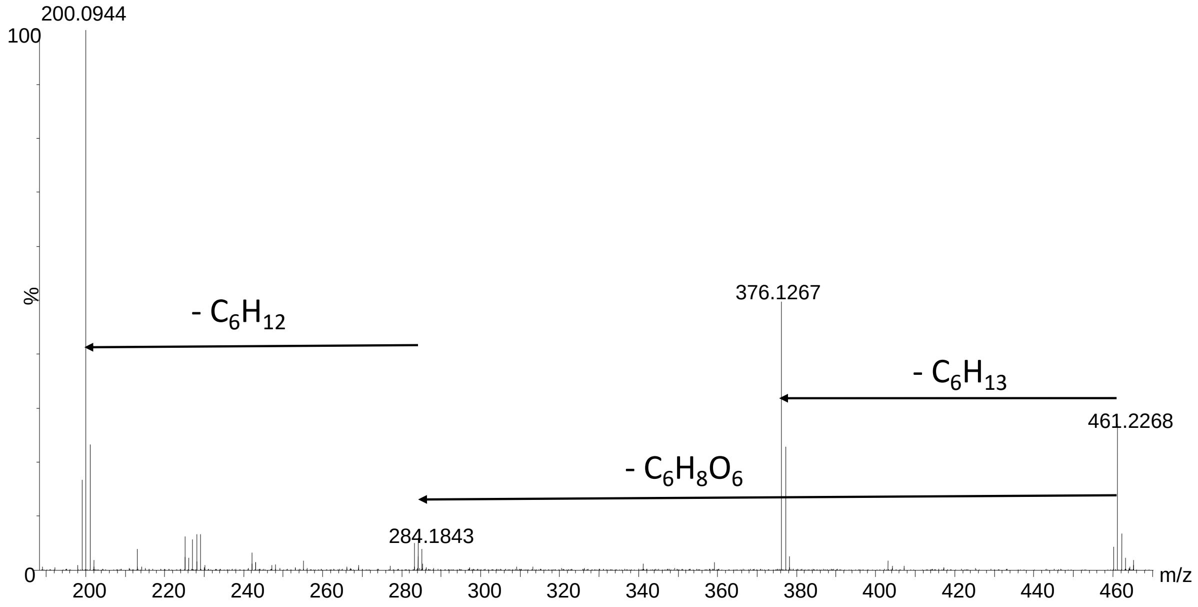

Compound name: 4-HDPA+Cys+glucuronide

Chemical formula: $C_{21}H_{25}N_2O_9S$ (+) / $C_{21}H_{23}N_2O_9S$ (-)

m/z: 481.1276 (+) / 479.1125 (-)

Retention time: 5.91 min

Confidence level: 3

Proposed chemical structure:

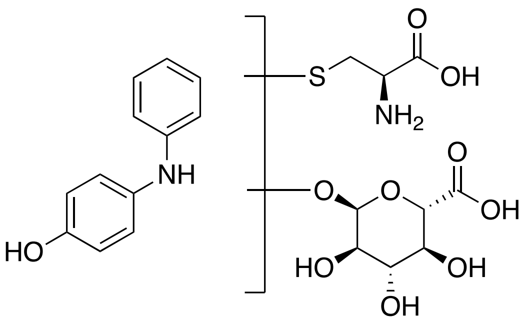

MS/MS spectrum:

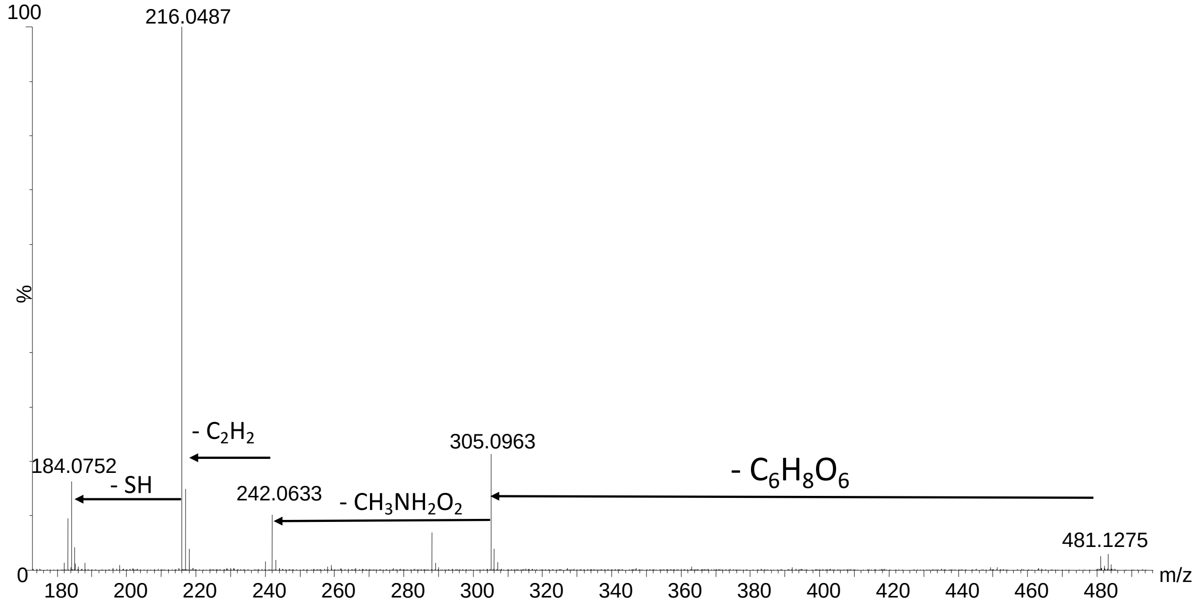

Compound name: 4-HDPA+2O+CH_3

Chemical formula: $C_{13}H_{14}NO_3$ (+)

m/z: 232.0965 (+)

Retention time: 6.07 min

Confidence level: 3

Proposed chemical structure:

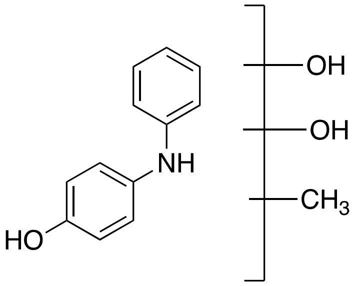

MS/MS spectrum:

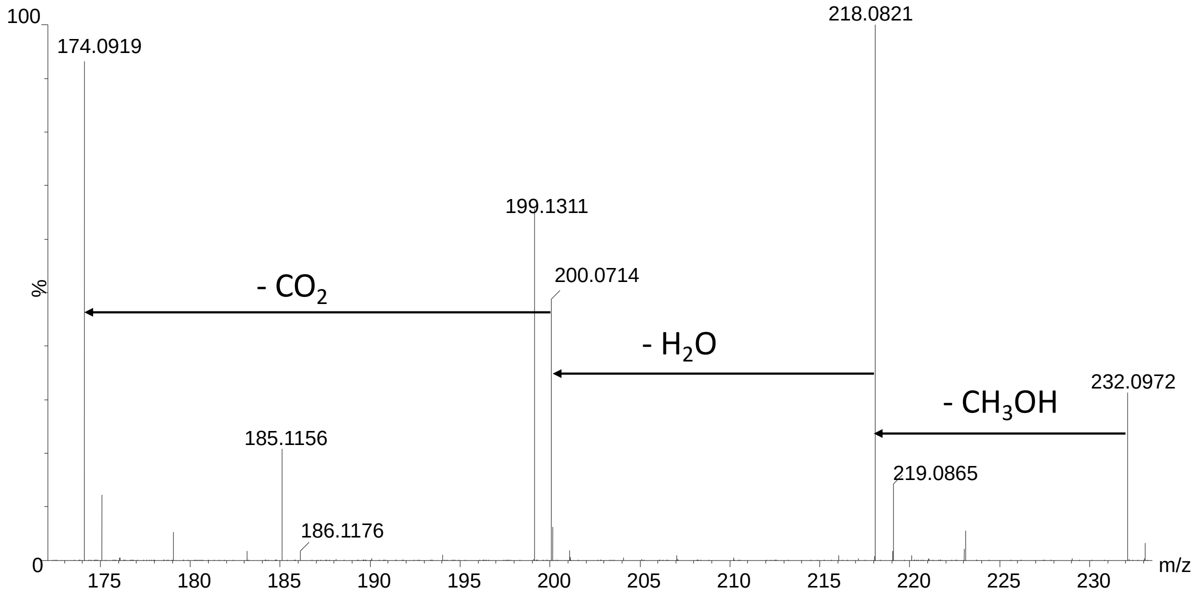

Compound name: 6-PPD+O+glucuronide +glutathione

Chemical formula: $C_{34}H_{46}N_5O_{13}S$ (+) / $C_{34}H_{44}N_5O_{13}S$ (-)

m/z: 764.2814 (+) / 762.2663 (-)

Retention time: 6.26 min

Confidence level: 3

Proposed chemical structure:

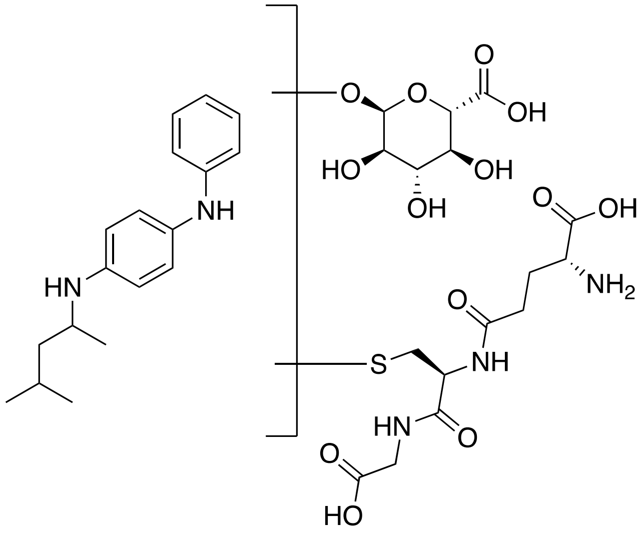

MS/MS spectrum:

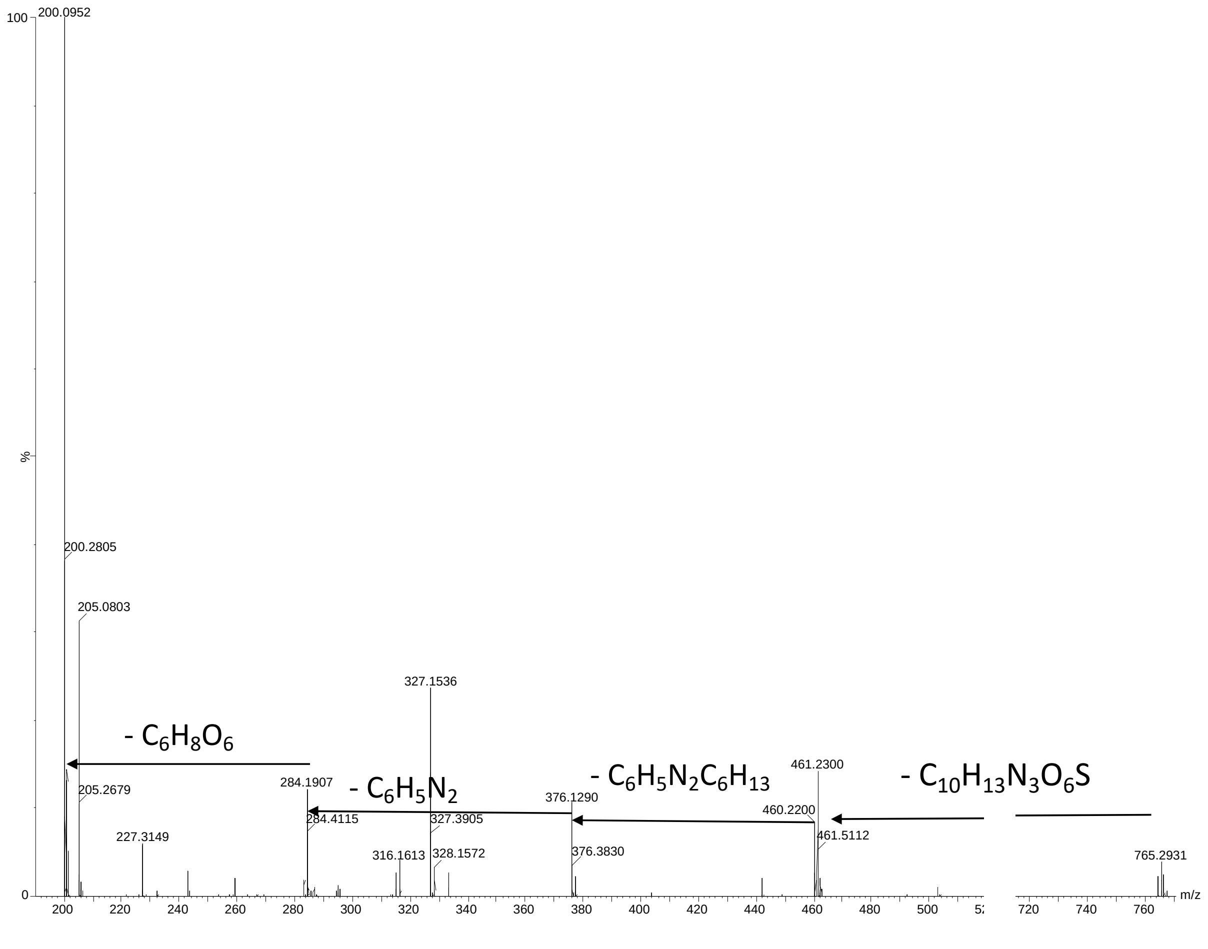

Compound name: 4-HDPA+NAcCys+glutathione

Chemical formula: $C_{27}H_{32}N_5O_{10}S_2$ (+) / $C_{27}H_{30}N_5O_{10}S_2$ (-)

m/z: 650.159 (+) / 648.1440 (-)

Retention time: 6.65 min

Confidence level: 3

Proposed chemical structure:

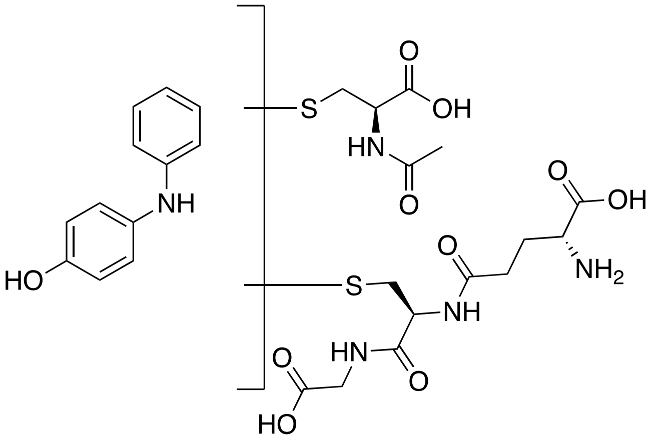

MS/MS spectrum:

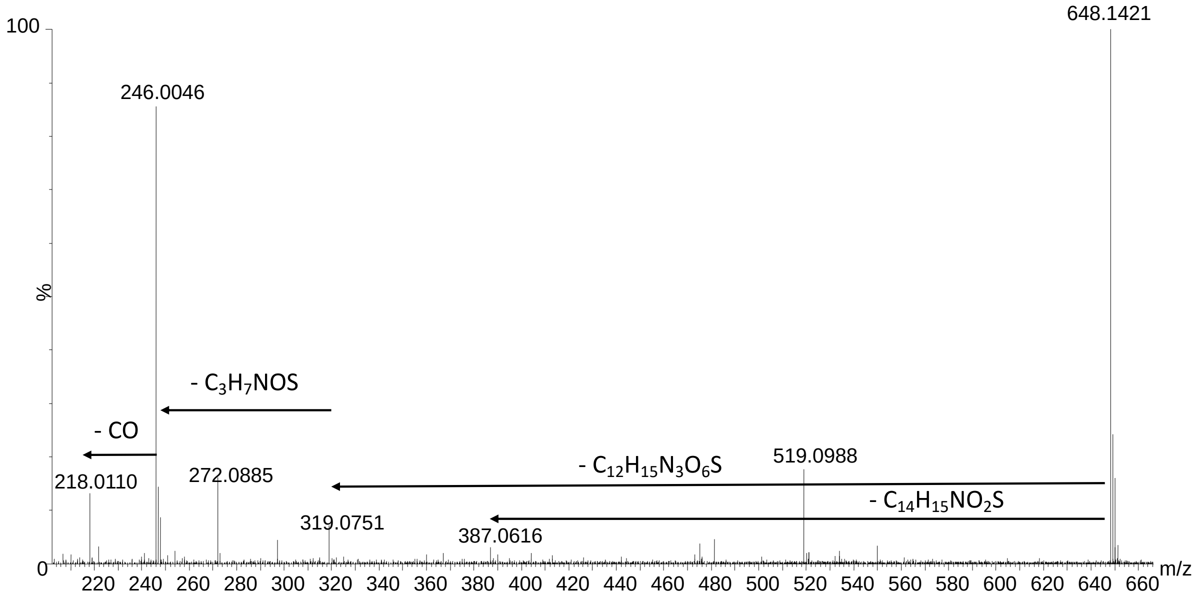

Compound name: 6-PPD+O+Cys

Chemical formula: $C_{21}H_{30}N_3O_3S$ (+) / $C_{21}H_{28}N_3O_3S$ (-)

m/z: 404.1988 (+) / 402.1844 (-)

Retention time: 6.68 min

Confidence level: 3

Proposed chemical structure:

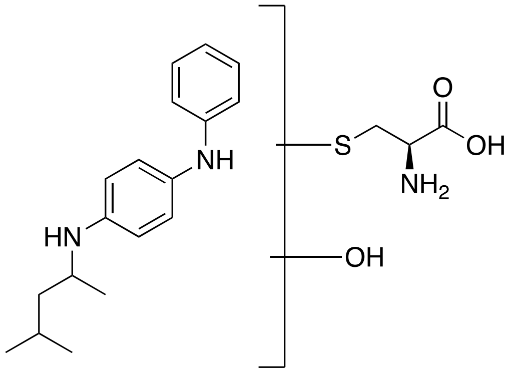

MS/MS spectrum:

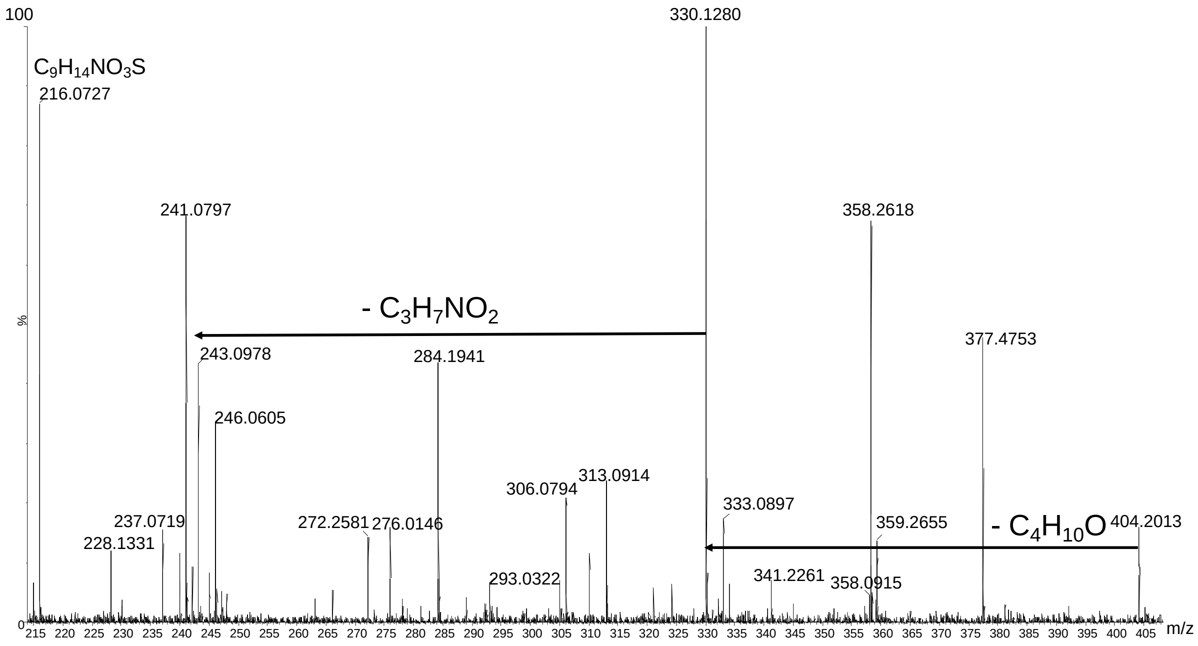

Compound name: 4-HDPA +glucuronide $+CH_2O$ (Acyl)

Chemical formula: $C_{19}H_{22}NO_8$ (+) / $C_{19}H_{20}NO_8$ (-)

m/z: 392.1343 (+) / 390.1188 (-)

Retention time: 6.77 min

Confidence level: 3

Proposed chemical structure:

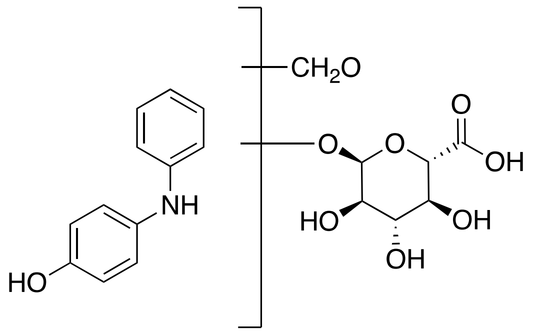

MS/MS spectrum:

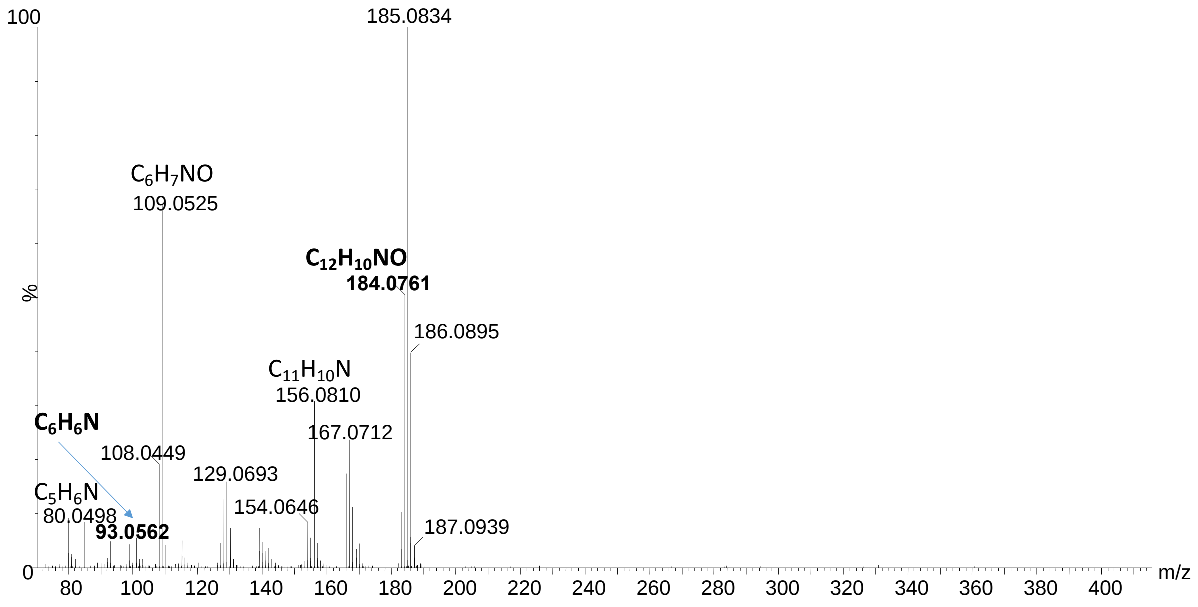

Compound name: 4-HDPA+O+acyl-glucuronide+NAcCys

Chemical formula: $C_{25}H_{31}N_2O_{12}S$ (+) / $C_{25}H_{29}N_2O_{12}S$ (-)

m/z: 583.1590 (+) / 581.1439 (-)

Retention time: 6.89 min

Confidence level: 3

Proposed chemical structure:

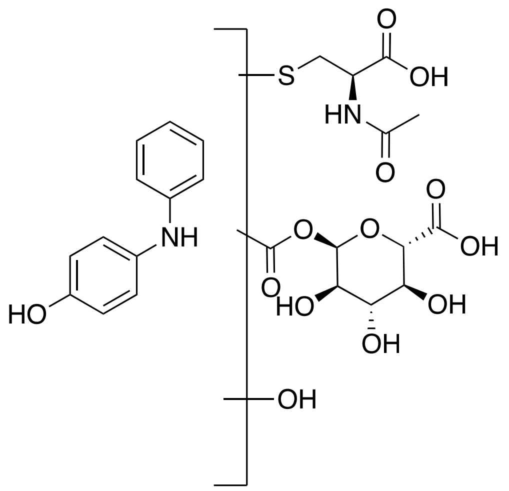

MS/MS spectrum:

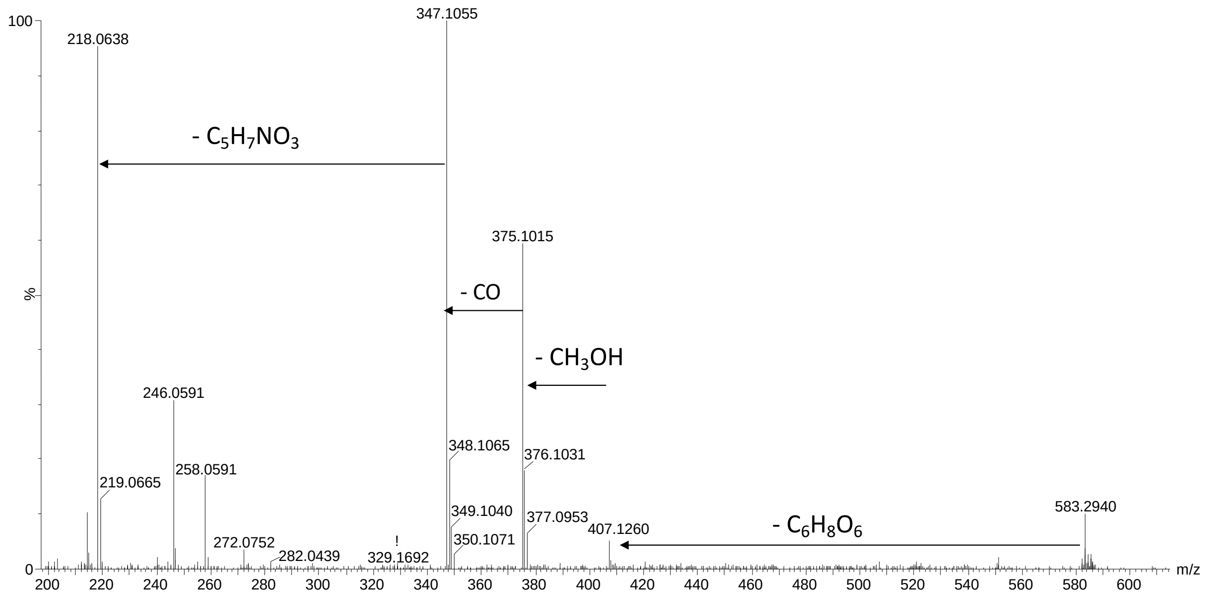

Compound name: 4-HDPA+GSH+NAcCys

Chemical formula: $C_{27}H_{32}N_5O_{10}S_2$ (+) / $C_{27}H_{30}N_5O_{10}S_2$ (-)

m/z: 650.159 (+) / 648.1440 (-)

Retention time: 7.07 min

Confidence level: 3

Proposed chemical structure:

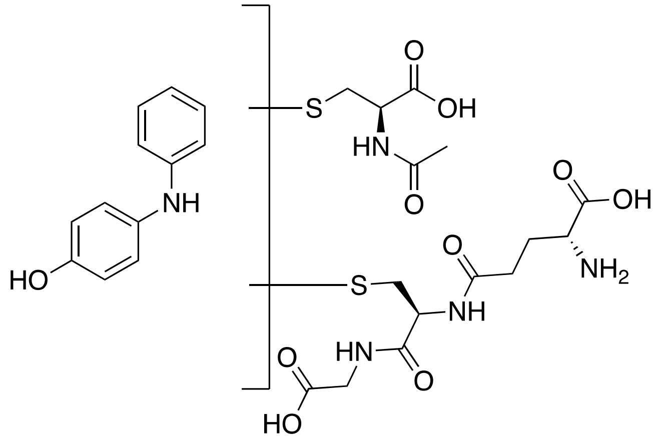

MS/MS spectrum:

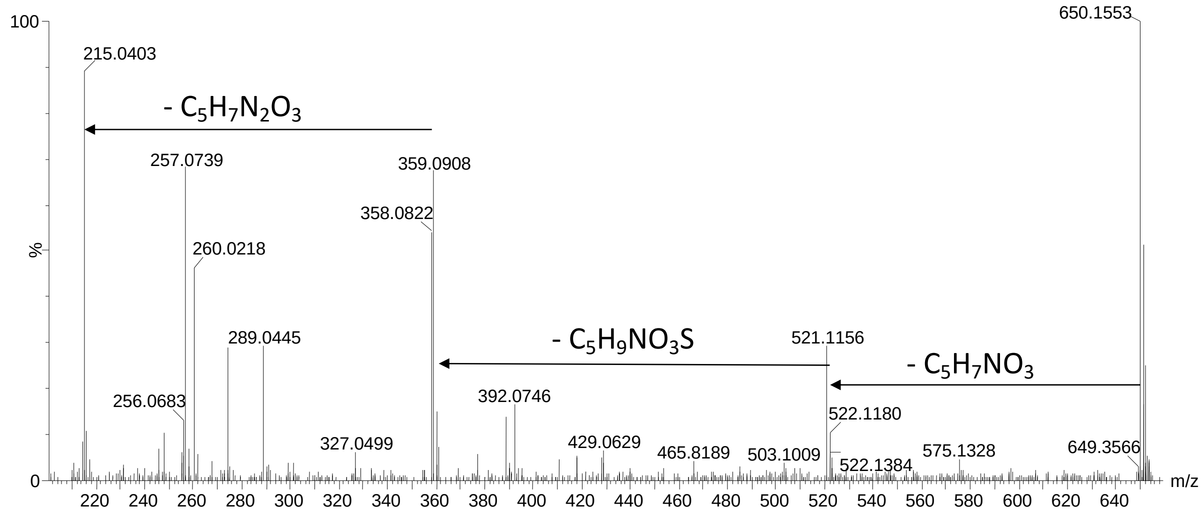

Compound name: 4-HDPA+SO_3

Chemical formula: $C_{12}H_{12}NO_4S$ (+) / $C_{12}H_{10}NO_4S$ (-)

m/z: 266.0480 (+) / 264.0340 (-)

Retention time: 7.08 min

Confidence level: 3

Proposed chemical structure:

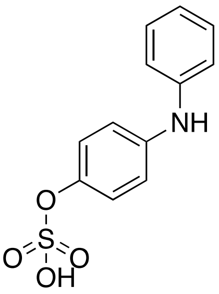

MS/MS spectrum:

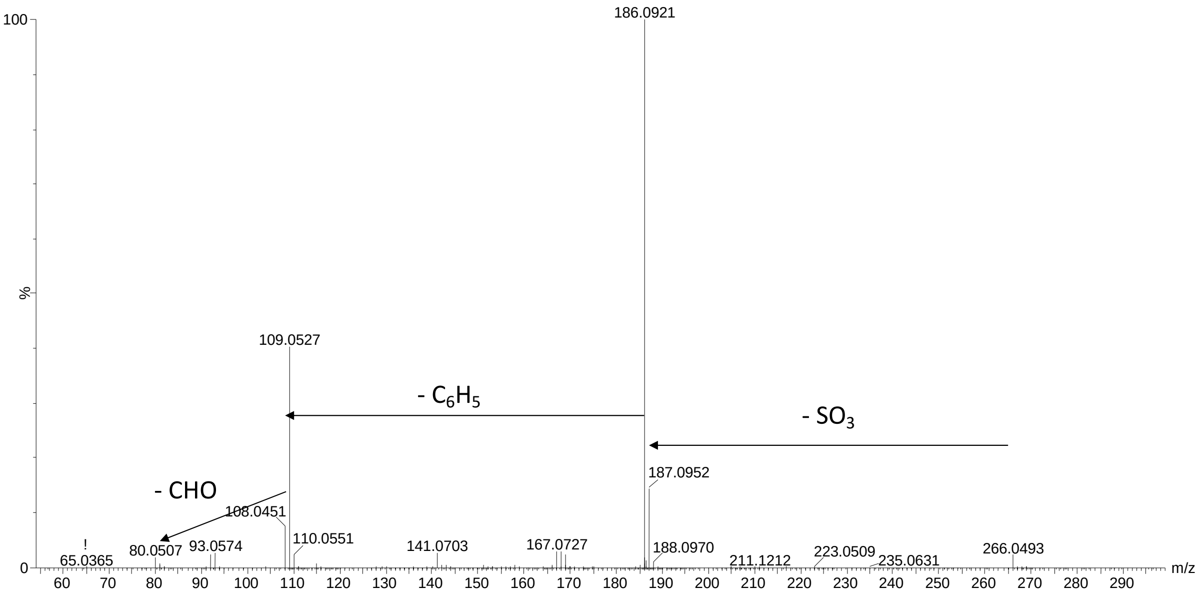

Compound name: 6-PPD+O+NAcCys

Chemical formula: $C_{23}H_{32}N_3O_4S$ (+) / $C_{23}H_{30}N_3O_4S$ (-)

m/z: 446.2105 (+) / 444.1955 (-)

Retention time: 7.71 min

Confidence level: 3

Proposed chemical structure:

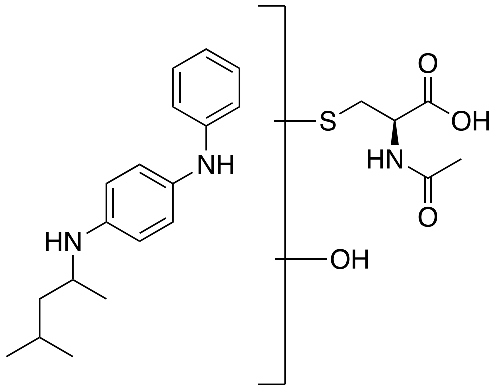

MS/MS spectrum:

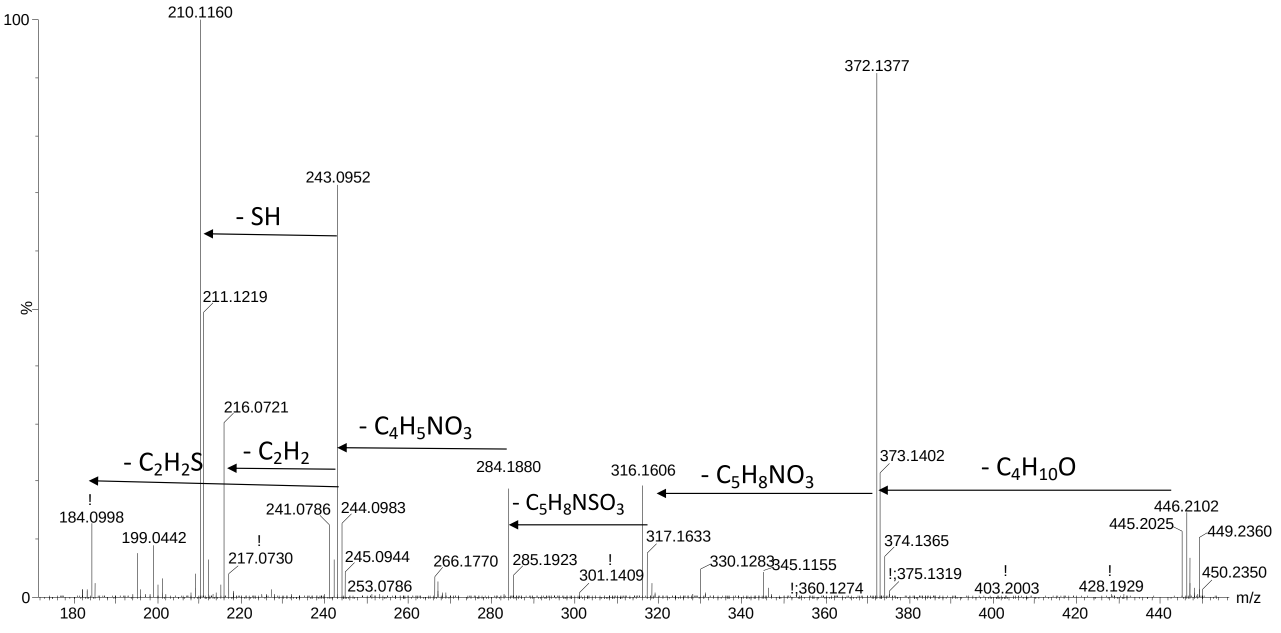

Compound name: 4-HDPA

Chemical formula: $C_{12}H_{12}NO$ (+)

m/z: 186.092 (+)

Retention time: 7.94 min

Confidence level: 1

Proposed chemical structure:

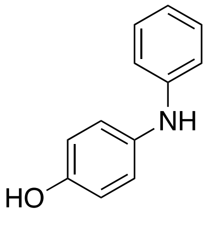

MS/MS spectrum:

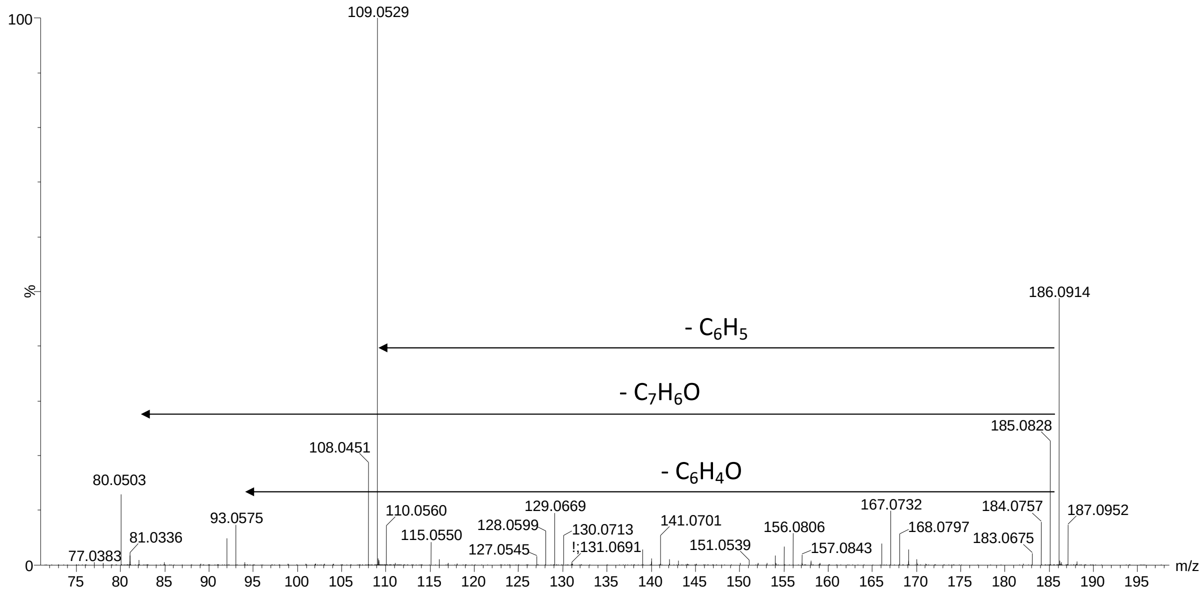

Compound name: 4-HDPA+NAcCys

Chemical formula: $C_{17}H_{17}N_2O_4S$ (+) / $C_{17}H_{15}N_2O_4S$ (-)

m/z: 345.092 (+) / 343.0755 (-)

Retention time: 7.98; 8.18 min

Confidence level: 3

Proposed chemical structure:

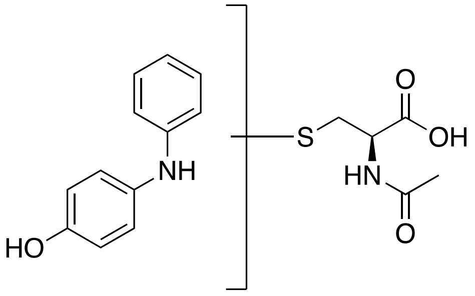

MS/MS spectrum:

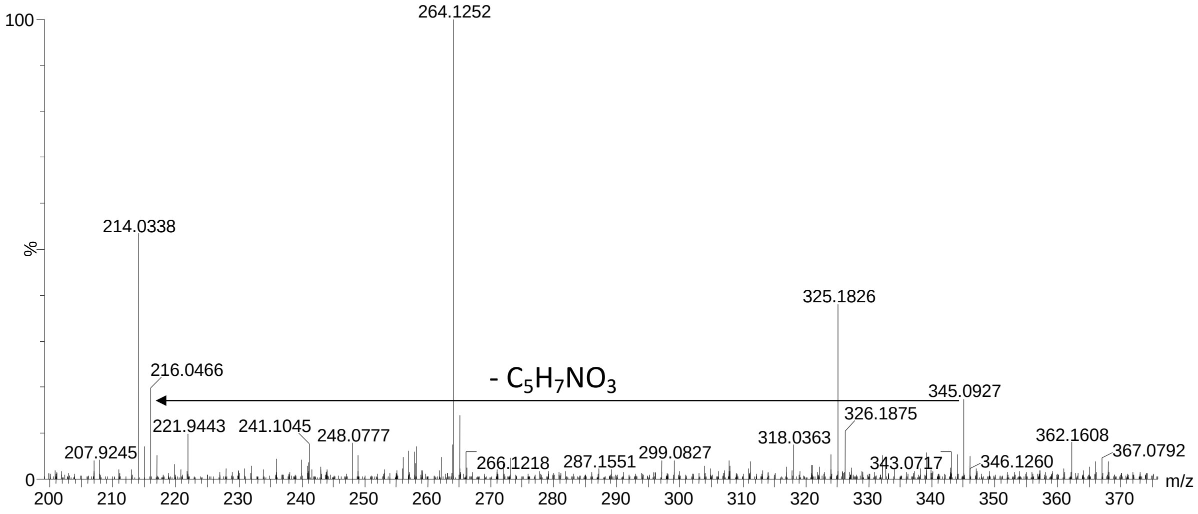

Compound name: 6-PPD+Cys

Chemical formula: $C_{21}H_{30}N_3O_2S$ (+) / $C_{21}H_{28}N_3O_2S$

m/z: 388.2046 (+) / 386.1896 (-)

Retention time: 8.07 min (+) / 8.05 min (-)

Confidence level: 3

Proposed chemical structure:

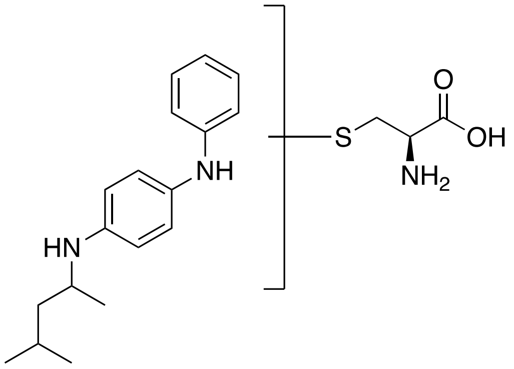

MS/MS spectrum:

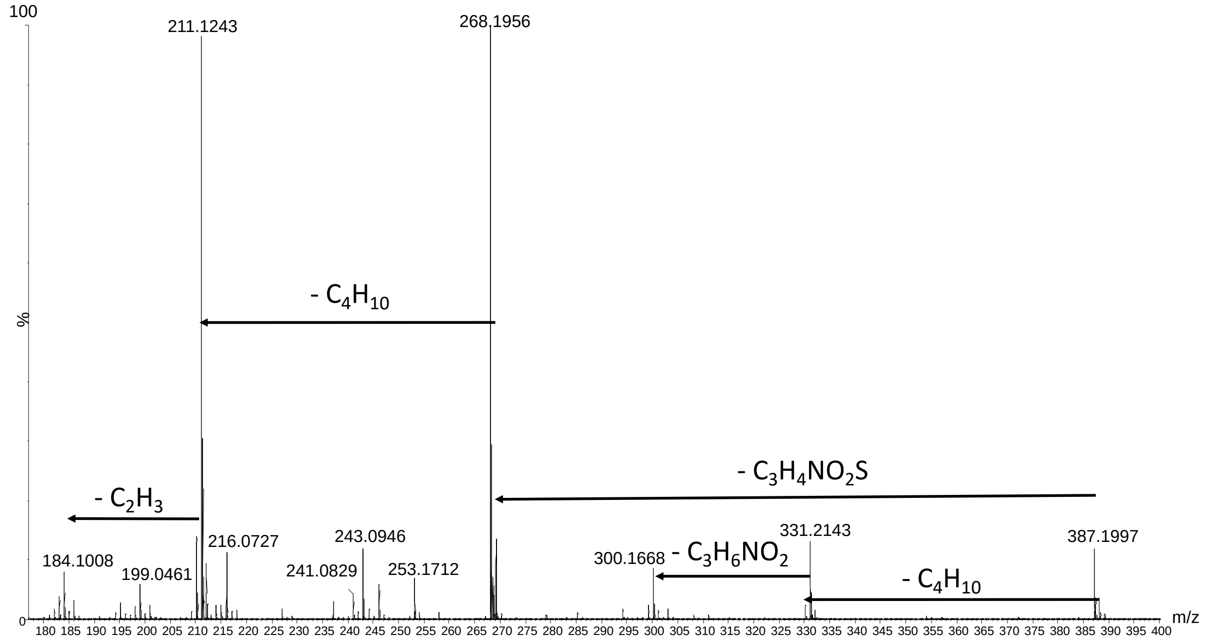

Compound name: 6-PPD+glutathione

Chemical formula: $C_{28}H_{38}N_5O_6S$ (+) / $C_{28}H_{36}N_5O_6S$ (-)

m/z: 572.2549 (+) **/** 570.2386 (-)

Retention time: 8.13 min

Confidence level: 3

Proposed chemical structure:

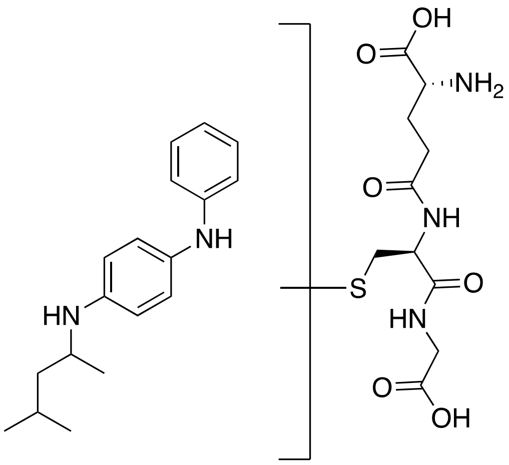

MS/MS spectrum:

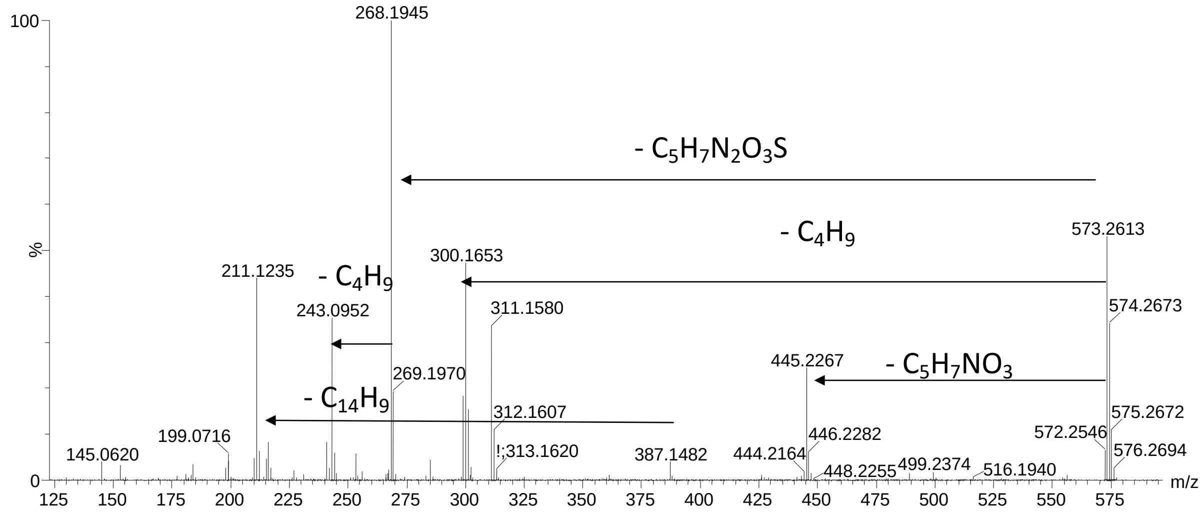

Compound name: 4-HDPA+Cys-Gly+Acetyl

Chemical formula: $C_{19}H_{23}N_2O_6S$

m/z: 407.12600 (+) / 405.1123 (-)

Retention time: 8.32 min

Confidence level: 3

Proposed chemical structure:

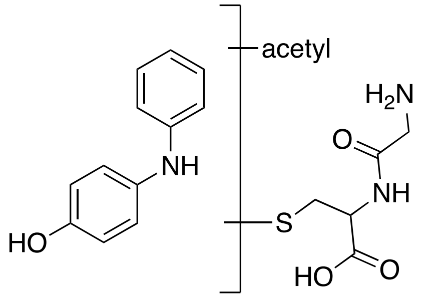

MS/MS spectrum:

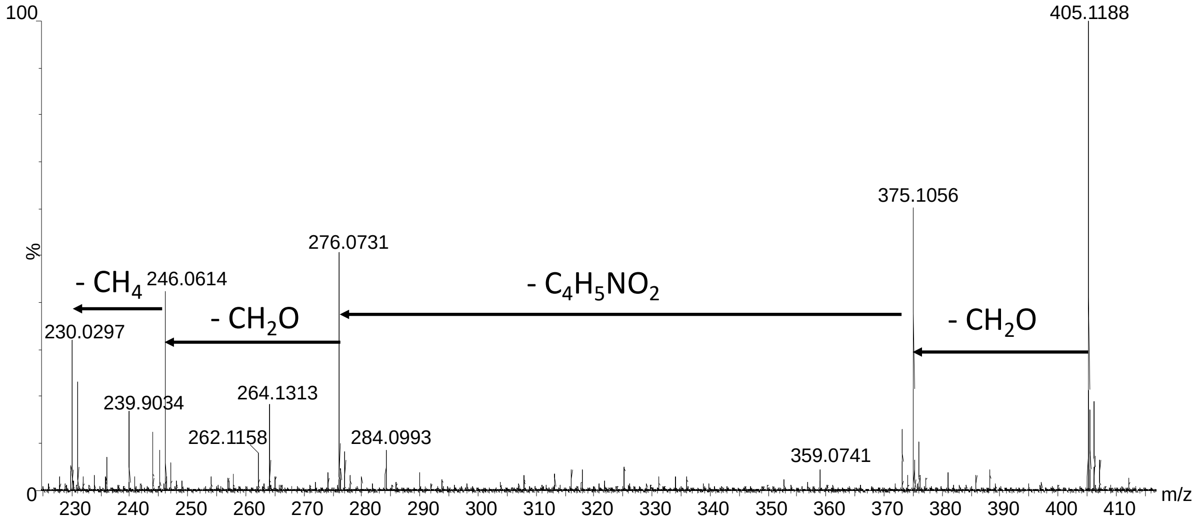

Compound name: 6-PPD

Chemical formula: $C_{18}H_{25}N_2$ (+)

m/z: 269.2072 (+)

Retention time: 8.42 min

Confidence level: 1

Proposed chemical structure:

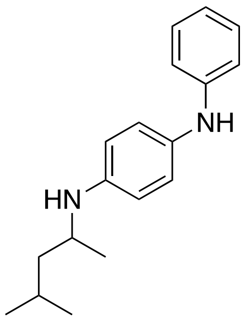

MS/MS spectrum:

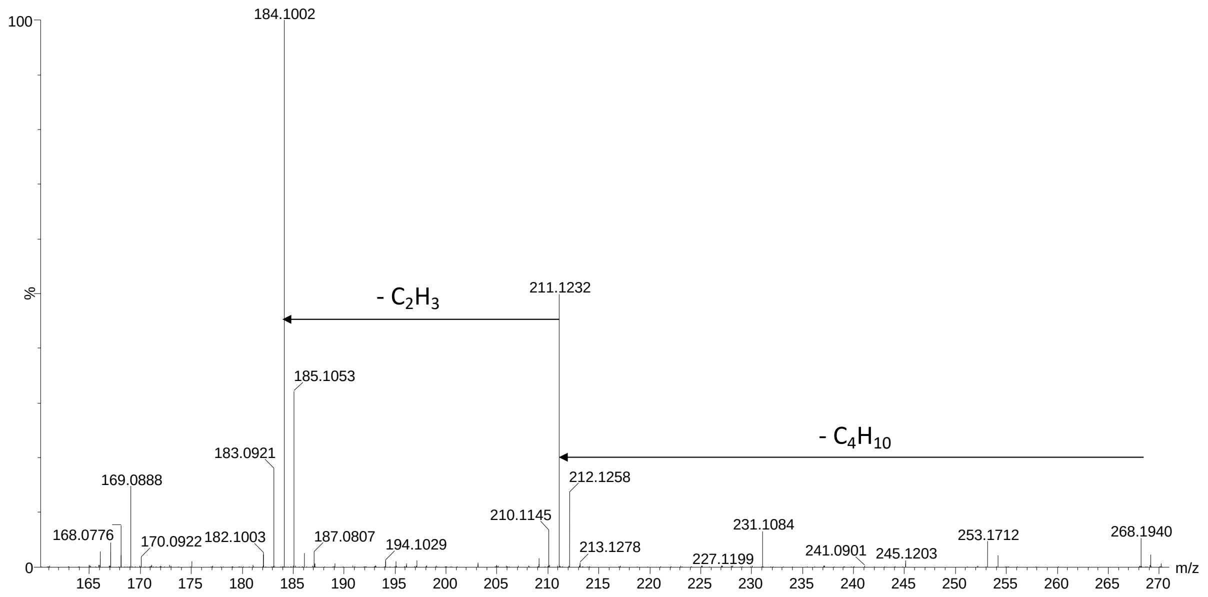

Compound name: 6-PPD+NAcCys

Chemical formula: $C_{23}H_{32}N_3O_3S$ (+)

m/z: 430.2148

Retention time: 8.83 min

Confidence level: 3

Proposed chemical structure:

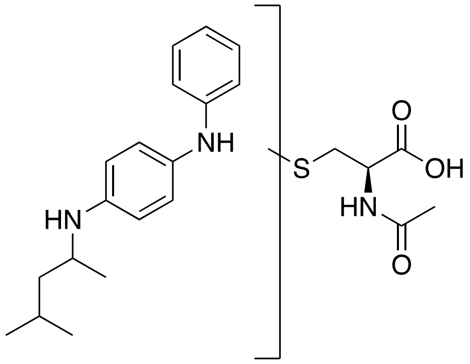

MS/MS spectrum:

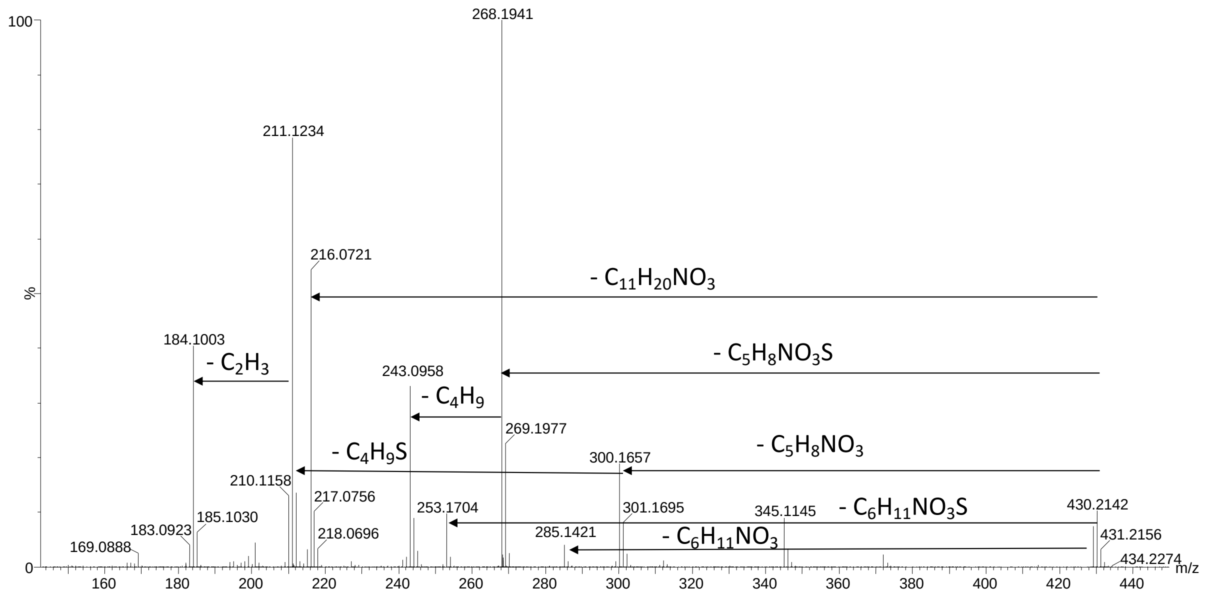

Compound name: 6-PPDQ

Chemical formula: $C_{18}H_{25}N_2$ (+)

m/z: 299.1760 (+)

Retention time: 10.98 min

Confidence level: 1

Proposed chemical structure:

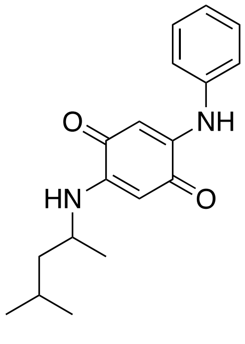

MS/MS spectrum: no fragments detected

Compound name: 6-PPDQ+O+glucuronide

Chemical formula: $C_{24}H_{31}N_2O_9$ (+) / $C_{24}H_{29}N_2O_9$

m/z: 491.2030 (+) / 489.1884 (-)

Retention time: 8.96 min; 8.60 min

Confidence level: 3

Proposed chemical structure:

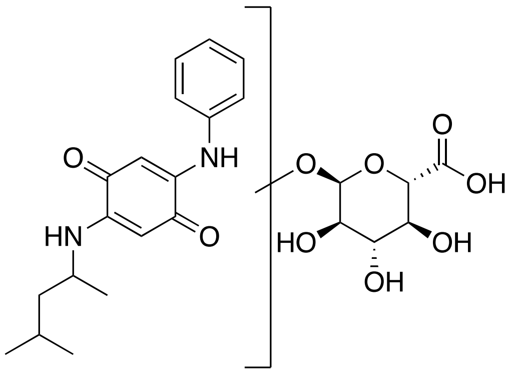

MS/MS spectrum:

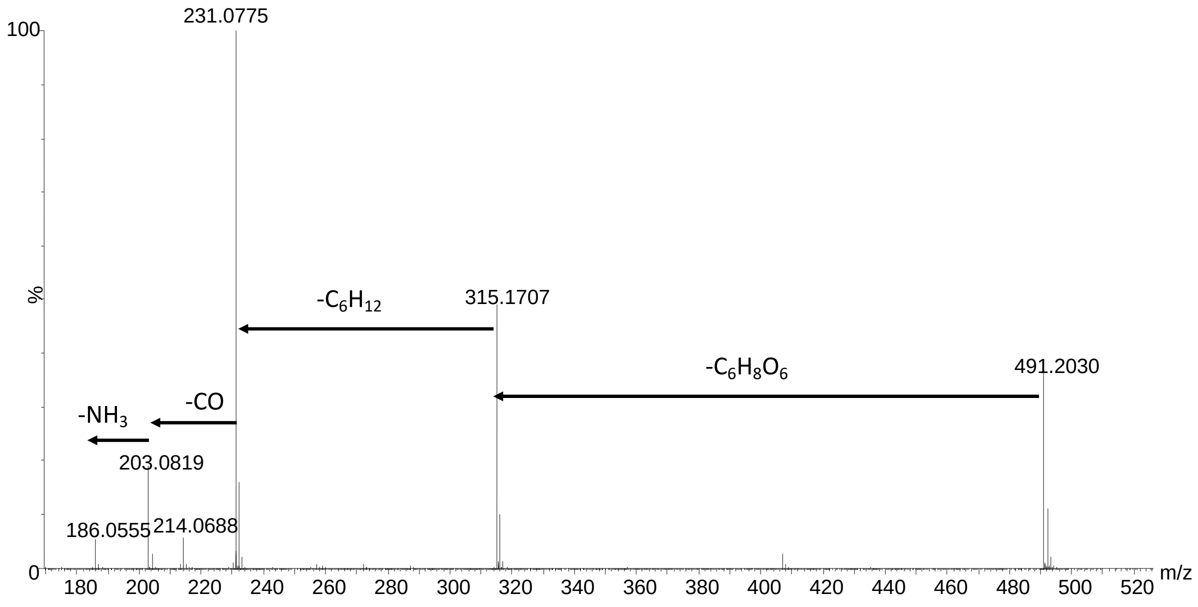

Compound name: 6-PPDQ+O+SO_3

Chemical formula: $C_{18}H_{23}N_2O_6S$ (+) / $C_{18}H_{21}N_2O_6S$

m/z: 395.1263 (+) / 393.1180 (-)

Retention time: 9.47 min (+) / 9.48 min (-)

Confidence level: 3

Proposed chemical structure:

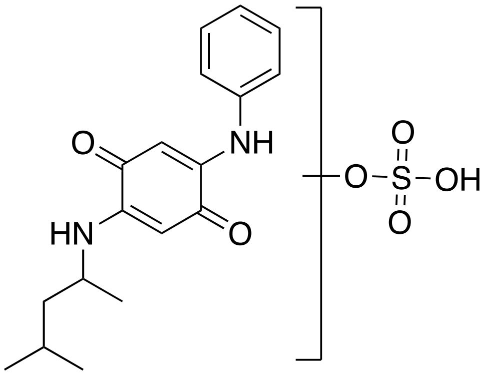

MS/MS spectrum:

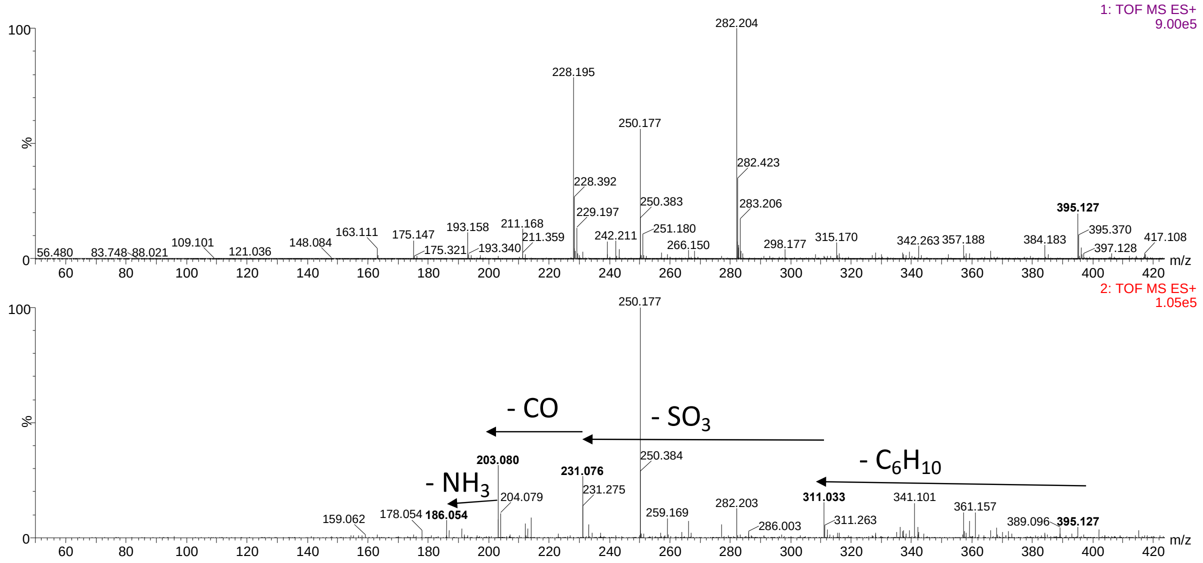

Compound name: 6-PPDQ+2 O+glucuronide

Chemical formula: $C_{24}H_{31}N_2O_{10}$ (+) / $C_{24}H_{29}N_2O_{10}$ (-)

m/z: 507.1978 (+) / 505.1826 (-)

Retention times: 6.83 min; 6.91 min; 7.46 min; 7.6 min

Confidence level: 3

Proposed chemical structure:

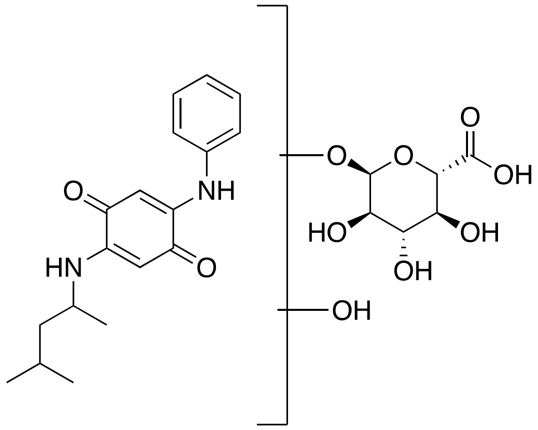

MS/MS spectrum:

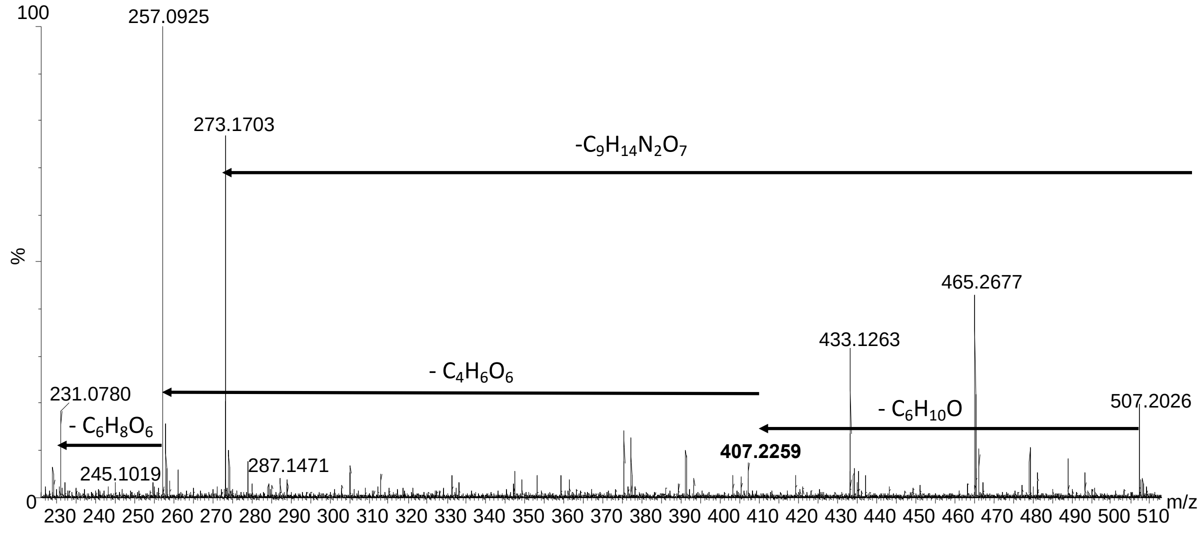

Compound name: 6-PPDQ+2O+SO_3

Chemical formula: $C_{18}H_{23}N_2O_7S$ (+) / $C_{18}H_{21}N_2O_7S$ (-)

m/z: 411.122 (+) / 409.1063 (-)

Retention time: 7.23 min; 7.32 min**;** 9.37 min

Confidence level: 3

Proposed chemical structure:

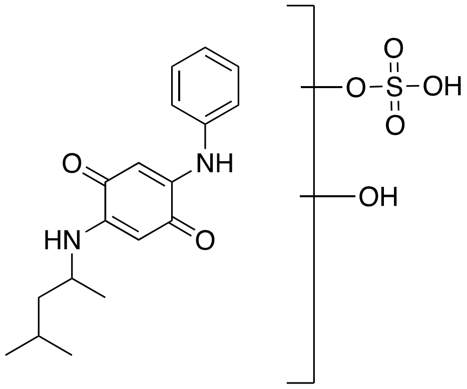

MS/MS spectrum:

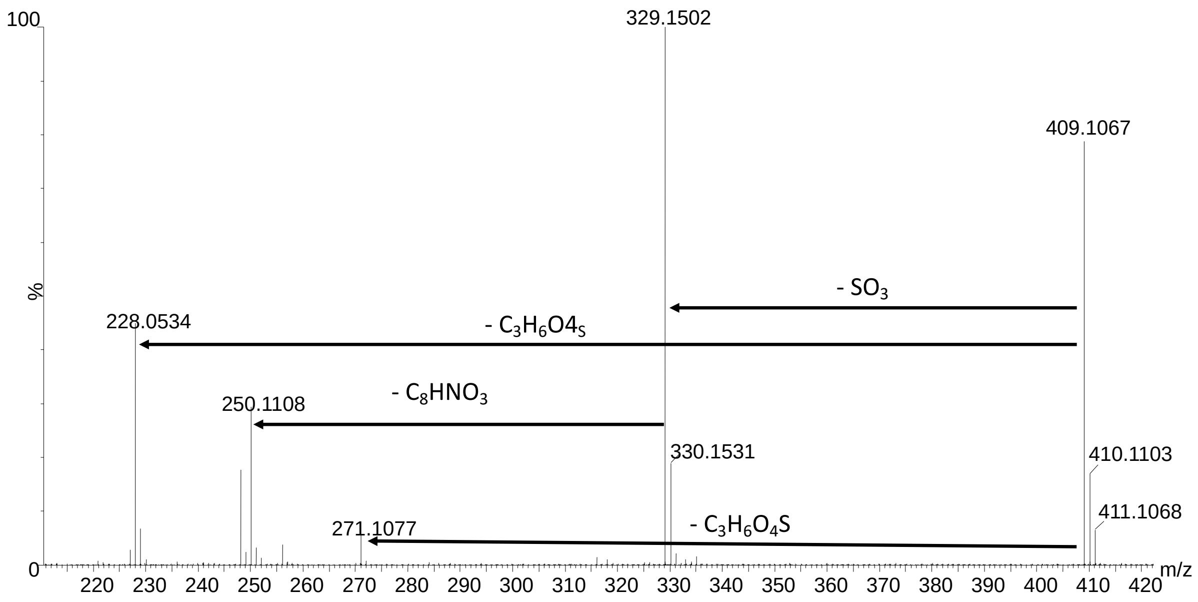

Compound name: 6-PPDQ+O+GSH

Chemical formula: $C_{28}H_{38}N_5O_9S$ (+) / $C_{28}H_{36}N_5O_9S$ (-)

m/z: 620.2386 (+) / 618.222 (-)

Retention time: 8.62 min

Confidence level: 3

Proposed chemical structure:

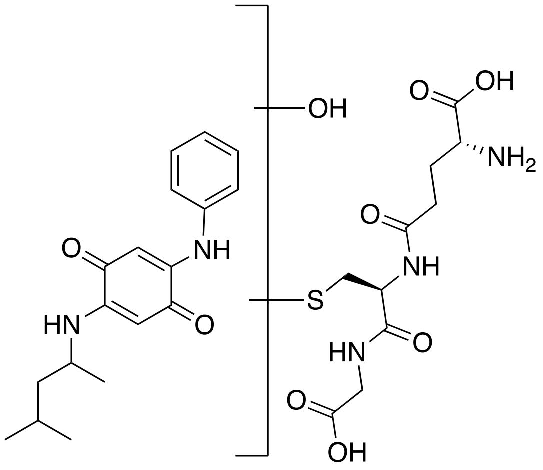

MS/MS spectrum:

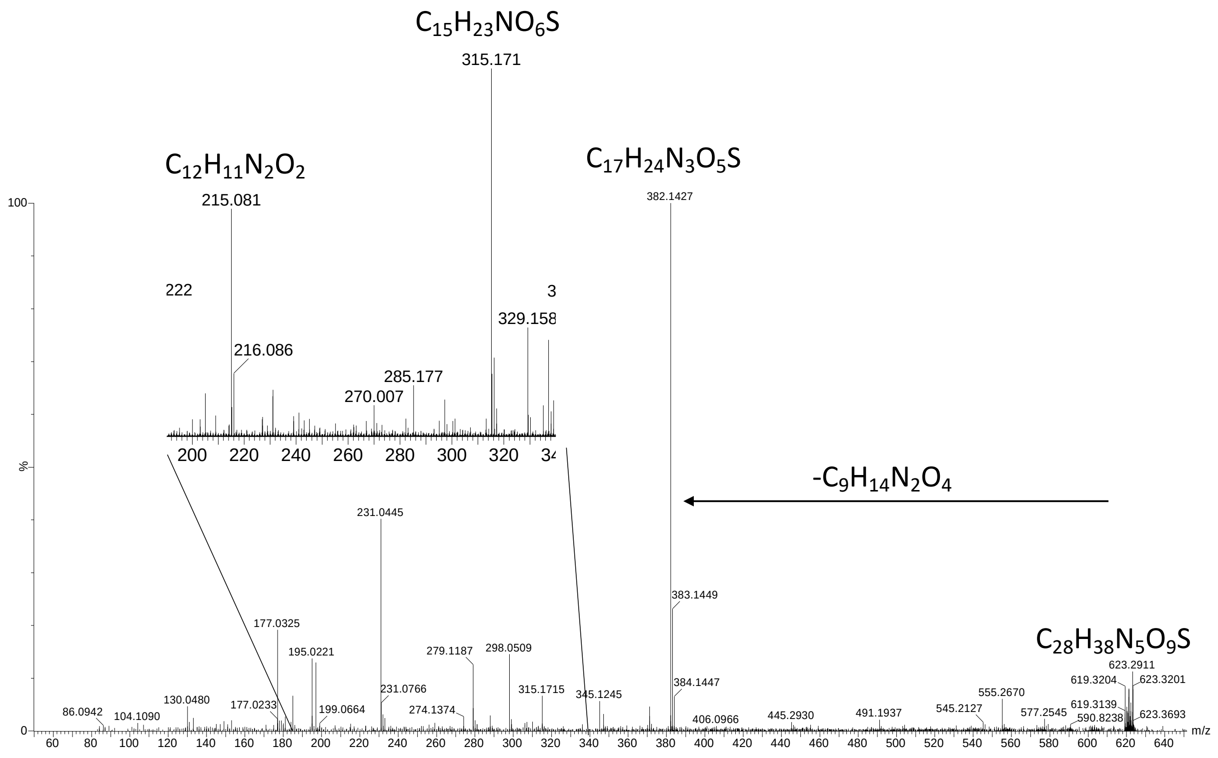

Compound name: 6-PPDHQ+acylglucuronide+NAcCys

Chemical formula: $C_{30}H_{40}N_3O_{13}S$ (+) / $C_{30}H_{38}N_3O_{13}S$ (-)

m/z: 682.2278 (+) / 680.2127 (-)

Retention time: 8.87 min

Confidence level: 3

Proposed chemical structure:

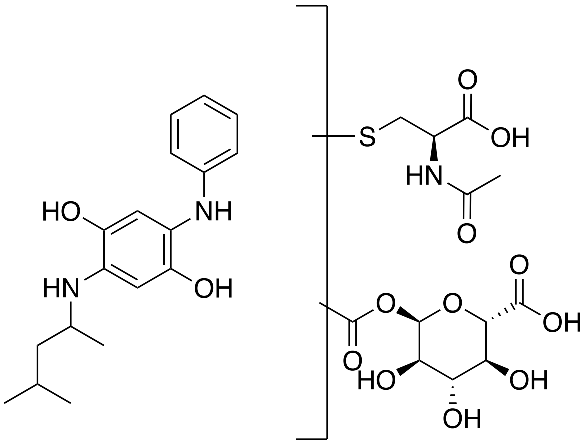

MS/MS spectrum:

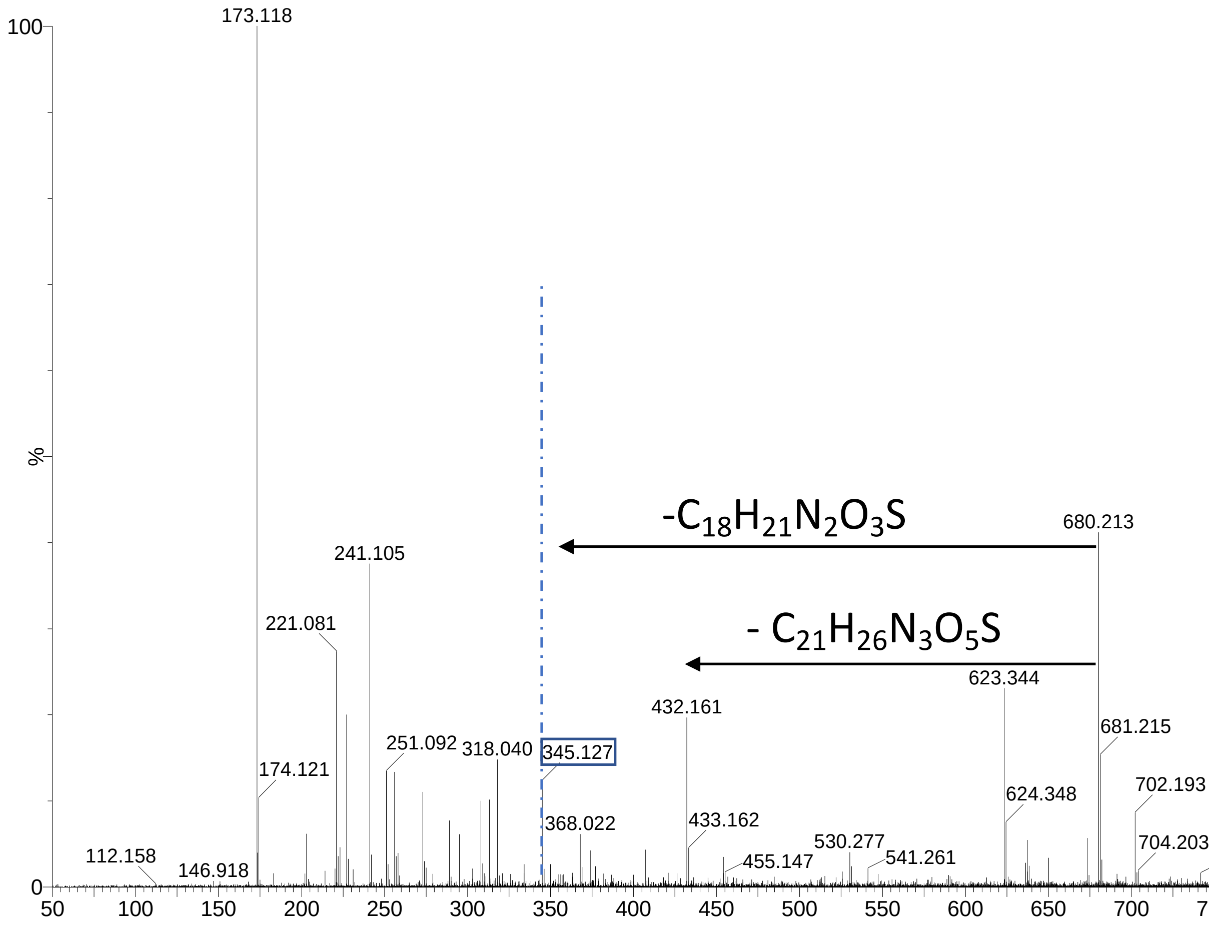

Compound name: 6-PPDQ+2O+CH_3+Cys

Chemical formula: $C_{22}H_{29}N_3O_6S$ (+) / $C_{22}H_{28}N_3O_6S$ (-)

m/z: 464.1835 (+) / 462.1694 (-)

Retention time: 9.03 min

Confidence level: 3

Proposed chemical structure:

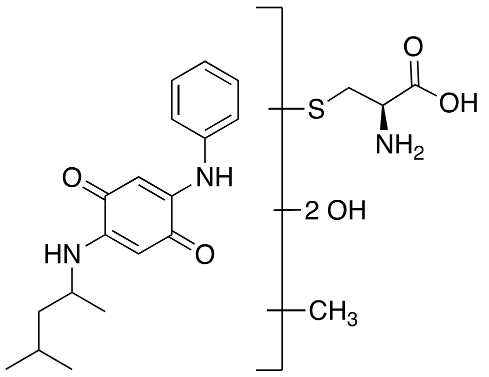

MS/MS spectrum:

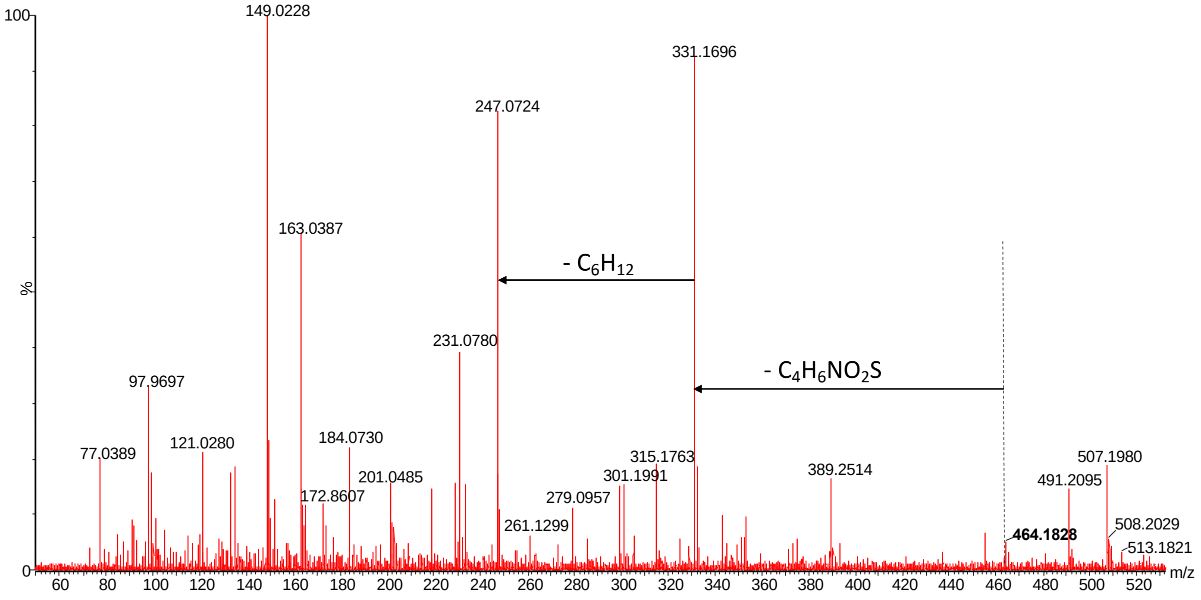

Compound name: 6-PPDQ+O+NAcCys

Chemical formula: $C_{23}H_{29}N_3O_6S$ (+)

m/z: 476.1846 (+)

Retention time: 9.68 min

Confidence level: 3

Proposed chemical structure:

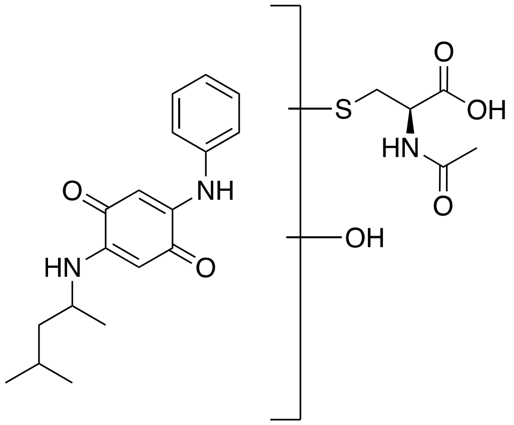

MS/MS spectrum:

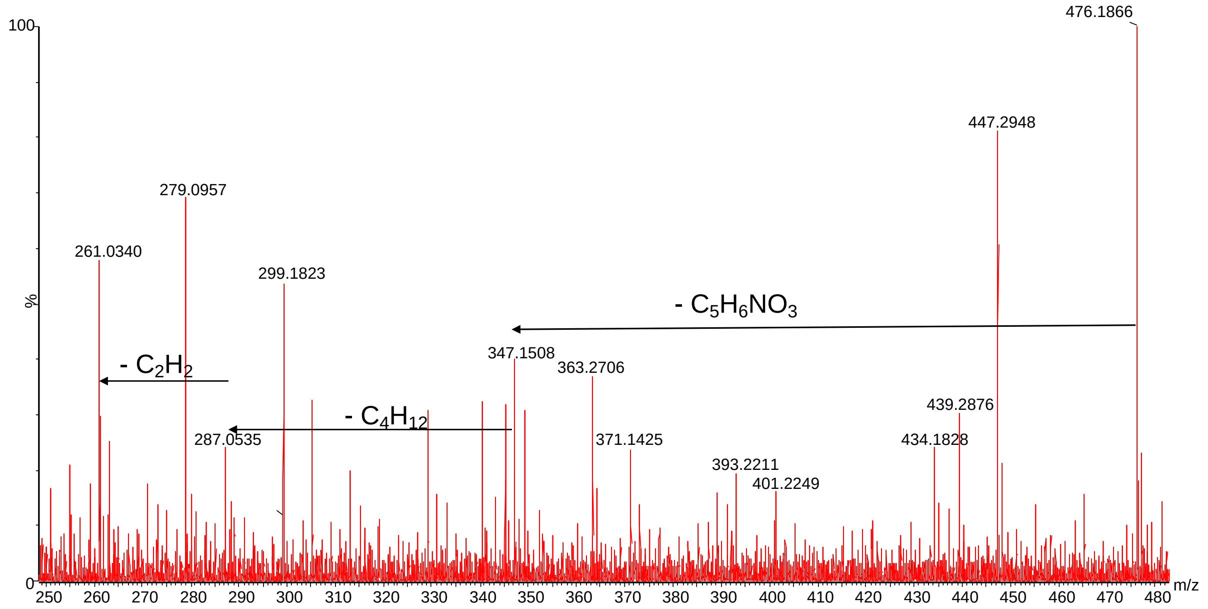

Compound name: 6-PPDQ+O

Chemical formula: $C_{18}H_{23}N_2O_3$ (+)

m/z: 315.1702 (+)

Retention time: 9.99 min

Confidence level: 3

Proposed chemical structure:

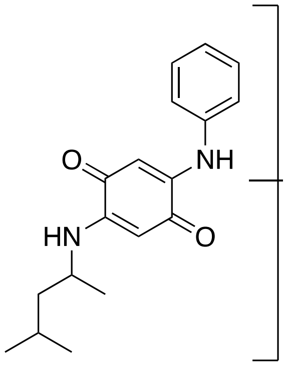

MS/MS spectrum:

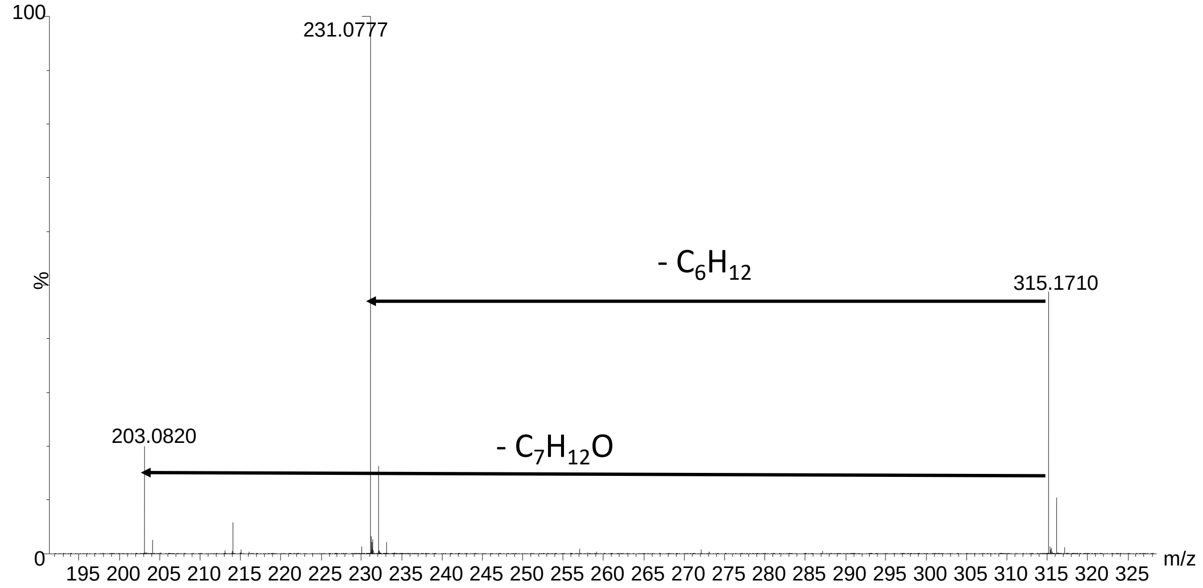

13. References Supporting Information

(1) Varshney, S.; Gora, A. H.; Siriyappagouder, P.; Kiron, V.; Olsvik, P. A. Toxicological Effects of 6PPD and 6PPD Quinone in Zebrafish Larvae. *Journal of Hazardous Materials* **2022**, *424*, 127623. https://doi.org/10.1016/j.jhazmat.2021.127623.

(2) Brox, S.; Ritter, A. P.; Küster, E.; Reemtsma, T. A Quantitative HPLC–MS/MS Method for Studying Internal Concentrations and Toxicokinetics of 34 Polar Analytes in Zebrafish (Danio Rerio) Embryos. *Anal Bioanal Chem* **2014**, *406* (20), 4831–4840. https://doi.org/10.1007/s00216-014-7929-y.

(3) Bittner, L.; Klüver, N.; Henneberger, L.; Mühlenbrink, M.; Zarfl, C.; Escher, B. I. Combined Ion-Trapping and Mass Balance Models To Describe the PH-Dependent Uptake and Toxicity of Acidic and Basic Pharmaceuticals in Zebrafish Embryos (*Danio Rerio*). *Environ. Sci. Technol.* **2019**, *53* (13), 7877–7886. https://doi.org/10.1021/acs.est.9b02563.

(4) Halbach, K.; Ulrich, N.; Goss, K.-U.; Seiwert, B.; Wagner, S.; Scholz, S.; Luckenbach, T.; Bauer, C.; Schweiger, N.; Reemtsma, T. Yolk Sac of Zebrafish Embryos as Backpack for Chemicals? *Environ. Sci. Technol.* **2020**, *54* (16), 10159–10169. https://doi.org/10.1021/acs.est.0c02068.
